# Genomic Approach to Identify Factors That Drive the Formation of Three-Dimensional Structures by EA.hy926 Endothelial Cells

**DOI:** 10.1371/journal.pone.0064402

**Published:** 2013-05-10

**Authors:** Xiao Ma, Markus Wehland, Herbert Schulz, Katrin Saar, Norbert Hübner, Manfred Infanger, Johann Bauer, Daniela Grimm

**Affiliations:** 1 Institute of Biomedicine, Pharmacology, Aarhus University, Aarhus, Denmark; 2 Clinic for Plastic, Aesthetic and Hand Surgery, Otto-von-Guericke-University Magdeburg, Magdeburg, Germany; 3 Max-Delbruck-Center for Molecular Medicine, Berlin-Buch, Germany; 4 Max-Planck Institute for Biochemistry, Martinsried, Germany; Medical University Innsbruck, Austria

## Abstract

Understanding the mechanisms responsible for tube formation by endothelial cells (ECs) is of major interest and importance in medicine and tissue engineering. Endothelial cells of the human cell line EA.hy926 behave ambivalently when cultured on a random positioning machine (RPM) simulating microgravity. Some cells form tube-like three-dimensional (3D) aggregates, while other cells (AD) continue to grow adherently. Between the fifth and seventh day of culturing, the two types of cell growth achieve the greatest balance. We harvested ECs that grew either adherently or as 3D aggregates separately after five and seven days of incubation on the RPM, and applied gene array analysis and PCR techniques to investigate their gene expression profiles in comparison to ECs growing adherently under normal static 1 *g* laboratory conditions for equal periods of time. Using gene arrays, 1,625 differentially expressed genes were identified. A strong overrepresentation of transient expression differences was found in the five-day, RPM-treated samples, where the number of genes being differentially expressed in comparison to 1 *g* cells was highest as well as the degree of alteration regarding distinct genes. We found 27 genes whose levels of expression were changed at least 4-fold in RPM-treated cells as compared to 1 *g* controls. These genes code for signal transduction and angiogenic factors, cell adhesion, membrane transport proteins or enzymes involved in serine biosynthesis. Fifteen of them, with *IL8* (interleukin 8) and *VWF* (von Willebrand factor) the most prominently affected, showed linkages to genes of another 20 proteins that are important in cell structure maintenance and angiogenesis and extended their network of interaction. Thus, the study reveals numerous genes, which mutually influence each other during initiation of 3D growth of endothelial cells.

## Introduction

The inner surface of healthy blood vessels is lined with endothelial cells (ECs) that play an active role in phenomena such as transporting molecules, guiding cell migration, regulating blood pressure and coagulation [1]. In addition, ECs are very important in neoangiogenesis, which occurs *in vivo* during wound healing, placenta formation or tumor neovascularization [2], [3], [4]. In these cases, some endothelial cells of existing vessels start growing. A tip cell is selected and pushed forward by proliferating stalk cells to form a vessel wall [5]. Most of our knowledge about ECs comes from *in vitro* experiments with human umbilical vein endothelial cells (HUVEC) [6]. In addition, permanent cell lines are often used in angiogenesis research [7]. One of the most frequently used and best characterized permanent human vascular EC lines is EA.hy926, which was generated by fusion of HUVEC with the human lung carcinoma cell line A549 [8]. EA.hy926 cells have proven especially useful for studying the formation of new vessels [9].

When we cultured EA.hy926 cells on a random positioning machine (RPM), a device created to simulate microgravity on Earth, adherently growing cells as well as three-dimensional (3D) aggregates were observed [10], [11]. The adherently growing cells maintained a shape comparable to cells that were cultured under normal 1 *g* conditions, but possessed altered molecular features [12]. ECs forming 3D aggregates had detached from the bottom of the culture flasks [10]. The 3D aggregates were columnar and had a central lumen surrounded by one (single layered) or more layers of ECs [11]. They had never been detected under static 1 *g* conditions. Therefore, we concluded that annulling gravitational forces can trigger ECs to form tubes.

Microgravity affects several molecular features of ECs [13]. Even short-term cancellation of gravity (22s) generated by parabolic flights significantly influences signaling pathways [14]. After four and 12 hours of cultivation on the RPM, a number of proteins were up- or downregulated in comparison to control cells and apoptosis was enhanced [10]. During further incubation, apoptosis remained below 30% while the mRNA- and protein levels of several extracellular matrix components and growth-regulating factors changed. After two weeks, a very interesting subtype of 3D-aggregates was observed in the culture supernatants. Its central lumen was surrounded by one layer of ECs. These single-layered tubular structures (TS) resembled the intimas of blood vessels. Characterization of these TS revealed that they might originate from double-row cell assemblies formed between the fifth and seventh days of culture under simulated microgravity, while the percentage of apoptotic cells was about twice as high as in control cell populations at this time [12].

The formation of a blood vessel is accompanied by changes in transcriptional regulation in ECs [15], [16]. Under simulated microgravity, EC exhibit alterations in the expression of various genes and proteins including protein kinase A catalytic subunit, protein kinase C-alpha, and extracellular signal-regulated kinases 1 and 2 [12], [17], [18]. In order to extend our knowledge about changes in gene expression levels that occur when EA.hy926 cells are grown on the RPM simulating microgravity for either five or seven days as compared to control cells incubated under normal laboratory conditions, we applied microarray analysis and quantitative PCR techniques to search for differentially expressed transcripts in cells cultured on the RPM and those incubated in a normal laboratory incubator (1 *g*). Genes found to be changed more than 4-fold on the RPM were compared with genes known to be involved in angiogenesis but altered less than 4-fold in these experiments. The results obtained suggested that IL-8 is driving the 3D growth of ECs in a microgravity-dependent system via a network of genes and proteins involved in cell structure maintenance, while serine biosynthesis is enhanced.

## Materials and Methods

### Random positioning machine

Microgravity was simulated by a desktop RPM manufactured by Dutch Space, an EADS-Astrium company (Leiden, The Netherlands). Under the control of dedicated software, the RPM enables a random 3D positional change in the biological specimen [19]. The samples were mounted close to the center of the platform on the RPM, which was then placed in an incubator under standard cell culture conditions at 37°C and 5% CO_2_. The method was previously published in detail [10], [11], [12].

### Cell culture procedure

Human endothelial EA.hy926 cells [8] were grown in Dulbecco's modified Eagle's medium (Invitrogen, Eggenstein, Germany) supplemented with 10% fetal bovine serum (Biochrom, Berlin, Germany), 100 units penicillin/ml, and 100 µg streptomycin/ml. The cells were grown in 25-cm^2^ culture flasks (Sarstedt, Nümbrecht, Germany) until subconfluent monolayers were formed. After reaching a subconfluency of 70%, the monolayers (10^6^ cells/flask) were randomized into the following study groups: 120 static control cultures (1 *g* static ground controls are cultures kept in the same incubator as the RPM at 37°C); and 120 samples for the simulated microgravity experiments. All flasks were completely filled without air bubbles. RPM cultures were then incubated immediately on the desktop RPM and under static control conditions (1 *g*) according to the experimental design. Every 48 hours, the cell populations were fed by removing 50% of the cell medium with a pipette, carefully avoiding sucking off the floating 3D aggregates or damaging adherent cells, and adding new culture medium. On days five and seven the cells were harvested as previously described in detail [12].

### Morphology of the three-dimensional structures

Immunohistochemical investigation was performed using monoclonal antibodies against laminin (Sigma-Aldrich Chemie, Taufkirchen, Germany). Antigen-antibody complexes were visualized by the streptavidin–biotin method [12], [20].

### RNA isolation

Twenty cell culture flasks from each group were used for RNA extraction. The cells were scraped off using cell scrapers (Sarstedt, Nümbrecht, Germany), transferred to tubes and pelleted by centrifugation (2500×*g*, 10 min, 4°C). The RNeasy Mini Kit (Qiagen, Hilden, Germany) was used according to the manufacturer's instructions to isolate total RNA [12]. RNA concentrations and quality were determined spectrophotometrically at 260 nm using a NanoDrop instrument (Thermo Scientific, Wilmington, DE, USA). The isolated RNA had an A260/280 ratio of >1.5. cDNA designated for quantitative real-time PCR was then obtained with the First Strand cDNA Synthesis Kit (Fermentas, St. Leon-Rot, Germany) using 1 µg of total RNA in a 20-µl reverse transcription reaction mixture. The method was published previously [14], [21].

### Gene-array technique

The endothelial cell line EA.hy926 was cultivated for five and seven days on the RPM and in parallel five and seven days under normal conditions (1 *g* static controls). During culture on the RPM, some adherent (AD) cells detached to form freely floating 3D aggregates (tubular structures, TS) in the culture medium. Both AD and TS were collected separately for RNA extraction. Each time, four independent RNA preparations from the resulting three different conditions were processed and hybridized using the Illumina HumanWG-6_V2_0_R3_11223189_A array. The resulting profiles had quantile normalization without background correction using the BeadStudio Gene Expression Module v3.3.7. After exclusion of low or unexpressed genes (maximum detection *p*-value >0.05), the arrays were checked for outliers in samples using principal component analysis (PCA), a correlation dispersion matrix and normalized Eigenvector scaling. No outliers were detected. Probes and samples were analyzed for significant expression differences using a two-way ANOVA approach differentiating between the conditions 1 *g*, AD and 3D on the one hand and the time points five days and seven days on the other. Multiple testing correction of the test statistic was performed using the Benjamini Hochberg FDR procedure [22]. Probes that underwent a 5% FDR between the three conditions, the time-course or its interaction and that exceeded a two-fold difference between any of the six conditions were selected as differentially expressed. Individual profile data were separated by k-means clustering using an average Euclidean distance function and k = 9 according to a local minimum in the Davies Bouldin k estimation [23]. Clustering was performed after normalization of probes to a mean of zero and a standard deviation of one. The nine resulting k-mean clusters were further investigated by functional enrichment using g:Profiler [24] with a simulation-based analytical threshold for significance estimation. Test statistics and clustering were performed using the Partek Genomic Suite version 6.4.

### STRING analysis

Physical and functional interactions between proteins were determined using the STRING platform [25], [26] using a low confidence score of 0.15 for all but two exceptions. With respect to the high number of genes in the STRING analyses over combined clusters, proteins were preselected using a high confidence score (0.9). Resulting singletons were rejected before network visualization using a low confidence score of 0.15.

### Quantitative real-time PCR

Quantitative real-time PCR [12], [14], [21] was used to determine the expression levels of the genes of interest. The Primer Express® software was utilized to design appropriate primers with a T_m_ of about 60°C (Table S1). The primers were synthesized by TIB Molbiol (Berlin, Germany). All assays were run on a 7500 Fast Real-Time PCR System using the Fast SYBR®Green PCR Master Mix (both Applied Biosystems, Darmstadt, Germany). The reaction volume was 25 µl, including 1 µl of template cDNA and a final primer concentration of 300 nM. PCR conditions were as follows: 20 s at 95°C, and then 40 cycles of 3 s at 95°C and 30 s at 60°C, followed by a melting curve analysis step (temperature gradient from 60°C to 95°C with +0.3°C per cycle). If all amplicons showed a single T_m_ similar to the one predicted by the Primer Express software, the PCR reactions were considered specific. Every sample was measured in triplicates and we utilized the comparative C_T_ (DDC_T_) method for the relative quantification of transcription levels. 18S rRNA was used as a housekeeping gene to normalize our expression data.

### Statistical analysis

Statistical analysis was performed using SPSS 16.0 (SPSS, Inc., Chicago, IL, USA). All data are expressed as means ± standard deviation (SD). Differences were considered significant at the level of *p*<0.05, employing the Mann-Whitney U-test.

## Results

**Figure 1:**
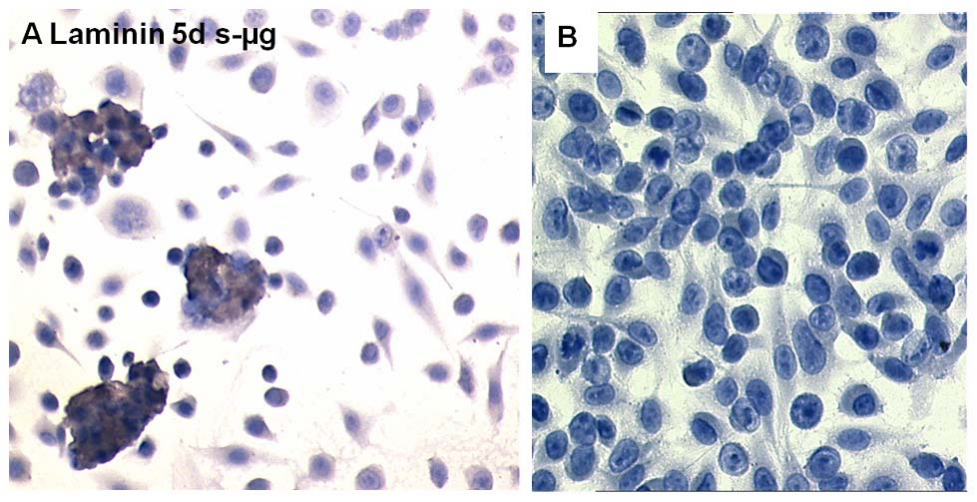
Immunocytochemistry. (A) Laminin-immunocytochemistry of EA.hy926 cells cultured for five days on the RPM. (B) Laminin-immunocytochemistry of EA.hy926 cells cultured for five days in a normal laboratory incubator.

EA.hy926 cells were grown under normal laboratory conditions (60 culture flasks) and on the RPM (60 culture flasks) for five and seven days (n = 30/time point), respectively. After five days on the RPM, a considerable number of cells had detached from the bottoms of the culture flasks. Some of the detached cells had formed small, oblong three-dimensional aggregates that were laminin-positive (Fig. 1A). By this time point, a nearly confluent monolayer had developed in a normal laboratory incubator under 1 *g* gravity (Fig. 1B). Harvesting normal monolayer cultures (Fig. 1B), the cells were scraped off and collected. During the harvest of RPM cultures (Fig. 1A), aggregates and detached cells were collected together with the culture medium first. Then the remaining adherent cells were scraped off and collected. In this way, we obtained six populations of EA.hy926 cells (Table 1). Each cell population was divided into aliquots, of which one was used for microarray analysis and another one for PCR.

**Table 1:** Time periods and conditions of culturing the various EA.hy926 cell populations.

*Time period*	*Condition*	*Kind of growth at harvest*	*Referred to as in text*
5 days	1g	adherent	5d 1g
5 days	RPM	adherent	5d AD
5 days	RPM	3D aggregates	5d 3D
7 days	1g	adherent	7d 1g
7 days	RPM	adherent	7d AD
7 days	RPM	3D aggregates	7d 3D

After harvest, each population was divided and aliquots were used for gene array technique or quantitative real-time PCR; 1 *g*: incubation in a normal laboratory incubator; RPM: incubation under simulated microgravity on the Random positioning machine; d: days.

### Microarray analysis

**Figure 2:**
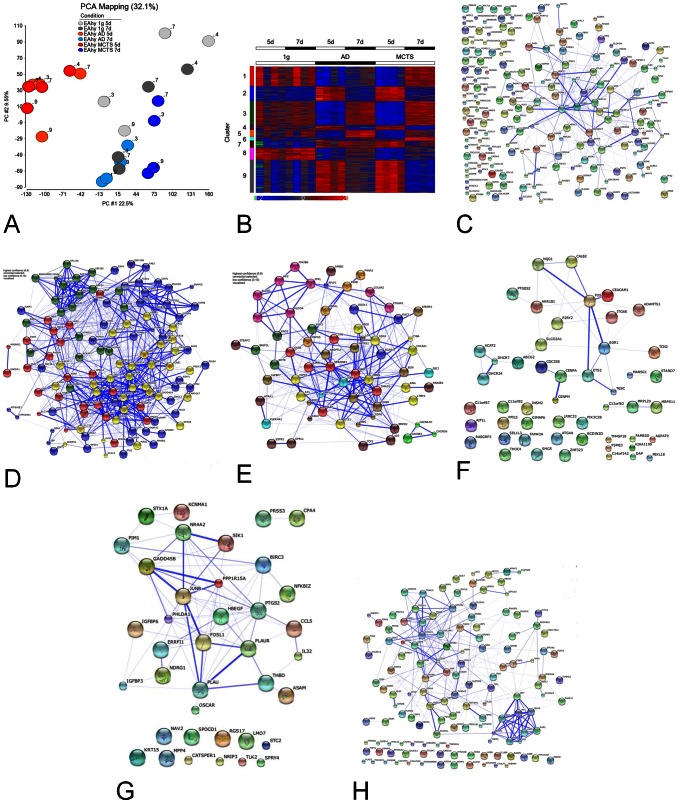
Microarray Analysis. (A) The first two PCs of the PCA of 31,991 expressed transcripts describe 32.1% of the dataset variance. The five-day RPM samples showed a separation from 1 *g* controls and seven-day RPM samples in PC #1 (22.5% of the variance). (B) K-mean clustering of the 1625 significantly regulated probes. The color scale represents upregulation (red) or downregulation (blue) of probes. Outliers are marked in orange (upregulated) or green (downregulated). **(**C–H) STRING visualization of known and predicted physical and functional interactions between the proteins organized in cluster 1 (C), clusters 2, 7 and 9 (D), clusters 3 and 5 (E), cluster 4 (F), cluster 6 (G) and cluster 8 (H).

Using the Illumina HumanWG-6_V2_0_R3_11223189_A array was a first approach to finding genes that are expressed differently depending on the different gravitational conditions under which the cells had grown. Of 31,991 possible transcripts, 1,625 met the criteria of a False Discovery Rate (FDR) below 5% and an expression difference of at least two-fold between any of the tested conditions. These genes were defined as differentially expressed. Biological functions retrievable from the gene ontology database (GO) could be assigned to 1,174 products of the 1,625 differentially expressed transcripts. Several of these functions are related to vessel formation, others to apoptosis, cell structure maintenance, cell migration, and cell adhesion (Table S2). Obvious transient transcriptome differences in the five-day, RPM-treated samples were detected by principal component analysis (PCA) (Fig. 2A). Separating the 1,625 significantly differentially expressed individual profile data by k-means clustering resulted in 9 clusters (Fig. 2B).

### Cluster 1

A widely unspecific set of 163 genes recognized by g:Profiler and 182 proteins recognized by STRING analysis are organized in cluster 1 and characterized by upregulation in seven-day 3D cells, a heterogeneous expression profile in the five-day and seven-day controls and downregulation in AD cells (Fig. 2B). Only a few GO biological processes were detected as significantly enriched: signal transduction (53 genes, GO:0007165, *p* = 6.04×10^−6^) and regulation of phosphorus metabolic processes (19 genes, GO:0051174, *p* = 3.98×10^−6^). In addition, we found single genes that are involved in angiogenesis (*CALD1*, *PNPLA6*, *MAPK11*, *THY1*), apoptosis (*IL10*, *MAP3K11*, *NOX4*, *BIRC3*), extracellular matrix organization (*NFKB2*) or actin cytoskeleton organization (*INF2*) (Fig. 2C).

### Clusters 2, 7 and 9

These clusters represent genes that are upregulated in both AD and 3D cells by day five on the RPM (Fig. 2B). In all three clusters, general metabolic processes were significantly enriched; for example, GO:0044281 (small molecule metabolic processes) with *p* = 2.42×10^−6^ and 38 genes in cluster 2, and GO:0008152 (metabolic processes) with *p* = 4.03×10^−6^ and 35 genes with *p* = 2.90×10^−8^ and 177 genes in clusters 7 and 9, respectively (Fig. 2D). An increase in general expression activity is indicated by increases in expression of DNA-dependent RNA polymerase (*POLR2L*), of exportin (*XPOT*) (which mediates the nuclear export of aminoacylated tRNAs) of ribosomal proteins (*RPL6, RPL9, RPL10a, RPL29,* and *RPLP0*) and the tRNA synthetases for serine (*SARS*), methionine (*MARS*), tyrosine (*YARS*), isoleucine (*IARS*), and glycine (*GARS*). Moreover, especially in clusters 2 and 7, we found more genes involved in angiogenesis (*HIF1A, IL8*), apoptosis (*SFN, BECL2L1, GLRX2, IL1A, IL6*), extracellular matrix organization (*MMP10*), and cytoskeleton organization (*TUBB6, ABLIM1*).

### Clusters 3 and 5

Clusters 3 and 5 are characterized by day-five downregulation in AD and 3D samples (Fig. 2B). The enrichment analysis indicated that these clusters are characterized by genes involved in anatomical structure development (GO:0048856, 79 genes, *p* = 2.00×10^−9^ in cluster 3 and 29 genes, *p* = 6.98×10^−7^ in cluster 5) as well as in cell-cell and cell-environment interactions (GO:0050896: response to stimulus, 121 genes, *p* = 1.61×10^−8^; GO:0007155: cell adhesion, 31 genes, *p* = 3.23×10^−8^; GO:0007154: cell communication 91 genes, *p* = 5.51×10^−9^, all cluster 3). Cluster 3 includes one half of the regulated collagens (*COL13A1, COL4A5, COL5A1, COL6A1,* and *COL6A2*), and both clusters include a multitude of angiogenic genes (for example, *ENG* in cluster 3 and *CAV1* and *CAV2, SERPINE1* and *MMP2* in cluster 5) (Fig. 2E).

### Cluster 4

Cluster 4 is characterized by an upregulation of gene expression by day five and a downregulation by day seven in AD and 3D samples cultured on the RPM (Fig. 2B). No significant enrichment of a GO biological process was found, but single genes involved in angiogenesis (*CEACAM1*), apoptosis (*DAP, DHCR24, FOS, PSME3*), and adhesion (*ITGAE*) belong to this cluster (Fig. 2F).

### Cluster 6

Cluster 6 includes genes upregulated in 3D cells by day seven on the RPM (Fig. 2B). The most significantly enriched biological process is programmed cell death (GO:0016256, 14 genes, *p* = 7.78×10^−7^). Some of the other processes comprising a relevant number of genes include cell communication (GO:0007154, 21 genes, *p* = 7.55×10^−6^) and locomotion (GO:0040011, 12 genes, *p* = 3.81×10^−7^) (Fig. 2G).

### Cluster 8

The genes in cluster 8 are generally downregulated (with an emphasis on day five) in AD and 3D samples and have some similarities in their expression patterns to genes from clusters 3 and 5 (Fig. 2B). Again, anatomical structure development (GO:0048856, 44 genes, *p* = 1.74×10^−6^) and angiogenic processes (GO:0001525, 11 genes, *p* = 2.79×10^−6^) are significantly enriched, including such genes as *COL4A1*, *COL4A2*, *COL5A2*, *COL18A1*, *EGFL7*, *EPHB4*, *NOTCH1*, *PTPRB*, *ROBO4*, *SOX18*, *VASH1*, and *ADAM15* (Fig. 2G). In addition, genes regulating cell adhesion (GO:0007155, 22 genes, *p* = 1.81×10^−8^) and the extracellular matrix (GO:0031012, 17 genes, *p* = 2.98×10^−10^) are also prominently represented (Fig. 2H).

### Quantification of differences

Looking at each individual cluster (Fig. 2B), no major differences between five- and seven-day samples of the 1 *g* control groups were observable. In contrast and corresponding to the PCA results (Fig. 2A), we could identify a strong overrepresentation of transient expression differences in the five-day, RPM-treated samples, where both AD and 3D samples showed a similar pattern (Fig. 2B). The similarity of the gene profiles of AD and 3D cells in five-day RPM treated samples is also notable because the expression of only two genes (*IFIT1, CTGF)* differs by a factor of two, while regarding the seven-day RPM treated samples, 3 genes (*NFKBIZ, NR4A2, FOS)* in 3D cells are more than three-fold upregulated as compared to the corresponding AD cells, and the expression of another 30 genes including *MMP2, MMP9,* and *MMP10* as well as *FOSL, PRDX3* and *TNRSF25* in the 3D and AD cells differs by a factor of two (Table S2).

The overrepresentation of transient expression differences in the five-day, RPM-treated samples (Fig. 2B) relates not only to the number of genes being differentially expressed in comparison to 1 *g* cells, but also to the degree of change regarding distinct genes. Most noticeable is the *IL8* gene. After 5 days on the RPM, the expression of this gene is 6-fold higher in 3D cells and 4-fold higher in AD cells as compared to 1 *g* cells. Further genes strongly affected by incubating the cells on the RPM are summarized in Table 2. It clearly shows that more genes exhibited greater than two-fold changes in expression levels in five-day RPM-treated cells than in seven-day RPM treated cells as compared to 1 *g* cells, respectively. Most of the genes showing large differences in expression code for signal transduction factors (*ASAP3, IFIT1*, *CBS*, *GNG10, STC1).* But major changes in genes for angiogenic factors (*ANGPTL4, IL-8, HMOX1, LOX),* cell adhesion (*VWF, SPP1, ITGB4*) and membrane transport (*SLCO4A1*, *SLC2A1, SLC3A2)* proteins also became obvious (Table 2). Interestingly, two genes were observed which code for enzymes involved in transforming pyruvate to serine (*PHGDH, PSAT1).*

**Table 2:** Number of genes, whose expression was changed more than twofold during incubation on the RPM.

*Fold change*	*5d AD vs. 1g*	*5d 3D vs. 1g*	*7d AD vs. 1g*	*7d 3D vs. 1g*
6 fold	–	1^1^	–	1^2^
5 fold	4^3^	7^4^	–	2^5^
4 fold	13^6^	11^7^	1^8^	–
3 fold	28	42	4	8
2 fold	264	346	33	51

1 *IL-8 (+); ^2^ VWF (−); ^3^SERPINE2 (+), SLCO4A1 (−), PHGDH (+), SEMA4B (−); ^4^ ANGPTL4 (−), SPP1 (+), SERPINE2 (+), SLCO4A1 (−), TSC22D1 (+), MMP10(+), PHGDH (+); ^5^ IL-8 (+); MMP10(+)^ 6^ ANGPTL4 (−), HMOX1 (+), IL-8 (+), MX1 (−), TXNIP (−), ITGB4 (−), ASAP3 (−), SPP1 (+), IFIT1(−), ZNF467 (−), IFI44L (−), PSAT1 (+), PTPRR (+); ^7^HMOX1 (+), MX1 (−), TXNIP(−), ITGB4 (−), CBS (+), GNG10 (+), STC1 (+), SLC2A1 (−), SLC3A2 (+), SEMA4B (−), PSAT1 (+); ^8^ LOX (+); + = * up-regulation; –  =  down-regulation.

### Quantitative real-time PCR of selected transcripts involved in the process of angiogenesis

In addition to genes showing outstanding degrees of alteration in expression, genes coding for proteins that had been recognized earlier to be involved in cell aggregation on the RPM [27], [28] were detected in the microarray analysis (Table S2). The list of these genes included *TUBB6, TGM2, SPATAN1, GSN, SERPINE1, ANXA2, CALD1, CAV1, SPAG9, PECAM1, ENG, IL-6* and *IL8.* We added the *ICAM1, ITGB1, MSN, RDX, TLN1, VIL2,* and *VIM* genes, although they did not emerge in the microarray, because their products had already attracted our attention earlier [26]. Then we tested the expression of all the genes mentioned above using quantitative real-time PCR (qPCR) (Table 3). We could not detect a significant transcriptional regulation for *ANXA2, CAV1, ENG, and SPTAN1*, although in most cases (with *ENG* as the most prominent example) their general expression patterns matched those observed in the microarray analysis (Table S2). The changes in expression determined by qPCR did not correlate to those from the microarray experiments for *TGM2* and *CALD1*; in these cases, qPCR indicated an increase in both AD and 3D samples after five days, whereas a decrease was observed in the microarray analysis (Table 3). A correlation between the microarray and qPCR experiments, however, was seen for *TUBB6, SPAG9, IL6*, and *IL8* after five days and for *SERPINE1, PECAM1, IL6,* and *IL8* after 7 days in AD and 3D cells. In addition, qPCR revealed significant up-regulation for *ITGB1, MSN, RDX,* and *VIL2* genes after 5 days.

**Table 3:** Comparison of gene array results and the relative quantities of mRNA determined by qPCR.

		*5d change*		*7d change*	
*Gene symbol*	*Protein name*	*3D vs. 1g FC Microarray/qPCR*	*AD vs. 1g FC Microarray/qPCR*	*3D vs. 1g FC Microarray/qPCR*	*AD vs. 1g FC Microarray/qPCR*
*TUBB6*	Tubulin beta-6 chain	2.02/162:100*	2.07/182:100*	1.12/135:100*	1.24/105:100
*TGM2*	Protein-glutamine-γ-glutamyltransferase	−1.50/210:100*	−1.56/147:100	1.31/92:100	1.23/104:100
*SPTAN1*	Spectrin alpha chain, brain	−2.21/95:100	−2.03/87:100	−1.43/72:100	−1.27/74:100
*GSN*	Gelsolin	−1.40/123:100	−1.45/111:100	−1.89/91:100	−2.36/54:100*
*SERPINE1*	Plasminogen activator**inhibitor 1	−1.47/63:100	1.08/96:100	1.52/245:100*	1.46/187:100*
*ANXA2*	Annexin A2	2.84/75:100	2.55/106:100	−1.09/117:100	1.48/116:100
*CALD1*	Caldesmon	−1.51/193:100*	−1.23/207:100*	1.46/124:100	−1.28/109:100
*CAV1*	Caveolin-1	−1.46/75:100	−1.19/93:100	−1.54/75:100	1.11/98:100
*SPAG9*	C-Jun-amino-terminal**kinase interacting protein 4	1.97/237:100*	1.93/169:100	1.04/134:100	1.23/100:100
*PECAM1*	Platelet endothelial cell adhesion mol.	−1.8/63:100	−1.7/65:100	−1.86/48:100*	−1.34/45:100*
*ENG*	Endoglin	−2.6/69:100	−2.2/64:100	−1.16/95:100	−1.06/107:100
*IL6*	Interleukin-6	2.37/346:100*	1.91/354:100*	2.32/728:100*	1.34/197:100
*IL8*	Interleukin-8	6.18/1734:100*	4.66/550:100*	5.4/1123:100*	3.36/564:100*
*ICAM1*	Intercellular adhesion**molecule 1	n.d./72:100	n.d./79:100	n.d./131:100	n.d./54:100*
*ITGB1*	Integrin beta-1	n.d./250:100*	n.d./207:100*	n.d./136:100	n.d./150:100
*MSN*	Moesin	n.d./175:100*	n.d./149:100	n.d./104:100	n.d./79:100
*RDX*	Radixin	n.d./197:100*	n.d./171:100	n.d./87:100	n.d./90:100
*TLN1*	Talin-1	n.d./157:100	n.d./193:100*	n.d./143:100	n.d./97:100
*VIL2*	Ezrin	n.d./180:100*	n.d./231:100*	n.d./117:100	n.d./127:100
*VIM*	Vimentin	n.d./150:100	n.d./183:100	n.d./129:100	n.d./128:100

* significant changes; n.d. not detected.

### Interactions of observed genes

**Figure 3:**
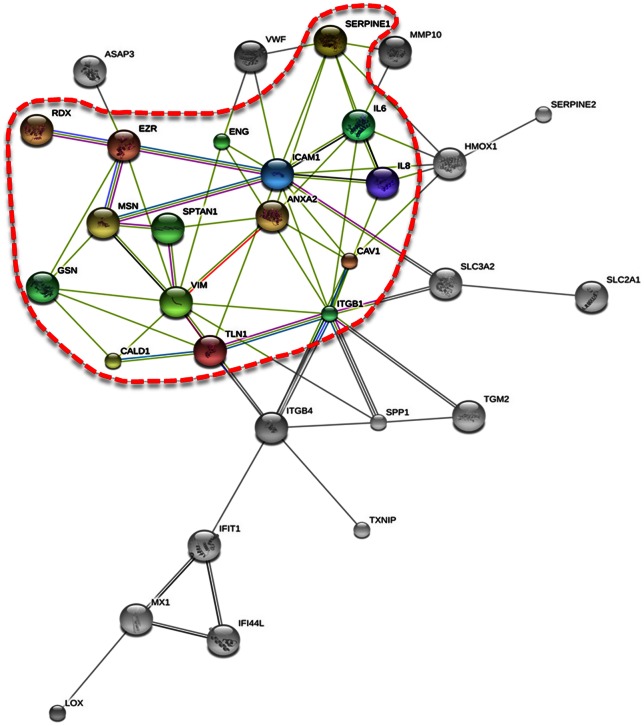
Interaction Analysis. STRING visualization of interactions between the proteins of genes playing a role in 3D aggregation and shown in Table 3 (surrounded by a red line) and genes whose level of interaction was changed at least 4-fold (Table 2). IL-8 belongs to both groups.

After a large number of genes were determined to be up- or downregulated when ECs were incubated on the RPM, it was of interest to see whether there are interactions between them. Application of STRING analysis revealed that the corresponding proteins of genes playing a role in 3D aggregation and shown in Table 3 form an interacting network, with the exception of TUBB6 and SPAG9 (Fig. 3, surrounded by a red line). Proteins of genes whose level of interaction changed at least 4-fold (Table 2) did not show significant interaction by themselves. Combining both groups, however, revealed that 15 of the 27 genes indicated in Table 2 clearly fitted into and extended the network formed by the earlier group indicated in Table 3 (Fig. 3, whole graph). These results suggest that an entire system of genes whose products are involved in maintaining cell structure and contact is changed when ECs start to form tubes on the RPM.

## Discussion

Looking for genes involved in the formation of 3D aggregates formed by EA.hy926 endothelial cells (Fig. 1), the genomic approach described in this paper was applied to search for differentially expressed transcripts in cells cultured on the RPM as compared to those incubated in a normal laboratory incubator (1 *g*). In accordance with the microscopic observation that oblong 3D aggregates, which we consider as precursors of the single-layered tubular structures observed after two weeks of incubation on the RPM [12], appear in cell cultures incubated on the RPM for 5 days (Fig. 1), we found a strong overrepresentation of transient expression differences in the five-day, RPM-treated samples. At that time, when the early stages of tubular structures appear, some clusters of genes were down-regulated, while others were up-regulated (Fig. 2B). However, amongst the 1,625 genes affected by annulling gravity, there were only 27 genes whose level of expression was up- or down-regulated at least 4-fold (Table 2). Fifteen of them, including *IL8* and *VWF* as the most prominently affected, showed linkages to genes known to be involved in angiogenesis (Fig. 3), while two other genes are important for serine biosynthesis (Table 2).

Evaluating the results of our experiments, we focused on two groups of genes: those whose expression was dramatically changed (>4-fold, Table 2) and those that are known to be involved in angiogenesis but were up- or down-regulated less than 4-fold (Table 3). In comparison to EC growing under normal 1 *g* conditions, the *VWF* gene was 6-fold down-regulated in 3D aggregates after 7 days of culturing on the RPM. Von Willebrand factor is a protein important in hemostasis and angiogenesis [29]. Its down-regulation during formation of vascular-like networks by HUVEC cells encapsulated in hydrogel was already reported [30]. A 6-fold up-regulation was observed for the *IL8* gene, while the *IL6* gene was 2–3 fold up-regulated (Tables 2, 3). IL-8 enhances endothelial cell proliferation, induces capillary tube organization and up-regulates the expression of anti-apoptotic genes [31]. IL-6 also induces proliferation, migration and tube formation in various types of endothelial cells [32], [33]. In our experiments, up-regulation of the IL6 and IL8 genes was most prominent in 3D aggregates after 5 days of culturing on the RPM (Table 3). In human ovarian cancer cells, *IL8* is up-regulated together with *IL6* by the nuclear factor-kappa B (NFκB) pathway [34]. Thus, our findings further support the idea that NFκB may play a special role when human cells start to form 3D aggregates under simulated microgravity [35].

According to our STRING analysis, IL-6 and IL-8 interact together with the HMOX1, ICAM1 and ITGB1 proteins, which are nodal points in a network of interaction of the detected genes (Fig. 3). The *HMOX1* gene was up-regulated more than 4-fold after 5 days of incubation on the RPM and less than 2-fold after 7 days (Table S2). It encodes heme-oxygenase-1, which facilitates angiogenesis [36]. The intercellular adhesion molecule-1 (ICAM-1) is an endothelial-associated transmembrane protein. It is important in stabilizing cell-cell interactions and facilitating leukocyte endothelial transmigration [37]. In accordance with a previous study [12], we did not see significant regulation of the *ICAM*-*1* gene. ICAM-1 has been shown to interact with ezrin and moesin [38], whose genes (*VIL2*, *MSN*) were significantly upregulated in AD cells and 3D aggregates after 5 days. Ezrin and moesin belong to the ERM protein family, which also includes radixin. These proteins have the ability to interact with both the plasma membrane and filamentous actin [39]. Moesin is required in ECs for *in vivo* tubulogenesis [40]. Its regulation resembles *TUBB6* gene regulation (Table 3). MSN extends the network of interaction via GSN, VIM and SPTAN1 (Fig. 3). *GSN* codes for the actin-binding protein gelsolin, which has multiple cellular functions including apoptosis and is reported to be down-regulated in various cancers and premalignant lesions [41]. The *SPTAN1* gene encodes another cytoskeletal protein known as spectrin, the non-erythroid alpha chain [42]. Both genes showed a tendency of being down-regulated when the ECs were incubated on the RPM, while *VIM* gene expression was not significantly changed on the RPM (Table 3).

When we investigated the *ITGB1* gene by qPCR, significant upregulation was observed, although it was not detected in the gene array analysis (Table 3). The increase in mRNA levels was substantial after five days, but only slightly elevated after seven days of culturing on the RPM (Table 3). *In vivo,* the cytoplasmic tail of the β-integrin subunit is coupled to F-actin by talin. This link is required to transmit force from the actin cytoskeleton to the extracellular matrix [43]. ECs lacking talin 1 *in vivo* are unable to undergo the cell spreading and flattening required for vessel formation [44]. We found that *TLN1* gene expression was significantly elevated on day five in AD cells of RPM cultures, but not on day seven (Table 3). At the same time, *SPP1*, another interaction partner of *ITGB1*, was 4-fold up-regulated, while *ITGB4* was 4-fold down-regulated as measured in the gene array analysis. Integrin beta 4 chains are involved in neoangiogenesis just like beta 1 chains. *SPP1* codes for osteopontin, a ligand of integrins [45]. Enhancement of cellular osteopontin has been observed in ECs *in vitro* when gravity was reduced during incubation [10] and *in vivo* during vascular remodelling [46]. These findings suggest that IL-8 and IL-6 may trigger a cascade of reactions of cytoskeleton related genes and proteins in the early phases of tube formation.

According to our STRING analysis, the cascade of possible mutual interactions that leads to tube formation includes further nodes (Fig. 3). Endoglin (ENG) is found on the surface of cells and is important for angiogenesis, as it regulates endothelial signaling and function during blood vessel development [47], [48]. Caveolin-1 (CAV1*)* supports the assembly of caveolae, which are the sites in the cell membrane responsible for concentrating an array of signaling molecules critical for endothelial cell function [49], [50]. Serpine peptidase inhibitor-1 (SERPINE1) interacts with extracellular matrix proteins as well as with transmembrane receptors and other links to the intracellular signaling machinery. Thus, it modulates cell migration, cell-matrix interactions, signaling pathways and angiogenesis [51]. We detected a non-significant down-regulation of the *ENG* gene on day five, whereas on day seven there was no change. Similarly, *CAV1* remained unchanged on days five and seven in AD and exhibited a moderate down-regulation in 3D aggregates. As caveolin-1 is thought to play a role as a negative regulator of signal transduction [52], downregulation of *CAV1* may indicate a contribution to tube formation. *SERPINE1* (*PAI-1*) gene expression was significantly up-regulated on day seven in AD and 3D cultures, while on day five it was significantly down-regulated in 3D cultures and unaltered in AD. Earlier measurements of this factor's presence in culture supernatants indicated that less PAI-1 is secreted in the supernatants of RPM cultures than in control 1 *g* cultures [18]. The difference may be explained by a strong binding of PAI-1 to the cells on the RPM. PAI-1 promotes tumour angiogenesis by preventing excessive proteolysis [53]. Thus, its association with cells may better protect them against plasmin activity, which is promoted by Annexin A2 (ANXA2*),* whose gene expression remained unchanged in all groups. Considering that proteolytic digestion of spectrin [54] favours angiogenesis, while plasmin activity is reduced [53], the present results support the idea of a complicated regulation of proteolysis during the initial phase of tube formation, as already suspected in a previous publication [11].

A prominent role for balanced proteolysis in EC tube formation also became obvious when gene profiles of AD and 3D cells harvested after 7 days of incubation on the RPM were compared. At that time, when the initial phase of cell aggregation is over, 10% of the genes (i.e. *MMP2*, *MMP9*, *MMP10)*, whose expression differed at least 2-fold between AD and 3D cells encode metalloproteinases, which are involved in remodeling the extracellular matrix of blood vessels [55]. In addition, the *NFKBZI* and *NR4A2* genes were up-regulated 3-fold in 3D as compared to AD cells after 7 days of incubation on the RPM. The expression of these genes is important for maintaining IL-6 and IL-8 production, respectively [56], [57]. Hence, it appears reasonable that these interleukins are also important in the elongation of formed tubes.

Taken together, the genomic approach applied in this study proved that a network of genes responsible for maintaining cellular structures and contacts is modulated when endothelial cells transit from a two- to a three-dimensional type of growth. Similar results were obtained when we studied spheroid formation of thyroid cancer cells [26], [28]. But investigating the thyroid cells, we noticed several enzymes of glycolysis that were modulated when gravity was reduced during cell culturing [58]. In this study, the *PHGDH* and *PSATI* genes were noted to be upregulated 5- and 4-fold, respectively. These genes encode for phosphoglycerate dehydrogenase and phosphoserine aminotransferase, which are involved in metabolizing pyruvate to L-serine. To our best knowledge, a role of these enzymes in *in vitro* tube formation has not been described so far. But they are critical for neurogenesis [59] and enhance the aggressiveness of various types of cancer cells [60], [61].

## Supporting Information

Table S1 **Primers used for quantitative real-time PCR.**
(DOCX)

Table S2 **Biological functions retrievable in the gene ontology database (GO) could be assigned to 1174 of the 1625 differentially expressed transcripts.**
(DOCX)
