# Supplementary material for: Genomic Approach to Identify Factors That Drive the Formation of Three-Dimensional Structures by EA.hy926 Endothelial Cells
Source: PLoS One. 2013 May 10;8(5):e64402. doi: 10.1371/journal.pone.0064402 (PMC3651237; doi:10.1371/journal.pone.0064402)
Supplement: Table S1 — Primers used for quantitative real-time PCR. (DOCX) [file pone.0064402.s001.docx]

**Supplementary Table S1: Primers used for quantitative real-time PCR**

| ***Gene*** | ***Primer Name*** | ***Sequence*** |
| --- | --- | --- |
| *18S rRNA* | 18S-F | GGAGCCTGCGGCTTAATTT |
|  | 18S-R | CAACTAAGAACGGCCATGCA |
| *ANXA2* | ANXA2-F | GGTACAAGAGTTACAGCCCTTATGACA |
|  | ANXA2-R | CATGGAGTCATACAGCCGATCA |
| *CALD1* | CALD1-F | CCGCATCAATGAATGGCTAACT |
|  | CALD1-R | GGGAAGTGACCTTATCCACAGATT |
| *CAV1* | CAV1-F | GTACGACGCGCACACCAA |
|  | CAV1-R | TCCCTTCTGGTTCTGCAATCA |
| *ENG* | ENG-F | TTGTCTTGCGCAGTGCTTACTC |
|  | ENG-R | CCTTTTTCCGCTGTGGTGAT |
| *GSN* | GSN-F | GCCACCTTCCTAGGCTACTTCA |
|  | GSN-R | CTCTGCACCACCACCTCGTT |
| *ICAM1* | ICAM1-F | CGGCTGACGTGTGCAGTAAT |
|  | ICAM1-R | CTTCTGAGACCTCTGGCTTCGT |
| *IL6* | IL6-F | CGGGAACGAAAGAGAAGCTCTA |
|  | IL6-R | GAGCAGCCCCAGGGAGAA |
| *IL8* | IL8-F | TGGCAGCCTTCCTGATTTCT |
|  | IL8-R | GGGTGGAAAGGTTTGGAGTATG |
| *ITGB1* | ITGB1-F | GAAAACAGCGCATATCTGGAAATT |
|  | ITGB1-R | CAGCCAATCAGTGATCCACAA |
| *LAM* | LAM-F | TGCTCATGGTCAATGCTAATCTG |
|  | LAM-R | TCTATCAATCCTCTTCCTTGGACAA |
| *MSN* | MSN-F | GAAATTTGTCATCAAGCCCATTG |
|  | MSN-R | CCATGCACAAGGCCAAGAT |
| *PAI1* | PAI1-F | AGGCTGACTTCACGAGTCTTTCA |
|  | PAI1-R | CACTCTCGTTCACCTCGATCTTC |
| *PECAM1* | PECAM1-F | GGATCCATATGCAGACCTCAGAA |
|  | PECAM1-F | CTGATGTGGAACTTGGGTGTAGAG |
| *RDX* | RDX-F | GAAAATGCCGAAACCAATCAA |
|  | RDX-R | GTATTGGGCTGAATGGCAAATT |
| *SPAG9* | SPAG9-F | ATTTCAGGAATTAAGTCAACCACGTT |
|  | SPAG9-R | GTTGATGCTGGAGTGGTAGCTTTAG |
| *SPTAN1* | SPTAN-F | GACACGGTGGATCCGAACAG |
|  | SPTAN-R | CGCTGGACTTGACGTTCTCA |
| *TGM2* | TGM2-F | AAGAGGAGCGGCAGGAGTATG |
|  | TGM2-R | GCCCAAAATTCCAAGGTATGTTC |
| *TLN1* | TLN1-F | GATGGCTATTACTCAGTACAGACAACTGA |
|  | TLN1-R | CATAGTAGACTCCTCATCTCCTTCCA |
| *TUBB6* | TUBB6-F | GTGCGGTCTGGGCCTTTT |
|  | TUBB6-R | CTCCGTGTAGTGCCCTTTCG |
| *VIL2* | VIL2-F | GAAGTGCACAAGTCTGGGTACCT |
|  | VIL2-R | CTCCCACTGGTCCCTGGTAAG |
| *VIM* | VIM-F | TTCAGAGAGAGGAAGCCGAAAAC |
|  | VIM-R | AGATTCCACTTTGCGTTCAAGGT |
|  |  |  |
|  |  |  |

All sequences are given in 5’-3’ direction
