# Supplementary material for: Genomic Approach to Identify Factors That Drive the Formation of Three-Dimensional Structures by EA.hy926 Endothelial Cells
Source: PLoS One. 2013 May 10;8(5):e64402. doi: 10.1371/journal.pone.0064402 (PMC3651237; doi:10.1371/journal.pone.0064402)
Supplement: Table S2 — Biological functions retrievable in the gene ontology database (GO) could be assigned to 1174 of the 1625 differentially expressed transcripts. (DOCX) [file pone.0064402.s002.docx]

**Supplementary Table S2: Biological functions retrievable in the gene ontology database (GO) could be assigned to 1174 of the 1625 differentially expressed transcripts.**

|  |  | ***5d*** | | | | | | ***7d*** | | | | | |
| --- | --- | --- | --- | --- | --- | --- | --- | --- | --- | --- | --- | --- | --- |
| ***GO Biological Process*** | ***Gene symbol*** | ***FC  AD vs. 1g*** | ***p-value*** | ***FC  3D vs. 1g*** | ***p-value*** | ***FC 3D vs. AD*** | ***p-value*** | ***FC AD vs. 1g*** | ***p-value*** | ***FC 3D vs. 1g*** | ***p-value*** | ***FC 3D vs. AD*** | ***p-value*** |
| **Angiogenesis** | *ADORA2A* | -3.04 | 4.01E-06 | -2.67 | 1.86E-05 | 1.14 | 4.60E-01 | -1.85 | 1.98E-03 | -2.15 | 2.82E-04 | -1.16 | 3.93E-01 |
|  | *ANG* | -1.41 | 5.07E-04 | -1.25 | 1.27E-02 | 1.13 | 1.61E-01 | 1.39 | 6.85E-04 | 1.41 | 5.08E-04 | 1.01 | 8.95E-01 |
|  | *ANGPTL4* | -4.80 | 5.87E-09 | -5.60 | 1.33E-09 | -1.17 | 3.23E-01 | -1.11 | 5.18E-01 | -1.24 | 1.69E-01 | -1.13 | 4.49E-01 |
|  | *ANXA2* | 2.55 | 3.21E-04 | 2.84 | 1.05E-04 | 1.11 | 6.17E-01 | 1.48 | 8.06E-02 | -1.09 | 6.83E-01 | -1.61 | 3.60E-02 |
|  | *B4GALT1* | -1.51 | 7.33E-05 | -1.96 | 1.36E-07 | -1.30 | 4.71E-03 | -1.32 | 2.82E-03 | 1.13 | 1.53E-01 | 1.49 | 1.04E-04 |
|  | *BGN* | -3.42 | 4.81E-10 | -3.74 | 1.52E-10 | -1.09 | 3.88E-01 | 1.95 | 3.83E-06 | -1.37 | 6.49E-03 | -2.67 | 1.63E-08 |
|  | *CAV1* | -1.19 | 2.86E-01 | -1.46 | 2.87E-02 | -1.23 | 2.18E-01 | 1.11 | 5.22E-01 | -1.54 | 1.52E-02 | -1.70 | 3.68E-03 |
|  | *CAV2* | -1.11 | 4.57E-01 | -1.46 | 1.60E-02 | -1.31 | 7.37E-02 | 1.35 | 4.78E-02 | 1.09 | 5.59E-01 | -1.24 | 1.44E-01 |
|  | *CDH5* | -2.21 | 2.06E-04 | -3.21 | 2.27E-06 | -1.45 | 4.38E-02 | -1.39 | 7.02E-02 | -1.84 | 2.30E-03 | -1.32 | 1.22E-01 |
|  | *CEACAM1* | 1.13 | 2.26E-01 | 1.27 | 2.07E-02 | 1.13 | 2.16E-01 | -1.72 | 1.95E-05 | -2.10 | 2.99E-07 | -1.23 | 4.49E-02 |
|  | *CITED2* | 1.72 | 5.97E-04 | 1.97 | 6.40E-05 | 1.14 | 3.22E-01 | 1.51 | 5.60E-03 | 1.32 | 5.00E-02 | -1.15 | 3.10E-01 |
|  | *COL18A1* | -2.44 | 2.70E-07 | -2.32 | 6.08E-07 | 1.05 | 6.60E-01 | -1.59 | 6.14E-04 | -1.46 | 3.23E-03 | 1.09 | 4.65E-01 |
|  | *COL4A2* | -1.75 | 1.46E-05 | -2.32 | 6.12E-08 | -1.32 | 8.97E-03 | -1.23 | 4.63E-02 | -1.20 | 7.44E-02 | 1.02 | 8.08E-01 |
|  | *COL4A5* | -1.61 | 1.41E-05 | -1.82 | 7.44E-07 | -1.13 | 1.51E-01 | -1.02 | 7.67E-01 | -1.15 | 9.50E-02 | -1.13 | 1.61E-01 |
|  | *CTGF* | -1.01 | 9.48E-01 | -2.14 | 1.58E-06 | -2.13 | 1.79E-06 | 1.23 | 7.64E-02 | -1.21 | 9.75E-02 | -1.48 | 1.92E-03 |
|  | *CYR61* | -1.12 | 4.38E-01 | -1.84 | 3.68E-04 | -1.64 | 2.15E-03 | -1.16 | 3.10E-01 | -1.31 | 6.92E-02 | -1.13 | 3.86E-01 |
|  | *DHCR7* | 1.62 | 6.72E-03 | -1.11 | 5.23E-01 | -1.80 | 1.59E-03 | 1.11 | 5.23E-01 | -1.34 | 8.14E-02 | -1.49 | 2.25E-02 |
|  | *EGFL7* | -1.76 | 3.20E-04 | -2.00 | 3.72E-05 | -1.14 | 3.34E-01 | -1.51 | 4.51E-03 | -1.40 | 1.77E-02 | 1.08 | 5.34E-01 |
|  | *ENG* | -2.20 | 1.07E-05 | -2.61 | 8.59E-07 | -1.18 | 2.14E-01 | -1.06 | 6.44E-01 | -1.16 | 2.86E-01 | -1.09 | 5.36E-01 |
|  | *EPAS1* | 2.33 | 1.13E-04 | 2.45 | 6.04E-05 | 1.05 | 7.74E-01 | 1.10 | 6.01E-01 | -1.39 | 7.20E-02 | -1.52 | 2.50E-02 |
|  | *EPHB4* | -2.25 | 1.51E-05 | -2.21 | 1.95E-05 | 1.02 | 9.03E-01 | -1.38 | 3.25E-02 | -1.85 | 2.99E-04 | -1.35 | 4.56E-02 |
|  | *GCLM* | 2.94 | 1.96E-04 | 2.92 | 2.11E-04 | -1.01 | 9.74E-01 | -1.00 | 9.97E-01 | -1.50 | 9.47E-02 | -1.50 | 9.54E-02 |
|  | *GPI* | -1.87 | 8.24E-06 | -2.29 | 1.88E-07 | -1.23 | 6.13E-02 | 1.34 | 9.81E-03 | 1.14 | 2.21E-01 | -1.18 | 1.23E-01 |
|  | *HIF1A* | 2.26 | 2.17E-05 | 2.97 | 5.13E-07 | 1.32 | 7.23E-02 | 1.06 | 6.78E-01 | -1.13 | 4.01E-01 | -1.20 | 2.16E-01 |
|  | *HMOX1* | 4.14 | 1.21E-09 | 4.58 | 4.03E-10 | 1.11 | 4.30E-01 | 1.55 | 2.40E-03 | 1.94 | 4.68E-05 | 1.25 | 9.02E-02 |
|  | *IL8* | 4.66 | 1.44E-04 | 6.18 | 2.18E-05 | 1.33 | 3.90E-01 | 3.36 | 1.36E-03 | 5.40 | 5.26E-05 | 1.61 | 1.56E-01 |
|  | *JUNB* | -1.47 | 1.58E-03 | -1.21 | 8.31E-02 | 1.21 | 7.63E-02 | -1.08 | 4.54E-01 | 1.72 | 5.57E-05 | 1.86 | 1.12E-05 |
|  | *KCNMA1* | -1.05 | 7.02E-01 | -1.11 | 3.83E-01 | -1.06 | 6.20E-01 | 1.31 | 3.52E-02 | 1.78 | 1.26E-04 | 1.36 | 1.88E-02 |
|  | *KLK3* | 1.03 | 8.82E-01 | 1.16 | 4.80E-01 | 1.13 | 5.75E-01 | 1.81 | 1.13E-02 | 1.17 | 4.64E-01 | -1.55 | 5.28E-02 |
|  | *LEPR* | -2.37 | 8.12E-06 | -2.45 | 4.98E-06 | -1.03 | 8.10E-01 | 1.55 | 5.87E-03 | 1.74 | 9.14E-04 | 1.12 | 4.13E-01 |
|  | *LOX* | 1.02 | 9.14E-01 | -1.05 | 7.10E-01 | -1.07 | 6.32E-01 | 4.11 | 6.06E-09 | 3.44 | 4.66E-08 | -1.19 | 2.14E-01 |
|  | *MAP3K7* | 1.54 | 1.17E-03 | 1.92 | 1.59E-05 | 1.25 | 6.30E-02 | 1.14 | 2.67E-01 | -1.11 | 3.61E-01 | -1.26 | 5.18E-02 |
|  | *MMP2* | -1.60 | 9.08E-07 | -2.01 | 2.68E-09 | -1.26 | 2.44E-03 | 1.58 | 1.22E-06 | -1.45 | 2.06E-05 | -2.29 | 1.69E-10 |
|  | *NOTCH1* | -2.41 | 5.66E-07 | -1.93 | 2.31E-05 | 1.25 | 7.57E-02 | -1.38 | 1.37E-02 | -1.67 | 3.63E-04 | -1.21 | 1.17E-01 |
|  | *PDE5A* | -1.50 | 1.01E-02 | -1.70 | 1.56E-03 | -1.13 | 4.07E-01 | -1.04 | 8.09E-01 | 1.03 | 8.38E-01 | 1.07 | 6.56E-01 |
|  | *PDGFB* | -2.23 | 1.35E-07 | -2.72 | 4.78E-09 | -1.22 | 5.30E-02 | 1.12 | 2.54E-01 | 1.01 | 9.01E-01 | -1.11 | 3.06E-01 |
|  | *PLAT* | -1.38 | 1.27E-02 | -1.68 | 3.12E-04 | -1.22 | 1.10E-01 | -1.19 | 1.58E-01 | 1.26 | 6.55E-02 | 1.49 | 2.95E-03 |
|  | *PLAU* | 1.28 | 4.13E-03 | 1.21 | 2.17E-02 | -1.06 | 4.51E-01 | 2.21 | 3.20E-09 | 2.47 | 3.96E-10 | 1.12 | 1.51E-01 |
|  | *PLCD3* | -1.69 | 4.24E-05 | -1.94 | 2.25E-06 | -1.15 | 1.66E-01 | 1.05 | 5.94E-01 | 1.02 | 8.24E-01 | -1.03 | 7.55E-01 |
|  | *PNPLA6* | -2.08 | 4.52E-05 | -1.95 | 1.24E-04 | 1.07 | 6.44E-01 | -1.15 | 3.31E-01 | -1.23 | 1.51E-01 | -1.07 | 6.23E-01 |
|  | *PPAP2B* | 2.02 | 1.30E-04 | 2.06 | 9.86E-05 | 1.02 | 8.99E-01 | 1.64 | 3.22E-03 | 1.48 | 1.54E-02 | -1.11 | 4.81E-01 |
|  | *RECK* | -2.18 | 8.78E-06 | -2.40 | 1.86E-06 | -1.10 | 4.42E-01 | 1.23 | 1.15E-01 | -1.33 | 3.87E-02 | -1.64 | 1.08E-03 |
|  | *ROBO4* | -1.94 | 1.18E-04 | -2.73 | 6.93E-07 | -1.41 | 2.06E-02 | -1.58 | 3.32E-03 | -1.50 | 7.67E-03 | 1.05 | 7.08E-01 |
|  | *SEMA3C* | -1.31 | 2.90E-01 | -1.89 | 2.04E-02 | -1.44 | 1.63E-01 | 1.70 | 4.78E-02 | 1.12 | 6.56E-01 | -1.52 | 1.12E-01 |
|  | *SERPINE1* | 1.08 | 5.30E-01 | -1.47 | 6.23E-03 | -1.59 | 1.51E-03 | 1.46 | 7.13E-03 | 1.52 | 3.59E-03 | 1.04 | 7.59E-01 |
|  | *SHB* | -1.86 | 1.18E-04 | -1.66 | 9.24E-04 | 1.13 | 3.64E-01 | -1.04 | 7.68E-01 | 1.01 | 9.14E-01 | 1.05 | 6.87E-01 |
|  | *SOD2* | 1.84 | 3.56E-05 | 1.97 | 1.06E-05 | 1.07 | 5.67E-01 | 1.38 | 1.01E-02 | 1.27 | 4.46E-02 | -1.08 | 4.83E-01 |
|  | *SOX17* | 1.83 | 8.60E-06 | 2.22 | 1.93E-07 | 1.22 | 6.06E-02 | -1.14 | 2.05E-01 | -1.00 | 9.70E-01 | 1.13 | 2.18E-01 |
|  | *SOX18* | -1.99 | 2.52E-04 | -2.17 | 7.54E-05 | -1.09 | 5.87E-01 | -1.31 | 8.86E-02 | -2.70 | 3.77E-06 | -2.05 | 1.62E-04 |
|  | *TBX1* | -1.89 | 1.41E-06 | -1.89 | 1.48E-06 | 1.00 | 9.82E-01 | 1.21 | 4.85E-02 | -1.43 | 9.97E-04 | -1.73 | 1.04E-05 |
|  | *TGM2* | -1.56 | 4.06E-03 | -1.50 | 7.39E-03 | 1.04 | 7.88E-01 | 1.23 | 1.41E-01 | 1.31 | 6.30E-02 | 1.06 | 6.64E-01 |
|  | *THY1* | -2.26 | 3.82E-07 | -2.08 | 1.70E-06 | 1.09 | 4.34E-01 | -1.09 | 4.13E-01 | 1.06 | 5.66E-01 | 1.16 | 1.72E-01 |
|  | *VASH1* | -1.41 | 1.34E-02 | -1.58 | 1.82E-03 | -1.12 | 3.73E-01 | -1.60 | 1.43E-03 | -1.72 | 3.98E-04 | -1.07 | 5.73E-01 |
|  |  |  |  |  |  |  |  |  |  |  |  |  |  |
| **Apoptosis** | *ADAM9* | -1.20 | 1.46E-01 | -1.02 | 8.60E-01 | 1.18 | 1.96E-01 | -1.07 | 5.70E-01 | 1.42 | 9.68E-03 | 1.52 | 2.72E-03 |
|  | *ADAMTSL4* | -1.60 | 6.55E-05 | -1.60 | 7.31E-05 | 1.00 | 9.60E-01 | 1.17 | 1.06E-01 | 1.25 | 2.40E-02 | 1.07 | 4.56E-01 |
|  | *ADM* | -2.65 | 3.52E-11 | -2.09 | 3.30E-09 | 1.27 | 2.79E-03 | 1.14 | 7.57E-02 | 1.14 | 7.83E-02 | -1.00 | 9.86E-01 |
|  | *AHR* | -2.47 | 1.87E-04 | -2.04 | 1.67E-03 | 1.21 | 3.37E-01 | -1.37 | 1.22E-01 | 1.25 | 2.61E-01 | 1.71 | 1.23E-02 |
|  | *ALDOC* | -2.17 | 1.11E-05 | -2.32 | 3.94E-06 | -1.07 | 6.14E-01 | 2.09 | 2.07E-05 | 1.30 | 5.51E-02 | -1.60 | 1.81E-03 |
|  | *APLP1* | -2.50 | 1.11E-09 | -3.34 | 1.27E-11 | -1.33 | 2.09E-03 | 1.13 | 1.44E-01 | 1.32 | 2.85E-03 | 1.17 | 7.04E-02 |
|  | *ASNS* | 3.79 | 1.34E-08 | 3.94 | 8.72E-09 | 1.04 | 7.87E-01 | 1.82 | 3.78E-04 | 2.32 | 8.06E-06 | 1.28 | 8.78E-02 |
|  | *BCAR1* | -2.85 | 1.88E-08 | -3.04 | 7.52E-09 | -1.07 | 5.63E-01 | 1.13 | 2.82E-01 | 1.20 | 1.09E-01 | 1.07 | 5.70E-01 |
|  | *BCL2L1* | 2.74 | 1.41E-05 | 2.88 | 7.56E-06 | 1.05 | 7.63E-01 | 1.14 | 4.61E-01 | 1.25 | 2.07E-01 | 1.10 | 5.86E-01 |
|  | *BCLAF1* | 1.85 | 3.01E-04 | 1.86 | 2.60E-04 | 1.01 | 9.48E-01 | -1.07 | 6.50E-01 | -1.43 | 1.77E-02 | -1.34 | 4.55E-02 |
|  | *BIRC3* | -1.66 | 8.46E-02 | -1.83 | 4.32E-02 | -1.10 | 7.31E-01 | -1.49 | 1.67E-01 | 1.10 | 7.32E-01 | 1.65 | 9.07E-02 |
|  | *BNIP3* | -2.08 | 7.58E-09 | -1.96 | 2.75E-08 | 1.06 | 4.24E-01 | 1.18 | 3.92E-02 | 1.32 | 1.19E-03 | 1.12 | 1.23E-01 |
|  | *BNIP3L* | -1.53 | 9.82E-02 | -1.69 | 4.48E-02 | -1.11 | 6.85E-01 | 1.34 | 2.47E-01 | 1.42 | 1.64E-01 | 1.06 | 8.02E-01 |
|  | *C11orf82* | 1.26 | 6.66E-02 | -1.07 | 5.99E-01 | -1.34 | 2.29E-02 | -1.08 | 5.02E-01 | -1.55 | 1.60E-03 | -1.43 | 7.27E-03 |
|  | *C3orf38* | 1.73 | 1.90E-03 | 2.05 | 1.56E-04 | 1.19 | 2.74E-01 | 1.16 | 3.46E-01 | -1.15 | 3.60E-01 | -1.33 | 7.27E-02 |
|  | *CARD10* | -2.03 | 1.37E-07 | -1.93 | 4.01E-07 | 1.05 | 5.54E-01 | -1.37 | 1.66E-03 | -1.66 | 1.17E-05 | -1.21 | 3.46E-02 |
|  | *CDKN1A* | 1.78 | 1.38E-04 | 1.88 | 4.80E-05 | 1.06 | 6.30E-01 | 1.06 | 6.34E-01 | 1.68 | 3.86E-04 | 1.59 | 1.13E-03 |
|  | *CEBPB* | 2.53 | 1.81E-06 | 2.39 | 4.04E-06 | -1.06 | 6.86E-01 | 1.38 | 2.77E-02 | 1.71 | 7.99E-04 | 1.24 | 1.21E-01 |
|  | *CFLAR* | -2.91 | 5.47E-05 | -2.73 | 1.07E-04 | 1.07 | 7.58E-01 | -1.69 | 1.86E-02 | -1.01 | 9.66E-01 | 1.68 | 2.04E-02 |
|  | *CRADD* | 1.90 | 1.07E-05 | 1.97 | 5.53E-06 | 1.04 | 7.48E-01 | 1.21 | 9.39E-02 | -1.06 | 5.98E-01 | -1.28 | 3.32E-02 |
|  | *DAP* | 1.22 | 1.59E-01 | 1.40 | 2.05E-02 | 1.15 | 2.97E-01 | -1.33 | 4.69E-02 | -1.17 | 2.42E-01 | 1.13 | 3.68E-01 |
|  | *DDIT3* | 3.31 | 6.10E-07 | 3.55 | 2.74E-07 | 1.07 | 6.64E-01 | 1.50 | 2.12E-02 | 1.96 | 5.16E-04 | 1.31 | 1.07E-01 |
|  | *DDIT4* | -2.24 | 1.01E-09 | -1.39 | 1.63E-04 | 1.60 | 2.50E-06 | 1.11 | 1.38E-01 | 1.03 | 7.09E-01 | -1.09 | 2.57E-01 |
|  | *DDX19B* | 1.99 | 7.64E-05 | 2.19 | 1.66E-05 | 1.10 | 4.79E-01 | 1.10 | 5.00E-01 | -1.06 | 6.92E-01 | -1.16 | 2.89E-01 |
|  | *DHCR24* | 1.74 | 3.92E-03 | -1.01 | 9.68E-01 | -1.75 | 3.58E-03 | -1.09 | 6.23E-01 | -1.91 | 1.15E-03 | -1.76 | 3.51E-03 |
|  | *DIDO1* | -1.57 | 1.54E-03 | -1.34 | 2.55E-02 | 1.17 | 2.13E-01 | -1.24 | 8.91E-02 | -1.42 | 9.77E-03 | -1.14 | 2.89E-01 |
|  | *DLC1* | -1.32 | 3.09E-01 | -1.64 | 8.07E-02 | -1.24 | 4.32E-01 | -1.30 | 3.48E-01 | 1.07 | 8.03E-01 | 1.39 | 2.39E-01 |
|  | *DOCK1* | -1.29 | 6.59E-02 | -1.50 | 6.25E-03 | -1.16 | 2.71E-01 | 1.25 | 1.06E-01 | 1.04 | 7.41E-01 | -1.19 | 1.90E-01 |
|  | *EEF1A2* | -1.63 | 2.48E-04 | -1.39 | 7.39E-03 | 1.18 | 1.43E-01 | 1.44 | 3.36E-03 | 2.14 | 1.47E-06 | 1.48 | 1.83E-03 |
|  | *EEF1E1* | 1.76 | 8.64E-04 | 2.10 | 6.06E-05 | 1.19 | 2.42E-01 | 1.21 | 1.91E-01 | 1.09 | 5.59E-01 | -1.11 | 4.55E-01 |
|  | *EI24* | 1.95 | 7.94E-04 | 1.64 | 8.13E-03 | -1.19 | 3.07E-01 | 1.11 | 5.34E-01 | -1.17 | 3.55E-01 | -1.30 | 1.30E-01 |
|  | *ETS1* | -1.10 | 6.72E-01 | -1.70 | 2.76E-02 | -1.55 | 6.48E-02 | 1.03 | 8.91E-01 | 1.21 | 3.96E-01 | 1.18 | 4.74E-01 |
|  | *FAF1* | -1.49 | 1.22E-04 | -1.71 | 3.47E-06 | -1.15 | 1.04E-01 | 1.10 | 2.49E-01 | 1.47 | 1.89E-04 | 1.33 | 2.66E-03 |
|  | *FNTA* | 1.58 | 2.86E-03 | 1.93 | 1.07E-04 | 1.22 | 1.55E-01 | 1.31 | 5.63E-02 | -1.00 | 9.77E-01 | -1.32 | 5.32E-02 |
|  | *FOSL1* | 1.17 | 1.44E-01 | 1.01 | 9.03E-01 | -1.16 | 1.78E-01 | -1.02 | 8.55E-01 | 2.37 | 1.86E-07 | 2.41 | 1.35E-07 |
|  | *GADD45A* | 2.43 | 8.68E-07 | 2.41 | 9.87E-07 | -1.01 | 9.46E-01 | 1.90 | 5.30E-05 | 2.19 | 4.52E-06 | 1.16 | 2.49E-01 |
|  | *GADD45B* | 1.15 | 2.20E-01 | 1.19 | 1.26E-01 | 1.04 | 7.44E-01 | 1.83 | 2.76E-05 | 2.48 | 1.32E-07 | 1.35 | 1.23E-02 |
|  | *GLRX2* | 1.98 | 2.53E-06 | 2.12 | 7.21E-07 | 1.07 | 5.17E-01 | 1.09 | 4.12E-01 | -1.11 | 3.08E-01 | -1.21 | 7.52E-02 |
|  | *GSN* | -1.45 | 1.67E-02 | -1.40 | 2.79E-02 | 1.04 | 8.08E-01 | -2.36 | 9.61E-06 | -1.89 | 2.77E-04 | 1.25 | 1.32E-01 |
|  | *HERPUD1* | 2.21 | 2.33E-04 | 2.10 | 4.55E-04 | -1.05 | 7.66E-01 | 1.32 | 1.22E-01 | 1.06 | 7.53E-01 | -1.25 | 2.09E-01 |
|  | *HIP1* | -1.47 | 2.45E-02 | -1.42 | 3.63E-02 | 1.03 | 8.49E-01 | -1.30 | 1.10E-01 | -1.43 | 3.49E-02 | -1.10 | 5.57E-01 |
|  | *HSP90B1* | -1.86 | 3.58E-07 | -2.02 | 6.33E-08 | -1.08 | 3.33E-01 | -1.02 | 7.99E-01 | -1.13 | 1.49E-01 | -1.10 | 2.28E-01 |
|  | *HSPA5* | -2.51 | 8.56E-10 | -2.98 | 5.55E-11 | -1.18 | 4.74E-02 | -1.10 | 2.64E-01 | -1.23 | 1.72E-02 | -1.12 | 1.58E-01 |
|  | *HTT* | -1.70 | 1.37E-05 | -2.11 | 1.26E-07 | -1.25 | 2.36E-02 | -1.40 | 1.40E-03 | -1.06 | 4.96E-01 | 1.32 | 6.54E-03 |
|  | *ID3* | -2.74 | 2.41E-08 | -2.33 | 3.06E-07 | 1.17 | 1.53E-01 | 1.43 | 3.89E-03 | -1.14 | 2.52E-01 | -1.62 | 2.80E-04 |
|  | *IFI6* | -2.95 | 2.57E-06 | -2.21 | 1.07E-04 | 1.34 | 8.79E-02 | -1.54 | 1.42E-02 | -1.19 | 3.01E-01 | 1.30 | 1.17E-01 |
|  | *IGFBP3* | 1.42 | 2.41E-01 | 1.27 | 4.13E-01 | -1.11 | 7.13E-01 | 2.24 | 1.23E-02 | 3.81 | 2.12E-04 | 1.70 | 8.21E-02 |
|  | *IL10* | -2.56 | 1.05E-03 | -2.36 | 2.27E-03 | 1.09 | 7.34E-01 | -1.39 | 1.85E-01 | 1.18 | 5.07E-01 | 1.64 | 5.47E-02 |
|  | *IL1A* | 3.86 | 6.67E-07 | 3.43 | 2.29E-06 | -1.12 | 5.24E-01 | 2.62 | 4.77E-05 | 3.21 | 4.65E-06 | 1.23 | 2.74E-01 |
|  | *IL6* | 1.91 | 1.49E-03 | 2.37 | 9.95E-05 | 1.24 | 2.36E-01 | 1.34 | 1.12E-01 | 2.32 | 1.27E-04 | 1.74 | 5.14E-03 |
|  | *KLF10* | 1.34 | 6.42E-02 | 1.37 | 5.11E-02 | 1.02 | 9.07E-01 | 1.40 | 3.82E-02 | 1.18 | 2.86E-01 | -1.19 | 2.71E-01 |
|  | *KRT8* | -1.45 | 4.08E-03 | -1.69 | 2.17E-04 | -1.16 | 2.02E-01 | 1.22 | 1.03E-01 | 1.72 | 1.53E-04 | 1.42 | 6.81E-03 |
|  | *LITAF* | 2.09 | 3.12E-07 | 2.76 | 2.56E-09 | 1.32 | 8.21E-03 | 1.51 | 3.30E-04 | 1.41 | 1.68E-03 | -1.07 | 4.73E-01 |
|  | *LOC285741* | 1.88 | 7.04E-04 | 2.06 | 1.83E-04 | 1.10 | 5.50E-01 | 1.75 | 1.96E-03 | 1.40 | 4.36E-02 | -1.25 | 1.64E-01 |
|  | *LRP1* | -1.34 | 4.37E-04 | -1.43 | 6.22E-05 | -1.06 | 3.84E-01 | -1.01 | 9.12E-01 | 1.30 | 1.15E-03 | 1.31 | 8.97E-04 |
|  | *MAP3K11* | -1.81 | 9.93E-04 | -1.90 | 4.81E-04 | -1.05 | 7.48E-01 | -1.84 | 8.21E-04 | -1.36 | 5.87E-02 | 1.35 | 6.18E-02 |
|  | *MCL1* | 1.69 | 5.98E-04 | 2.02 | 2.65E-05 | 1.20 | 1.68E-01 | 1.14 | 3.01E-01 | 1.23 | 1.17E-01 | 1.08 | 5.69E-01 |
|  | *MMP9* | -2.22 | 1.64E-08 | -2.45 | 2.71E-09 | -1.10 | 2.51E-01 | 1.38 | 9.87E-04 | 2.83 | 2.47E-10 | 2.04 | 8.40E-08 |
|  | *MX1* | -4.88 | 3.08E-06 | -4.38 | 7.57E-06 | 1.11 | 6.58E-01 | -2.22 | 3.57E-03 | -1.91 | 1.42E-02 | 1.16 | 5.33E-01 |
|  | *MYC* | 1.54 | 9.58E-03 | 1.97 | 2.35E-04 | 1.28 | 1.11E-01 | 1.13 | 4.30E-01 | -1.21 | 2.14E-01 | -1.36 | 5.05E-02 |
|  | *NCKAP1* | 2.26 | 2.48E-04 | 2.76 | 2.16E-05 | 1.23 | 2.71E-01 | 1.19 | 3.42E-01 | -1.25 | 2.32E-01 | -1.49 | 4.00E-02 |
|  | *NCSTN* | -2.08 | 1.54E-07 | -2.13 | 1.01E-07 | -1.02 | 8.10E-01 | 1.13 | 1.72E-01 | -1.21 | 4.52E-02 | -1.37 | 2.17E-03 |
|  | *NFKBIA* | -2.21 | 2.89E-07 | -1.94 | 3.24E-06 | 1.14 | 2.15E-01 | -1.65 | 8.98E-05 | -1.03 | 7.88E-01 | 1.61 | 1.63E-04 |
|  | *NME2* | 1.59 | 2.09E-04 | 1.87 | 7.35E-06 | 1.17 | 1.31E-01 | 1.05 | 6.06E-01 | -1.11 | 3.07E-01 | -1.17 | 1.32E-01 |
|  | *NOL3* | -1.50 | 3.72E-04 | -1.70 | 2.27E-05 | -1.13 | 2.11E-01 | 1.23 | 4.06E-02 | 1.21 | 5.76E-02 | -1.02 | 8.61E-01 |
|  | *NQO1* | 1.22 | 3.37E-01 | 1.18 | 4.18E-01 | -1.03 | 8.77E-01 | -1.16 | 4.70E-01 | -1.37 | 1.31E-01 | -1.18 | 4.10E-01 |
|  | *NR4A2* | 1.19 | 1.69E-01 | 1.68 | 4.99E-04 | 1.41 | 1.18E-02 | -1.20 | 1.54E-01 | 2.81 | 1.10E-07 | 3.36 | 9.74E-09 |
|  | *NRG1* | 2.15 | 1.01E-05 | 1.93 | 6.11E-05 | -1.11 | 4.02E-01 | 1.32 | 4.36E-02 | 1.46 | 8.05E-03 | 1.11 | 4.30E-01 |
|  | *PDCD10* | 2.01 | 1.33E-05 | 2.04 | 9.97E-06 | 1.02 | 8.89E-01 | 1.13 | 3.17E-01 | -1.04 | 7.42E-01 | -1.17 | 1.90E-01 |
|  | *PDCD4* | -2.07 | 1.95E-04 | -2.09 | 1.73E-04 | -1.01 | 9.58E-01 | -1.22 | 2.17E-01 | 1.07 | 6.72E-01 | 1.31 | 1.05E-01 |
|  | *PDCL3* | 1.63 | 1.11E-03 | 1.75 | 2.89E-04 | 1.08 | 5.51E-01 | 1.05 | 7.08E-01 | -1.21 | 1.39E-01 | -1.27 | 6.99E-02 |
|  | *PERP* | 1.85 | 3.18E-03 | 1.88 | 2.65E-03 | 1.02 | 9.35E-01 | 1.20 | 3.34E-01 | 1.43 | 6.39E-02 | 1.19 | 3.39E-01 |
|  | *PHLDA1* | 1.38 | 6.47E-02 | 1.84 | 1.56E-03 | 1.33 | 9.65E-02 | 1.19 | 3.11E-01 | 1.17 | 3.44E-01 | -1.01 | 9.44E-01 |
|  | *PHLDA2* | 1.45 | 2.69E-04 | 1.68 | 6.35E-06 | 1.16 | 9.38E-02 | 1.03 | 6.86E-01 | 1.00 | 9.82E-01 | -1.03 | 7.03E-01 |
|  | *PIM1* | -1.28 | 6.97E-03 | -1.16 | 8.22E-02 | 1.10 | 2.44E-01 | 1.45 | 2.16E-04 | 2.32 | 5.02E-09 | 1.60 | 1.83E-05 |
|  | *PPM1F* | -1.75 | 2.35E-04 | -1.97 | 2.83E-05 | -1.13 | 3.39E-01 | -1.53 | 2.73E-03 | -1.22 | 1.22E-01 | 1.25 | 8.10E-02 |
|  | *PPP1R13L* | -1.80 | 1.08E-05 | -1.85 | 5.82E-06 | -1.03 | 7.64E-01 | 1.18 | 1.06E-01 | 1.30 | 1.56E-02 | 1.10 | 3.45E-01 |
|  | *PPP1R15A* | 1.51 | 1.18E-02 | 1.31 | 8.13E-02 | -1.15 | 3.52E-01 | 1.30 | 9.50E-02 | 2.05 | 1.31E-04 | 1.58 | 6.49E-03 |
|  | *PRDX3* | 2.53 | 1.59E-03 | 2.62 | 1.20E-03 | 1.03 | 9.00E-01 | 1.32 | 2.79E-01 | -1.52 | 1.14E-01 | -2.01 | 1.24E-02 |
|  | *PSME3* | 1.38 | 2.32E-04 | 1.26 | 4.37E-03 | -1.10 | 2.03E-01 | -1.10 | 1.77E-01 | -1.35 | 4.15E-04 | -1.23 | 9.29E-03 |
|  | *PTGIS* | -3.70 | 1.26E-10 | -3.30 | 5.48E-10 | 1.12 | 2.73E-01 | -1.08 | 4.49E-01 | 1.02 | 8.64E-01 | 1.10 | 3.56E-01 |
|  | *PTGS2* | 1.29 | 7.81E-02 | 1.71 | 1.02E-03 | 1.32 | 5.60E-02 | -1.10 | 4.84E-01 | 2.03 | 6.04E-05 | 2.24 | 1.34E-05 |
|  | *PTPRF* | -1.26 | 4.48E-02 | -1.85 | 2.29E-05 | -1.46 | 2.55E-03 | 1.40 | 6.46E-03 | -1.04 | 7.00E-01 | -1.46 | 2.73E-03 |
|  | *RRM2B* | 2.18 | 1.05E-04 | 2.63 | 8.39E-06 | 1.21 | 2.46E-01 | 1.19 | 2.77E-01 | -1.04 | 7.90E-01 | -1.25 | 1.81E-01 |
|  | *SFN* | 2.80 | 1.44E-05 | 1.90 | 1.71E-03 | -1.47 | 4.13E-02 | -1.10 | 6.09E-01 | 1.13 | 4.98E-01 | 1.24 | 2.41E-01 |
|  | *SGK1* | 2.07 | 4.77E-03 | 2.19 | 2.74E-03 | 1.06 | 8.05E-01 | 1.21 | 4.06E-01 | 2.01 | 6.47E-03 | 1.66 | 3.89E-02 |
|  | *SH3GLB1* | 1.63 | 6.10E-04 | 2.17 | 3.76E-06 | 1.33 | 2.73E-02 | 1.18 | 1.89E-01 | -1.05 | 6.92E-01 | -1.23 | 9.42E-02 |
|  | *SMAD3* | -1.29 | 6.10E-03 | -1.60 | 1.81E-05 | -1.24 | 1.58E-02 | 1.02 | 7.74E-01 | 1.50 | 1.05E-04 | 1.46 | 1.99E-04 |
|  | *SRC* | -1.83 | 1.31E-04 | -1.61 | 1.24E-03 | 1.14 | 3.23E-01 | -1.41 | 1.31E-02 | 1.15 | 2.78E-01 | 1.62 | 1.11E-03 |
|  | *SRGN* | 2.10 | 3.03E-03 | 1.71 | 2.39E-02 | -1.23 | 3.51E-01 | 1.46 | 1.01E-01 | -1.31 | 2.24E-01 | -1.91 | 7.83E-03 |
|  | *STAG3L3* | -2.04 | 1.98E-02 | -1.94 | 2.85E-02 | 1.05 | 8.63E-01 | -1.97 | 2.57E-02 | -1.40 | 2.42E-01 | 1.41 | 2.37E-01 |
|  | *STEAP3* | -1.46 | 2.46E-06 | -1.26 | 6.59E-04 | 1.16 | 1.62E-02 | -1.02 | 6.85E-01 | 1.48 | 1.61E-06 | 1.51 | 7.36E-07 |
|  | *TAF9* | 1.80 | 5.56E-03 | 2.04 | 1.21E-03 | 1.14 | 5.00E-01 | 1.34 | 1.37E-01 | -1.14 | 4.88E-01 | -1.52 | 3.62E-02 |
|  | *TIMP3* | -1.59 | 3.23E-02 | -1.94 | 3.92E-03 | -1.22 | 3.37E-01 | -1.61 | 2.90E-02 | -3.04 | 3.01E-05 | -1.89 | 5.48E-03 |
|  | *TLR4* | -1.89 | 3.11E-06 | -1.70 | 3.06E-05 | 1.11 | 2.77E-01 | -1.20 | 7.44E-02 | -1.19 | 8.68E-02 | 1.01 | 9.35E-01 |
|  | *TNFRSF10B* | -1.72 | 5.76E-07 | -1.91 | 4.52E-08 | -1.11 | 1.63E-01 | -1.26 | 5.41E-03 | -1.06 | 4.36E-01 | 1.19 | 2.95E-02 |
|  | *TNFRSF25* | -2.08 | 1.53E-04 | -1.91 | 5.22E-04 | 1.09 | 5.85E-01 | -1.87 | 7.22E-04 | 1.13 | 4.30E-01 | 2.11 | 1.22E-04 |
|  | *TNFSF14* | -3.41 | 5.48E-04 | -2.75 | 2.84E-03 | 1.24 | 4.70E-01 | -1.51 | 1.75E-01 | 1.33 | 3.49E-01 | 2.00 | 2.89E-02 |
|  | *TNFSF18* | 2.01 | 3.81E-04 | 2.47 | 2.30E-05 | 1.23 | 2.11E-01 | 1.25 | 1.82E-01 | -1.20 | 2.64E-01 | -1.50 | 2.04E-02 |
|  | *TRIB3* | 2.04 | 8.56E-06 | 2.18 | 2.63E-06 | 1.07 | 5.61E-01 | -1.31 | 3.07E-02 | 1.09 | 4.81E-01 | 1.43 | 6.68E-03 |
|  | *TXNDC5* | -2.31 | 3.12E-07 | -2.64 | 3.70E-08 | -1.14 | 2.30E-01 | 1.06 | 5.91E-01 | -1.32 | 1.82E-02 | -1.40 | 5.59E-03 |
|  | *TXNIP* | -4.47 | 1.56E-10 | -4.87 | 6.33E-11 | -1.09 | 4.75E-01 | 1.25 | 7.37E-02 | 1.15 | 2.58E-01 | -1.09 | 4.74E-01 |
|  | *XAF1* | -2.41 | 4.52E-04 | -2.00 | 3.37E-03 | 1.20 | 3.78E-01 | -1.44 | 9.51E-02 | -1.34 | 1.70E-01 | 1.07 | 7.44E-01 |
|  | *YARS* | 2.03 | 3.40E-07 | 2.09 | 1.94E-07 | 1.03 | 7.57E-01 | -1.01 | 9.23E-01 | 1.38 | 2.28E-03 | 1.39 | 1.83E-03 |
|  | *YWHAB* | 1.69 | 4.30E-04 | 1.69 | 4.39E-04 | -1.00 | 9.93E-01 | 1.03 | 7.85E-01 | -1.20 | 1.50E-01 | -1.24 | 9.19E-02 |
|  | *YWHAE* | -2.31 | 2.76E-03 | -2.23 | 3.82E-03 | 1.04 | 8.85E-01 | -1.11 | 6.72E-01 | 1.25 | 3.65E-01 | 1.39 | 1.90E-01 |
|  | *ZAK* | 1.81 | 1.64E-04 | 1.94 | 4.91E-05 | 1.07 | 5.84E-01 | 1.29 | 5.58E-02 | 1.45 | 8.48E-03 | 1.12 | 3.75E-01 |
|  |  |  |  |  |  |  |  |  |  |  |  |  |  |
| **Adhesion/cell-cell contact/extracellular matrix** | *ADAM15* | -1.88 | 2.27E-04 | -2.52 | 2.77E-06 | -1.34 | 4.89E-02 | -2.15 | 2.75E-05 | -2.05 | 5.70E-05 | 1.05 | 7.36E-01 |
|  | *ATP2A2* | -1.01 | 9.60E-01 | -1.37 | 1.03E-02 | -1.36 | 1.15E-02 | 1.11 | 3.54E-01 | 1.04 | 7.12E-01 | -1.06 | 5.72E-01 |
|  | *BMP1* | -1.73 | 4.98E-06 | -1.57 | 5.83E-05 | 1.11 | 2.50E-01 | -1.27 | 1.29E-02 | -1.23 | 2.49E-02 | 1.03 | 7.57E-01 |
|  | *CCL5* | -1.57 | 1.69E-03 | -1.67 | 5.41E-04 | -1.06 | 6.15E-01 | 1.32 | 3.68E-02 | 2.11 | 9.10E-06 | 1.60 | 1.18E-03 |
|  | *CD34* | -1.73 | 1.75E-04 | -1.97 | 1.71E-05 | -1.14 | 2.90E-01 | -1.37 | 1.56E-02 | -1.82 | 6.99E-05 | -1.33 | 2.42E-02 |
|  | *CD58* | 1.59 | 3.63E-03 | 1.58 | 3.97E-03 | -1.01 | 9.69E-01 | -1.01 | 9.48E-01 | -1.29 | 8.24E-02 | -1.28 | 9.31E-02 |
|  | *CD93* | -1.93 | 1.35E-04 | -1.85 | 2.53E-04 | 1.04 | 7.78E-01 | -1.76 | 6.05E-04 | -1.23 | 1.41E-01 | 1.43 | 1.79E-02 |
|  | *CDH15* | -1.31 | 2.03E-03 | -1.30 | 2.56E-03 | 1.01 | 9.18E-01 | 2.05 | 1.98E-08 | 1.68 | 2.10E-06 | -1.22 | 1.63E-02 |
|  | *CELSR3* | -1.83 | 1.37E-05 | -2.01 | 2.04E-06 | -1.10 | 3.54E-01 | -1.17 | 1.49E-01 | 1.05 | 6.30E-01 | 1.23 | 6.08E-02 |
|  | *CERCAM* | -1.81 | 3.06E-04 | -1.92 | 1.22E-04 | -1.06 | 6.80E-01 | -1.06 | 6.49E-01 | 1.16 | 2.87E-01 | 1.23 | 1.36E-01 |
|  | *CLDN11* | -1.06 | 6.48E-01 | -1.22 | 9.86E-02 | -1.16 | 2.17E-01 | 1.48 | 3.51E-03 | -1.49 | 2.79E-03 | -2.21 | 2.21E-06 |
|  | *CLDN14* | -2.27 | 6.17E-06 | -2.52 | 1.26E-06 | -1.11 | 4.26E-01 | -1.34 | 3.52E-02 | -1.77 | 3.62E-04 | -1.31 | 5.00E-02 |
|  | *CLSTN1* | -1.73 | 1.07E-06 | -2.05 | 2.17E-08 | -1.19 | 3.79E-02 | -1.07 | 3.92E-01 | -1.01 | 8.84E-01 | 1.06 | 4.75E-01 |
|  | *CNTNAP1* | -1.65 | 5.13E-06 | -1.62 | 9.02E-06 | 1.02 | 7.82E-01 | 1.60 | 1.21E-05 | 1.37 | 9.14E-04 | -1.17 | 6.05E-02 |
|  | *COL13A1* | -1.67 | 1.09E-06 | -1.56 | 7.35E-06 | 1.07 | 3.42E-01 | 1.04 | 5.72E-01 | 1.76 | 2.89E-07 | 1.69 | 8.30E-07 |
|  | *COL5A2* | -2.46 | 1.31E-07 | -2.95 | 8.08E-09 | -1.20 | 1.05E-01 | -1.16 | 1.86E-01 | -1.53 | 9.56E-04 | -1.32 | 1.94E-02 |
|  | *COL6A1* | -1.89 | 2.06E-07 | -1.85 | 3.38E-07 | 1.02 | 7.85E-01 | -1.07 | 4.00E-01 | 1.23 | 1.69E-02 | 1.32 | 2.58E-03 |
|  | *COL6A2* | -1.73 | 4.39E-05 | -1.79 | 2.34E-05 | -1.03 | 7.70E-01 | 1.16 | 1.62E-01 | 1.07 | 5.12E-01 | -1.08 | 4.40E-01 |
|  | *COL8A1* | -1.74 | 1.50E-03 | -2.78 | 1.92E-06 | -1.60 | 5.55E-03 | 1.29 | 1.03E-01 | -1.20 | 2.40E-01 | -1.55 | 8.89E-03 |
|  | *CTNNAL1* | -2.38 | 8.52E-07 | -2.62 | 1.90E-07 | -1.10 | 4.20E-01 | 1.38 | 1.44E-02 | -1.29 | 4.31E-02 | -1.78 | 1.19E-04 |
|  | *DGCR2* | -1.88 | 1.08E-04 | -2.01 | 3.68E-05 | -1.07 | 6.21E-01 | -1.31 | 5.00E-02 | 1.01 | 9.66E-01 | 1.32 | 4.59E-02 |
|  | *DST* | -1.11 | 4.35E-01 | -1.34 | 3.62E-02 | -1.21 | 1.60E-01 | -1.18 | 2.14E-01 | 1.35 | 3.06E-02 | 1.59 | 1.90E-03 |
|  | *FAT4* | -1.93 | 2.96E-07 | -2.26 | 1.26E-08 | -1.17 | 7.59E-02 | 1.10 | 2.54E-01 | -1.03 | 7.15E-01 | -1.14 | 1.39E-01 |
|  | *GPNMB* | 2.63 | 7.08E-09 | 3.11 | 5.89E-10 | 1.18 | 1.00E-01 | -1.06 | 5.69E-01 | -1.16 | 1.40E-01 | -1.10 | 3.47E-01 |
|  | *HSD17B12* | -1.07 | 6.34E-01 | -1.24 | 1.13E-01 | -1.17 | 2.53E-01 | -1.12 | 4.08E-01 | -2.00 | 4.78E-05 | -1.79 | 3.01E-04 |
|  | *IGFBP7* | -1.69 | 5.91E-03 | -1.67 | 6.63E-03 | 1.01 | 9.59E-01 | 1.64 | 8.80E-03 | -1.26 | 1.90E-01 | -2.06 | 4.33E-04 |
|  | *IL32* | 1.08 | 5.18E-01 | -1.14 | 2.87E-01 | -1.24 | 9.62E-02 | 1.45 | 6.35E-03 | 1.86 | 6.70E-05 | 1.28 | 5.38E-02 |
|  | *ITGA10* | -3.08 | 1.68E-05 | -2.63 | 9.50E-05 | 1.17 | 4.24E-01 | -1.48 | 5.72E-02 | -1.88 | 4.46E-03 | -1.27 | 2.39E-01 |
|  | *ITGA11* | -2.34 | 2.41E-08 | -2.53 | 6.03E-09 | -1.08 | 3.87E-01 | -1.04 | 6.38E-01 | -1.41 | 1.32E-03 | -1.35 | 3.80E-03 |
|  | *ITGAE* | 1.55 | 1.13E-03 | 1.43 | 5.31E-03 | -1.08 | 4.94E-01 | -1.04 | 7.33E-01 | -1.30 | 3.26E-02 | -1.25 | 6.46E-02 |
|  | *ITGB4* | -4.08 | 1.51E-08 | -4.59 | 4.34E-09 | -1.13 | 4.28E-01 | -1.12 | 4.54E-01 | -1.03 | 8.54E-01 | 1.09 | 5.70E-01 |
|  | *ITGB5* | -1.72 | 1.16E-04 | -2.32 | 5.45E-07 | -1.34 | 1.59E-02 | -1.10 | 4.13E-01 | -1.21 | 1.02E-01 | -1.10 | 3.87E-01 |
|  | *ITGBL1* | -1.59 | 9.22E-04 | -1.92 | 2.63E-05 | -1.21 | 1.20E-01 | 1.18 | 1.79E-01 | 1.11 | 3.64E-01 | -1.06 | 6.46E-01 |
|  | *LAMB2* | -2.54 | 1.47E-05 | -2.80 | 4.21E-06 | -1.10 | 5.45E-01 | -1.55 | 1.26E-02 | -1.25 | 1.76E-01 | 1.24 | 1.90E-01 |
|  | *LAMC1* | -1.66 | 3.67E-06 | -2.07 | 2.28E-08 | -1.25 | 1.07E-02 | -1.09 | 2.84E-01 | -1.12 | 1.62E-01 | -1.03 | 7.27E-01 |
|  | *LGALS3BP* | -1.84 | 4.13E-04 | -1.98 | 1.29E-04 | -1.08 | 6.04E-01 | 1.02 | 8.77E-01 | -1.08 | 6.05E-01 | -1.10 | 5.03E-01 |
|  | *LMO7* | 1.35 | 1.00E-02 | 1.20 | 9.32E-02 | -1.12 | 2.84E-01 | 1.21 | 7.96E-02 | 2.05 | 1.72E-06 | 1.69 | 7.65E-05 |
|  | *LPXN* | 1.74 | 4.42E-05 | 1.86 | 1.15E-05 | 1.07 | 5.28E-01 | 1.30 | 1.99E-02 | 1.38 | 6.66E-03 | 1.05 | 6.15E-01 |
|  | *MCAM* | -1.50 | 2.39E-03 | -1.66 | 3.13E-04 | -1.11 | 3.73E-01 | -1.90 | 2.57E-05 | -1.54 | 1.31E-03 | 1.23 | 8.82E-02 |
|  | *MPZL2* | -1.79 | 5.74E-05 | -2.11 | 2.79E-06 | -1.18 | 1.58E-01 | -1.08 | 4.80E-01 | -1.14 | 2.45E-01 | -1.06 | 6.36E-01 |
|  | *MTSS1* | -1.34 | 1.36E-02 | -1.28 | 3.65E-02 | 1.05 | 6.39E-01 | -1.11 | 3.40E-01 | 1.48 | 1.91E-03 | 1.65 | 2.17E-04 |
|  | *NFKB2* | -1.42 | 2.97E-03 | -1.72 | 4.49E-05 | -1.21 | 7.28E-02 | -1.11 | 3.01E-01 | 1.67 | 7.67E-05 | 1.87 | 8.24E-06 |
|  | *OLFML2A* | -2.58 | 6.53E-10 | -2.75 | 2.36E-10 | -1.06 | 4.51E-01 | 1.34 | 1.99E-03 | 1.23 | 1.98E-02 | -1.09 | 3.05E-01 |
|  | *P4HA1* | -2.38 | 9.94E-09 | -2.50 | 4.25E-09 | -1.05 | 5.82E-01 | 1.18 | 8.06E-02 | 1.22 | 3.47E-02 | 1.04 | 6.70E-01 |
|  | *PARP12* | -2.40 | 2.29E-05 | -2.13 | 1.14E-04 | 1.12 | 4.62E-01 | -1.56 | 9.59E-03 | -1.10 | 5.51E-01 | 1.42 | 3.43E-02 |
|  | *PCDHB9* | -2.24 | 3.71E-03 | -2.17 | 4.88E-03 | 1.03 | 9.02E-01 | -1.56 | 8.43E-02 | 1.11 | 6.81E-01 | 1.72 | 3.75E-02 |
|  | *PDPN* | -2.45 | 1.28E-08 | -2.19 | 9.40E-08 | 1.12 | 2.40E-01 | -1.63 | 4.43E-05 | -1.74 | 1.12E-05 | -1.06 | 5.17E-01 |
|  | *PECAM1* | -1.70 | 7.78E-03 | -1.80 | 3.63E-03 | -1.06 | 7.32E-01 | -1.34 | 1.16E-01 | -1.86 | 2.46E-03 | -1.39 | 7.85E-02 |
|  | *PODXL* | -1.19 | 7.68E-02 | -1.67 | 2.60E-05 | -1.41 | 1.56E-03 | -1.14 | 1.64E-01 | 1.29 | 1.14E-02 | 1.48 | 4.62E-04 |
|  | *RGMB* | 1.62 | 2.31E-06 | 1.76 | 2.39E-07 | 1.09 | 2.39E-01 | -1.06 | 4.50E-01 | 1.43 | 9.06E-05 | 1.51 | 1.76E-05 |
|  | *SIPA1* | -1.68 | 9.07E-04 | -2.04 | 3.71E-05 | -1.21 | 1.61E-01 | -1.30 | 5.84E-02 | 1.29 | 6.64E-02 | 1.68 | 8.87E-04 |
|  | *SIRPA* | -1.48 | 4.80E-03 | -1.60 | 1.15E-03 | -1.08 | 5.28E-01 | -1.10 | 4.27E-01 | -1.22 | 1.18E-01 | -1.11 | 4.19E-01 |
|  | *SPOCK1* | -3.32 | 2.79E-09 | -3.80 | 4.95E-10 | -1.15 | 2.31E-01 | 1.57 | 7.26E-04 | 1.38 | 9.46E-03 | -1.14 | 2.61E-01 |
|  | *SPP1* | 4.61 | 1.16E-08 | 5.50 | 2.12E-09 | 1.19 | 2.70E-01 | 1.32 | 9.42E-02 | -1.18 | 3.10E-01 | -1.55 | 1.15E-02 |
|  | *TESC* | 2.04 | 4.97E-05 | 2.50 | 2.34E-06 | 1.22 | 1.52E-01 | -2.24 | 1.15E-05 | -2.22 | 1.37E-05 | 1.01 | 9.34E-01 |
|  | *TGFB1I1* | -1.53 | 2.42E-04 | -1.54 | 2.31E-04 | -1.00 | 9.83E-01 | 1.07 | 4.70E-01 | 1.32 | 9.01E-03 | 1.23 | 4.20E-02 |
|  | *TGFBI* | -1.47 | 3.16E-04 | -1.69 | 1.04E-05 | -1.15 | 1.27E-01 | 1.81 | 2.32E-06 | 1.58 | 5.65E-05 | -1.15 | 1.36E-01 |
|  | *THBS3* | -1.79 | 2.78E-03 | -1.47 | 3.27E-02 | 1.21 | 2.65E-01 | -1.21 | 2.64E-01 | 1.09 | 5.99E-01 | 1.33 | 1.09E-01 |
|  | *TPM1* | -1.71 | 1.33E-03 | -1.73 | 1.12E-03 | -1.01 | 9.38E-01 | -1.16 | 3.03E-01 | -1.37 | 3.85E-02 | -1.18 | 2.57E-01 |
|  | *VWF* | -3.18 | 1.50E-05 | -3.42 | 6.99E-06 | -1.08 | 7.13E-01 | -3.80 | 2.43E-06 | -6.40 | 2.29E-08 | -1.68 | 1.69E-02 |
|  | *WISP2* | -1.17 | 1.10E-02 | -1.23 | 1.72E-03 | -1.05 | 4.10E-01 | 1.86 | 1.95E-09 | 1.04 | 4.54E-01 | -1.78 | 6.02E-09 |
|  |  |  |  |  |  |  |  |  |  |  |  |  |  |
| **Cytoskeleton** | *ABLIM1* | 1.27 | 1.78E-01 | 1.36 | 8.92E-02 | 1.07 | 6.98E-01 | 1.24 | 2.18E-01 | -1.67 | 7.81E-03 | -2.07 | 4.61E-04 |
|  | *AGFG1* | -1.24 | 8.15E-02 | -1.38 | 1.25E-02 | -1.11 | 3.65E-01 | 1.25 | 7.52E-02 | 1.50 | 2.57E-03 | 1.21 | 1.25E-01 |
|  | *ALDOA* | -2.06 | 9.10E-09 | -2.67 | 7.12E-11 | -1.29 | 2.31E-03 | -1.01 | 9.40E-01 | 1.02 | 7.71E-01 | 1.03 | 7.15E-01 |
|  | *ARAP3* | -1.51 | 9.96E-04 | -1.83 | 1.72E-05 | -1.22 | 7.77E-02 | -1.62 | 2.28E-04 | -1.57 | 4.34E-04 | 1.03 | 7.75E-01 |
|  | *ARPC1B* | 2.16 | 2.92E-04 | 2.92 | 7.04E-06 | 1.35 | 9.62E-02 | 1.16 | 3.92E-01 | 1.11 | 5.59E-01 | -1.05 | 7.82E-01 |
|  | *CALD1* | -1.23 | 3.98E-01 | -1.51 | 9.82E-02 | -1.23 | 3.91E-01 | -1.28 | 3.04E-01 | 1.46 | 1.27E-01 | 1.88 | 1.60E-02 |
|  | *CDC42* | 1.78 | 4.76E-03 | 2.29 | 2.16E-04 | 1.28 | 1.80E-01 | 1.24 | 2.50E-01 | -1.06 | 7.59E-01 | -1.31 | 1.51E-01 |
|  | *CDC42BPB* | -1.50 | 4.39E-03 | -1.71 | 4.19E-04 | -1.14 | 3.05E-01 | -1.13 | 3.52E-01 | 1.32 | 4.08E-02 | 1.48 | 5.43E-03 |
|  | *CDC42EP5* | -1.37 | 1.93E-02 | -1.25 | 8.06E-02 | 1.09 | 4.83E-01 | 1.24 | 9.78E-02 | -1.09 | 5.06E-01 | -1.34 | 2.61E-02 |
|  | *CENPA* | 1.71 | 1.27E-03 | 1.13 | 3.86E-01 | -1.51 | 9.00E-03 | 1.02 | 8.69E-01 | -1.17 | 2.90E-01 | -1.19 | 2.25E-01 |
|  | *CKS2* | 1.82 | 4.42E-05 | 1.80 | 5.34E-05 | -1.01 | 9.30E-01 | 1.25 | 5.75E-02 | -1.10 | 3.93E-01 | -1.38 | 9.46E-03 |
|  | *CRIPAK* | -1.25 | 8.58E-02 | -1.42 | 1.09E-02 | -1.13 | 3.21E-01 | -1.18 | 1.91E-01 | 1.19 | 1.78E-01 | 1.41 | 1.29E-02 |
|  | *CYTH2* | -2.07 | 9.75E-06 | -1.90 | 4.29E-05 | 1.09 | 4.86E-01 | -1.65 | 5.69E-04 | -1.38 | 1.57E-02 | 1.20 | 1.49E-01 |
|  | *EHD2* | -2.25 | 1.32E-04 | -2.04 | 4.62E-04 | 1.10 | 5.76E-01 | -1.38 | 6.86E-02 | -1.08 | 6.59E-01 | 1.28 | 1.54E-01 |
|  | *INF2* | -2.05 | 3.37E-05 | -1.88 | 1.31E-04 | 1.09 | 5.34E-01 | -1.34 | 3.69E-02 | 1.03 | 8.02E-01 | 1.39 | 2.19E-02 |
|  | *LIMCH1* | -2.26 | 4.94E-07 | -2.39 | 1.89E-07 | -1.06 | 5.99E-01 | -1.05 | 6.68E-01 | -1.25 | 4.94E-02 | -1.20 | 1.12E-01 |
|  | *LOC399942* | 2.97 | 2.47E-04 | 3.00 | 2.26E-04 | 1.01 | 9.67E-01 | 2.08 | 6.79E-03 | 1.09 | 7.23E-01 | -1.91 | 1.47E-02 |
|  | *MACF1* | -1.11 | 5.66E-01 | -1.72 | 8.34E-03 | -1.55 | 2.87E-02 | -1.31 | 1.53E-01 | 1.07 | 7.11E-01 | 1.41 | 7.82E-02 |
|  | *MID1IP1* | 1.86 | 1.67E-07 | 1.88 | 1.23E-07 | 1.01 | 8.60E-01 | 1.46 | 9.65E-05 | 1.46 | 9.92E-05 | -1.00 | 9.90E-01 |
|  | *RAC3* | -1.73 | 2.44E-04 | -1.79 | 1.29E-04 | -1.04 | 7.76E-01 | -1.13 | 3.36E-01 | 1.27 | 5.94E-02 | 1.44 | 7.66E-03 |
|  | *RAN* | 1.72 | 2.47E-03 | 1.79 | 1.35E-03 | 1.04 | 7.89E-01 | 1.33 | 7.92E-02 | -1.07 | 6.61E-01 | -1.42 | 3.32E-02 |
|  | *SDCBP* | 2.98 | 1.95E-03 | 3.61 | 4.75E-04 | 1.21 | 5.33E-01 | 1.11 | 7.35E-01 | -1.51 | 1.86E-01 | -1.68 | 1.03E-01 |
|  | *SPTAN1* | -2.03 | 3.20E-06 | -2.21 | 7.05E-07 | -1.09 | 4.39E-01 | -1.27 | 3.57E-02 | -1.43 | 3.77E-03 | -1.12 | 3.06E-01 |
|  | *TACC3* | -1.39 | 3.06E-02 | -1.93 | 1.80E-04 | -1.39 | 3.04E-02 | -1.36 | 4.12E-02 | 1.04 | 7.63E-01 | 1.42 | 2.21E-02 |
|  | *TTLL3* | -1.90 | 1.78E-03 | -1.55 | 2.24E-02 | 1.23 | 2.60E-01 | -1.49 | 3.44E-02 | 1.02 | 9.04E-01 | 1.52 | 2.68E-02 |
|  | *TUBB6* | 2.07 | 2.24E-04 | 2.02 | 3.14E-04 | -1.02 | 8.79E-01 | 1.24 | 1.94E-01 | 1.12 | 4.79E-01 | -1.10 | 5.39E-01 |
|  | *UBE2C* | -1.19 | 9.31E-02 | -2.00 | 1.20E-06 | -1.68 | 4.28E-05 | 1.37 | 4.82E-03 | 1.89 | 3.71E-06 | 1.38 | 3.65E-03 |
|  |  |  |  |  |  |  |  |  |  |  |  |  |  |
| **Cell migration** | *ABI3* | -1.80 | 1.09E-03 | -2.38 | 2.00E-05 | -1.32 | 8.24E-02 | -1.50 | 1.56E-02 | -1.15 | 3.81E-01 | 1.31 | 9.31E-02 |
|  | *CLIC4* | 1.54 | 2.83E-05 | 2.18 | 9.65E-09 | 1.41 | 3.51E-04 | 1.27 | 6.47E-03 | 1.15 | 1.00E-01 | -1.11 | 1.95E-01 |
|  | *HBEGF* | 1.06 | 5.22E-01 | 1.39 | 1.65E-03 | 1.31 | 7.01E-03 | -1.48 | 3.67E-04 | 1.49 | 2.74E-04 | 2.21 | 5.40E-08 |
|  | *PRSS3* | 1.07 | 4.89E-01 | 1.15 | 1.65E-01 | 1.07 | 4.68E-01 | 1.40 | 2.09E-03 | 2.21 | 1.16E-07 | 1.58 | 1.33E-04 |
|  | *PTP4A1* | 1.93 | 3.43E-06 | 2.15 | 4.12E-07 | 1.12 | 2.78E-01 | 1.22 | 6.35E-02 | 1.07 | 5.26E-01 | -1.14 | 2.00E-01 |
|  | *SEMA3F* | -1.85 | 3.06E-04 | -1.99 | 9.99E-05 | -1.07 | 6.15E-01 | -1.30 | 7.20E-02 | 1.05 | 7.16E-01 | 1.37 | 3.50E-02 |
|  | *SERPINE2* | 5.24 | 7.33E-09 | 5.56 | 4.23E-09 | 1.06 | 7.20E-01 | 2.03 | 4.07E-04 | 1.64 | 7.11E-03 | -1.23 | 2.14E-01 |
|  | *SPAG9* | 1.93 | 9.84E-05 | 1.97 | 7.08E-05 | 1.02 | 8.81E-01 | 1.23 | 1.35E-01 | 1.04 | 7.57E-01 | -1.18 | 2.27E-01 |
|  | *TNS3* | -1.16 | 2.20E-01 | -1.47 | 3.57E-03 | -1.27 | 5.22E-02 | 1.60 | 7.08E-04 | 1.00 | 9.79E-01 | -1.59 | 7.50E-04 |
|  | *TRIB1* | 1.32 | 1.98E-02 | 1.67 | 1.65E-04 | 1.27 | 4.28E-02 | -1.29 | 2.90E-02 | 1.57 | 5.47E-04 | 2.03 | 3.61E-06 |
|  |  |  |  |  |  |  |  |  |  |  |  |  |  |
| **Lipid metabolic process** | *ABCA2* | -2.00 | 4.40E-05 | -2.21 | 8.49E-06 | -1.11 | 4.40E-01 | 1.04 | 7.45E-01 | 1.28 | 7.43E-02 | 1.22 | 1.35E-01 |
|  | *ABHD5* | 2.03 | 3.52E-04 | 2.42 | 3.33E-05 | 1.19 | 2.91E-01 | -1.06 | 7.43E-01 | 1.04 | 8.14E-01 | 1.10 | 5.74E-01 |
|  | *ACAD11* | -1.90 | 1.47E-06 | -1.90 | 1.46E-06 | -1.00 | 9.97E-01 | 1.20 | 5.64E-02 | 1.32 | 7.46E-03 | 1.09 | 3.42E-01 |
|  | *ACADVL* | -2.22 | 2.47E-03 | -2.41 | 1.07E-03 | -1.09 | 7.11E-01 | -1.55 | 7.01E-02 | -1.12 | 6.23E-01 | 1.38 | 1.71E-01 |
|  | *ACAT2* | 1.85 | 1.41E-03 | 1.02 | 9.11E-01 | -1.82 | 1.81E-03 | 1.08 | 6.56E-01 | -1.51 | 2.05E-02 | -1.63 | 7.80E-03 |
|  | *ACER3* | -2.62 | 1.51E-07 | -2.34 | 8.92E-07 | 1.12 | 3.39E-01 | -1.44 | 6.06E-03 | 1.10 | 4.28E-01 | 1.58 | 1.00E-03 |
|  | *ACOX2* | 2.39 | 1.43E-06 | 2.13 | 8.74E-06 | -1.12 | 3.70E-01 | 1.25 | 9.27E-02 | 1.22 | 1.19E-01 | -1.02 | 8.89E-01 |
|  | *AGPAT4* | -1.82 | 1.14E-04 | -1.81 | 1.23E-04 | 1.00 | 9.71E-01 | -1.33 | 3.19E-02 | -1.22 | 1.26E-01 | 1.09 | 4.79E-01 |
|  | *AGPAT9* | 1.20 | 1.36E-01 | 1.58 | 9.63E-04 | 1.32 | 2.88E-02 | -1.38 | 1.18E-02 | -1.26 | 6.19E-02 | 1.10 | 4.28E-01 |
|  | *AKR1B10* | 2.06 | 7.96E-07 | 1.77 | 1.63E-05 | -1.16 | 1.43E-01 | 1.06 | 5.59E-01 | 1.28 | 2.00E-02 | 1.21 | 6.62E-02 |
|  | *AKR1D1* | -1.86 | 1.50E-03 | -1.74 | 3.62E-03 | 1.07 | 6.96E-01 | -1.29 | 1.42E-01 | 1.10 | 5.68E-01 | 1.42 | 4.84E-02 |
|  | *ALG1* | 1.66 | 3.18E-05 | 1.81 | 4.49E-06 | 1.09 | 3.53E-01 | -1.04 | 6.97E-01 | -1.23 | 3.76E-02 | -1.19 | 8.08E-02 |
|  | *ALG13* | 1.86 | 1.25E-03 | 1.89 | 1.06E-03 | 1.01 | 9.41E-01 | 1.16 | 3.69E-01 | -1.11 | 5.31E-01 | -1.29 | 1.36E-01 |
|  | *ATP8B4* | -1.98 | 1.84E-07 | -1.53 | 7.64E-05 | 1.29 | 6.59E-03 | -1.04 | 6.05E-01 | 1.32 | 4.08E-03 | 1.38 | 1.27E-03 |
|  | *CNBP* | 2.10 | 1.72E-03 | 2.42 | 3.61E-04 | 1.15 | 4.91E-01 | 1.30 | 2.08E-01 | -1.15 | 4.93E-01 | -1.50 | 6.03E-02 |
|  | *COL4A3BP* | -1.63 | 1.09E-03 | -1.84 | 1.24E-04 | -1.13 | 3.38E-01 | -1.36 | 2.45E-02 | 1.25 | 9.41E-02 | 1.70 | 5.12E-04 |
|  | *CPS1* | -1.85 | 5.32E-07 | -1.74 | 1.99E-06 | 1.06 | 4.92E-01 | 1.61 | 1.37E-05 | 1.29 | 6.15E-03 | -1.25 | 1.18E-02 |
|  | *CYP26B1* | 1.75 | 2.30E-03 | 2.04 | 2.83E-04 | 1.16 | 3.58E-01 | -1.30 | 1.11E-01 | -1.44 | 3.36E-02 | -1.10 | 5.40E-01 |
|  | *DPM1* | 2.14 | 8.48E-05 | 1.83 | 8.16E-04 | -1.17 | 3.17E-01 | 1.08 | 6.12E-01 | -1.13 | 4.45E-01 | -1.22 | 2.11E-01 |
|  | *EBP* | 1.68 | 1.21E-03 | 1.17 | 2.51E-01 | -1.43 | 1.62E-02 | 1.01 | 9.23E-01 | -1.14 | 3.40E-01 | -1.16 | 2.95E-01 |
|  | *ELOVL6* | 1.66 | 3.23E-04 | 1.45 | 4.48E-03 | -1.14 | 2.52E-01 | 1.11 | 3.66E-01 | -1.10 | 4.26E-01 | -1.22 | 9.84E-02 |
|  | *ETNK1* | 1.89 | 2.53E-06 | 1.88 | 2.89E-06 | -1.01 | 9.46E-01 | 1.14 | 1.89E-01 | 1.09 | 3.61E-01 | -1.04 | 6.75E-01 |
|  | *FABP5L2* | 2.15 | 2.92E-06 | 1.94 | 1.75E-05 | -1.11 | 3.87E-01 | 1.39 | 1.07E-02 | 1.13 | 2.95E-01 | -1.22 | 9.37E-02 |
|  | *FAR1* | -2.21 | 2.47E-06 | -2.50 | 3.39E-07 | -1.13 | 3.03E-01 | -1.20 | 1.31E-01 | 1.28 | 5.19E-02 | 1.54 | 1.77E-03 |
|  | *FDFT1* | 2.16 | 3.61E-07 | 1.67 | 6.20E-05 | -1.29 | 1.82E-02 | 1.05 | 6.01E-01 | -1.29 | 1.75E-02 | -1.36 | 5.56E-03 |
|  | *GBA* | 1.41 | 3.61E-03 | 1.88 | 7.42E-06 | 1.34 | 1.04E-02 | 1.04 | 7.02E-01 | -1.14 | 2.11E-01 | -1.19 | 1.09E-01 |
|  | *GLA* | 1.42 | 3.65E-04 | 1.43 | 2.80E-04 | 1.01 | 9.06E-01 | -1.07 | 4.18E-01 | -1.11 | 2.06E-01 | -1.04 | 6.35E-01 |
|  | *GOT1* | 2.20 | 5.08E-08 | 2.39 | 1.18E-08 | 1.08 | 3.77E-01 | 1.08 | 3.75E-01 | 1.06 | 5.35E-01 | -1.02 | 7.85E-01 |
|  | *HSD3B7* | -1.54 | 3.65E-03 | -1.48 | 7.46E-03 | 1.04 | 7.49E-01 | 1.17 | 2.34E-01 | 1.65 | 1.08E-03 | 1.41 | 1.61E-02 |
|  | *IDI1* | 2.47 | 6.10E-07 | 1.76 | 1.86E-04 | -1.40 | 1.14E-02 | 1.12 | 3.71E-01 | -1.05 | 6.98E-01 | -1.17 | 2.06E-01 |
|  | *LIPT1* | 1.59 | 2.45E-03 | 1.88 | 1.33E-04 | 1.19 | 2.05E-01 | 1.26 | 9.89E-02 | -1.11 | 4.27E-01 | -1.40 | 1.99E-02 |
|  | *LPCAT2* | -1.57 | 1.20E-06 | -1.85 | 1.32E-08 | -1.18 | 1.76E-02 | 1.49 | 5.56E-06 | -1.47 | 8.89E-06 | -2.19 | 2.73E-10 |
|  | *LRP5* | -3.46 | 1.08E-06 | -3.58 | 7.36E-07 | -1.04 | 8.40E-01 | -1.01 | 9.77E-01 | -1.06 | 7.34E-01 | -1.06 | 7.55E-01 |
|  | *LYPLA1* | 2.18 | 1.77E-03 | 3.27 | 2.78E-05 | 1.50 | 7.38E-02 | 1.34 | 1.84E-01 | -1.03 | 8.75E-01 | -1.39 | 1.40E-01 |
|  | *MBTPS1* | -1.43 | 2.10E-02 | -1.67 | 2.00E-03 | -1.17 | 2.94E-01 | 1.17 | 2.70E-01 | -1.07 | 6.43E-01 | -1.26 | 1.25E-01 |
|  | *MGLL* | 1.92 | 3.95E-06 | 1.42 | 2.53E-03 | -1.35 | 7.42E-03 | 1.54 | 4.47E-04 | 1.36 | 6.72E-03 | -1.13 | 2.37E-01 |
|  | *NMT2* | -1.05 | 6.95E-01 | -1.30 | 4.87E-02 | -1.24 | 1.03E-01 | 1.25 | 8.74E-02 | 1.38 | 1.83E-02 | 1.10 | 4.42E-01 |
|  | *NPC1* | 2.95 | 1.55E-06 | 2.62 | 6.91E-06 | -1.12 | 4.57E-01 | -1.00 | 9.81E-01 | 1.39 | 4.68E-02 | 1.40 | 4.46E-02 |
|  | *PHYH* | 2.05 | 1.43E-04 | 2.57 | 5.70E-06 | 1.26 | 1.42E-01 | 1.27 | 1.28E-01 | -1.02 | 8.98E-01 | -1.29 | 1.02E-01 |
|  | *PIGP* | 1.84 | 1.81E-04 | 2.10 | 1.96E-05 | 1.14 | 3.13E-01 | 1.32 | 4.39E-02 | -1.19 | 1.99E-01 | -1.57 | 2.55E-03 |
|  | *PIGW* | 1.49 | 1.64E-02 | 1.41 | 3.67E-02 | -1.06 | 7.01E-01 | -1.06 | 7.03E-01 | -1.45 | 2.56E-02 | -1.36 | 5.57E-02 |
|  | *PIK3C2B* | -1.41 | 1.02E-03 | -1.27 | 1.41E-02 | 1.11 | 2.48E-01 | -1.95 | 4.58E-07 | -2.37 | 1.08E-08 | -1.21 | 3.94E-02 |
|  | *PIP4K2A* | 1.93 | 6.09E-05 | 2.29 | 3.96E-06 | 1.18 | 2.02E-01 | 1.01 | 9.39E-01 | -1.03 | 8.36E-01 | -1.04 | 7.76E-01 |
|  | *PLA2G4C* | 1.89 | 7.22E-06 | 2.17 | 5.54E-07 | 1.15 | 2.00E-01 | 1.20 | 8.98E-02 | 1.24 | 5.23E-02 | 1.03 | 7.79E-01 |
|  | *PLAUR* | -1.03 | 8.26E-01 | -1.05 | 7.03E-01 | -1.02 | 8.71E-01 | 1.37 | 1.66E-02 | 1.87 | 4.75E-05 | 1.37 | 1.56E-02 |
|  | *PLD6* | 1.76 | 2.82E-04 | 1.82 | 1.53E-04 | 1.04 | 7.84E-01 | 1.14 | 3.08E-01 | 1.18 | 2.01E-01 | 1.04 | 7.83E-01 |
|  | *PLTP* | -1.82 | 7.10E-05 | -2.10 | 5.48E-06 | -1.15 | 2.34E-01 | 1.08 | 5.24E-01 | 1.30 | 3.51E-02 | 1.21 | 1.21E-01 |
|  | *PPAP2A* | 1.84 | 5.96E-03 | 1.98 | 2.58E-03 | 1.08 | 7.09E-01 | -1.07 | 7.24E-01 | -1.64 | 2.07E-02 | -1.53 | 4.31E-02 |
|  | *PTGES* | 1.25 | 8.53E-02 | 2.17 | 5.05E-06 | 1.74 | 2.35E-04 | 1.19 | 1.68E-01 | 1.39 | 1.40E-02 | 1.17 | 2.16E-01 |
|  | *PTGES2* | 1.27 | 7.50E-02 | 1.35 | 2.96E-02 | 1.06 | 6.42E-01 | -1.45 | 8.42E-03 | -1.39 | 1.83E-02 | 1.05 | 7.20E-01 |
|  | *PTGR1* | 1.81 | 3.38E-05 | 2.04 | 3.47E-06 | 1.13 | 2.80E-01 | 1.18 | 1.51E-01 | -1.40 | 6.22E-03 | -1.64 | 2.25E-04 |
|  | *RBM12* | -1.31 | 1.46E-02 | -1.37 | 4.73E-03 | -1.05 | 6.10E-01 | -1.02 | 8.73E-01 | 1.26 | 3.09E-02 | 1.28 | 2.22E-02 |
|  | *SC4MOL* | 3.04 | 7.17E-05 | 2.53 | 4.61E-04 | -1.20 | 4.06E-01 | 1.28 | 2.66E-01 | -1.11 | 6.28E-01 | -1.43 | 1.18E-01 |
|  | *SEC14L2* | -1.94 | 9.37E-06 | -1.77 | 5.50E-05 | 1.10 | 4.08E-01 | -1.18 | 1.41E-01 | 1.31 | 2.48E-02 | 1.54 | 8.63E-04 |
|  | *SLC27A1* | -1.65 | 6.04E-04 | -1.41 | 1.12E-02 | 1.17 | 2.02E-01 | -1.08 | 5.51E-01 | 1.02 | 8.99E-01 | 1.09 | 4.71E-01 |
|  | *SPTLC1* | 1.69 | 3.73E-04 | 2.04 | 1.28E-05 | 1.21 | 1.33E-01 | 1.16 | 2.24E-01 | -1.27 | 6.32E-02 | -1.48 | 4.54E-03 |
|  | *ST6GALNAC6* | -2.24 | 3.68E-05 | -2.39 | 1.47E-05 | -1.07 | 6.66E-01 | -1.13 | 4.32E-01 | 1.13 | 4.28E-01 | 1.27 | 1.24E-01 |
|  | *STK11* | -1.82 | 5.17E-05 | -2.26 | 1.09E-06 | -1.24 | 7.16E-02 | -1.41 | 6.73E-03 | -1.17 | 1.78E-01 | 1.21 | 1.15E-01 |
|  | *SULT1A1* | -2.49 | 6.80E-03 | -2.15 | 1.95E-02 | 1.16 | 6.28E-01 | -1.87 | 5.08E-02 | -1.09 | 7.67E-01 | 1.71 | 9.00E-02 |
|  | *TPI1* | -2.06 | 1.88E-03 | -2.11 | 1.42E-03 | -1.03 | 9.00E-01 | -1.20 | 3.67E-01 | -1.21 | 3.47E-01 | -1.01 | 9.68E-01 |
|  |  |  |  |  |  |  |  |  |  |  |  |  |  |
| **Signal transduction** | *ACCN2* | -2.14 | 1.47E-05 | -2.14 | 1.48E-05 | 1.00 | 9.98E-01 | -1.14 | 3.21E-01 | -1.18 | 2.07E-01 | -1.04 | 7.76E-01 |
|  | *ADAMTS1* | 1.01 | 9.70E-01 | 1.23 | 1.51E-01 | 1.22 | 1.61E-01 | -2.25 | 1.25E-05 | -1.16 | 2.85E-01 | 1.94 | 1.29E-04 |
|  | *AGAP8* | -1.64 | 5.98E-04 | -1.59 | 1.07E-03 | 1.03 | 7.95E-01 | -1.25 | 7.60E-02 | 1.16 | 2.22E-01 | 1.46 | 5.55E-03 |
|  | *AGRN* | -2.20 | 1.99E-05 | -2.25 | 1.50E-05 | -1.02 | 8.93E-01 | -1.26 | 1.11E-01 | -1.06 | 6.63E-01 | 1.19 | 2.33E-01 |
|  | *AKAP12* | 1.30 | 4.89E-02 | 1.84 | 1.25E-04 | 1.41 | 1.32E-02 | 1.60 | 1.43E-03 | 1.54 | 2.74E-03 | -1.04 | 7.74E-01 |
|  | *ANKDD1A* | -1.28 | 2.80E-02 | -1.19 | 1.07E-01 | 1.07 | 4.97E-01 | 1.00 | 9.88E-01 | 1.47 | 1.51E-03 | 1.47 | 1.57E-03 |
|  | *ANXA3* | 1.73 | 2.37E-05 | 1.36 | 5.28E-03 | -1.27 | 2.37E-02 | 1.60 | 1.28E-04 | 1.03 | 7.63E-01 | -1.55 | 2.50E-04 |
|  | *APBB3* | -1.42 | 6.12E-02 | -1.13 | 5.02E-01 | 1.26 | 2.06E-01 | 1.02 | 9.28E-01 | 1.18 | 3.56E-01 | 1.16 | 4.03E-01 |
|  | *APLN* | -1.09 | 5.53E-01 | -2.02 | 7.27E-05 | -1.86 | 2.72E-04 | 1.12 | 4.08E-01 | -1.61 | 2.87E-03 | -1.80 | 4.36E-04 |
|  | *ARL5B* | 1.58 | 1.10E-04 | 1.75 | 1.09E-05 | 1.11 | 2.89E-01 | 1.16 | 1.37E-01 | 1.28 | 1.65E-02 | 1.11 | 2.92E-01 |
|  | *ASAP1* | 2.21 | 1.20E-06 | 2.44 | 2.23E-07 | 1.11 | 3.71E-01 | 1.28 | 4.00E-02 | 1.04 | 7.41E-01 | -1.23 | 7.66E-02 |
|  | *ASAP3* | -4.01 | 1.80E-08 | -3.92 | 2.28E-08 | 1.02 | 8.83E-01 | -1.10 | 5.26E-01 | -1.20 | 2.19E-01 | -1.10 | 5.38E-01 |
|  | *ASB9* | -1.65 | 6.60E-04 | -1.84 | 9.31E-05 | -1.11 | 3.85E-01 | -1.31 | 3.95E-02 | -1.35 | 2.28E-02 | -1.03 | 7.90E-01 |
|  | *AXL* | -1.08 | 3.94E-01 | -1.69 | 1.30E-05 | -1.56 | 8.19E-05 | 1.48 | 3.07E-04 | 1.35 | 3.33E-03 | -1.10 | 2.98E-01 |
|  | *BAMBI* | -2.01 | 2.72E-05 | -2.63 | 3.99E-07 | -1.31 | 4.50E-02 | 1.01 | 9.48E-01 | -2.33 | 2.45E-06 | -2.35 | 2.15E-06 |
|  | *BMPR2* | -1.64 | 6.10E-05 | -2.11 | 3.25E-07 | -1.29 | 1.64E-02 | -1.02 | 8.26E-01 | -1.31 | 1.04E-02 | -1.29 | 1.68E-02 |
|  | *C5orf13* | -1.58 | 1.30E-06 | -1.42 | 3.29E-05 | 1.11 | 1.26E-01 | 1.26 | 2.28E-03 | 1.01 | 9.30E-01 | -1.25 | 2.78E-03 |
|  | *CACNB3* | -1.73 | 2.79E-05 | -1.49 | 8.31E-04 | 1.17 | 1.36E-01 | 1.14 | 2.13E-01 | 1.31 | 1.30E-02 | 1.16 | 1.60E-01 |
|  | *CAPN5* | -2.21 | 5.79E-08 | -1.99 | 4.32E-07 | 1.11 | 2.65E-01 | -1.17 | 1.00E-01 | -1.03 | 7.63E-01 | 1.14 | 1.71E-01 |
|  | *CBS* | 3.63 | 2.07E-10 | 4.05 | 5.41E-11 | 1.12 | 2.94E-01 | 1.45 | 1.68E-03 | 1.92 | 5.00E-06 | 1.32 | 1.42E-02 |
|  | *CCL14* | -1.62 | 2.77E-05 | -1.66 | 1.56E-05 | -1.02 | 7.87E-01 | -1.90 | 7.63E-07 | -2.02 | 2.07E-07 | -1.06 | 4.82E-01 |
|  | *CCNA2* | 1.50 | 7.01E-03 | 1.02 | 8.75E-01 | -1.47 | 9.91E-03 | 1.22 | 1.62E-01 | 1.04 | 7.60E-01 | -1.17 | 2.65E-01 |
|  | *CCNE1* | 1.32 | 5.53E-02 | 1.71 | 9.82E-04 | 1.29 | 7.63E-02 | -1.04 | 7.96E-01 | -1.11 | 4.75E-01 | -1.07 | 6.46E-01 |
|  | *CCRL2* | 1.82 | 4.40E-05 | 1.91 | 1.76E-05 | 1.05 | 6.68E-01 | -1.02 | 8.60E-01 | -1.20 | 1.21E-01 | -1.18 | 1.64E-01 |
|  | *CD59* | -1.65 | 1.26E-04 | -2.49 | 6.00E-08 | -1.51 | 9.26E-04 | -1.16 | 1.64E-01 | -1.13 | 2.65E-01 | 1.03 | 7.67E-01 |
|  | *CDC42SE2* | 1.98 | 2.08E-05 | 2.30 | 1.63E-06 | 1.16 | 2.20E-01 | 1.08 | 5.25E-01 | 1.01 | 9.45E-01 | -1.07 | 5.70E-01 |
|  | *CHMP5* | 1.87 | 3.92E-03 | 1.88 | 3.61E-03 | 1.01 | 9.71E-01 | 1.42 | 7.75E-02 | -1.05 | 7.94E-01 | -1.50 | 4.65E-02 |
|  | *CLIC3* | -2.64 | 2.92E-07 | -2.28 | 2.75E-06 | 1.16 | 2.46E-01 | 1.88 | 7.13E-05 | 1.55 | 2.12E-03 | -1.21 | 1.42E-01 |
|  | *CNIH* | 2.73 | 7.52E-04 | 3.17 | 1.96E-04 | 1.16 | 5.52E-01 | 1.67 | 5.24E-02 | -1.09 | 7.23E-01 | -1.83 | 2.55E-02 |
|  | *COLEC12* | -1.46 | 1.52E-02 | -1.62 | 3.18E-03 | -1.11 | 4.82E-01 | -1.21 | 1.89E-01 | -2.20 | 2.75E-05 | -1.81 | 5.30E-04 |
|  | *CORO2A* | -1.22 | 3.62E-02 | -1.09 | 3.48E-01 | 1.12 | 2.10E-01 | 1.63 | 2.41E-05 | 1.61 | 3.33E-05 | -1.01 | 8.79E-01 |
|  | *CSNK1E* | -1.20 | 1.90E-02 | -1.21 | 1.73E-02 | -1.00 | 9.64E-01 | 1.04 | 5.62E-01 | 1.32 | 1.13E-03 | 1.27 | 4.18E-03 |
|  | *CSNK2A1* | 2.30 | 2.72E-05 | 2.47 | 9.96E-06 | 1.08 | 6.33E-01 | 1.34 | 6.68E-02 | 1.02 | 8.94E-01 | -1.31 | 8.62E-02 |
|  | *CXCL2* | 1.70 | 7.39E-03 | 2.76 | 1.84E-05 | 1.62 | 1.33E-02 | 1.11 | 5.56E-01 | 2.27 | 1.99E-04 | 2.04 | 7.50E-04 |
|  | *DAPP1* | -1.94 | 1.27E-03 | -1.86 | 2.20E-03 | 1.04 | 8.08E-01 | -1.39 | 7.57E-02 | 1.10 | 5.86E-01 | 1.53 | 2.53E-02 |
|  | *DCLK2* | -1.56 | 1.96E-03 | -1.60 | 1.20E-03 | -1.03 | 8.27E-01 | -1.07 | 5.87E-01 | 1.48 | 5.13E-03 | 1.58 | 1.50E-03 |
|  | *DEF8* | 1.66 | 1.22E-07 | 1.64 | 1.71E-07 | -1.01 | 8.48E-01 | -1.02 | 7.99E-01 | 1.03 | 6.07E-01 | 1.05 | 4.45E-01 |
|  | *DGKA* | -1.51 | 3.05E-03 | -2.10 | 9.01E-06 | -1.39 | 1.50E-02 | -1.15 | 2.54E-01 | 1.27 | 6.41E-02 | 1.47 | 5.54E-03 |
|  | *DKK3* | -1.58 | 7.96E-04 | -1.83 | 4.90E-05 | -1.16 | 2.19E-01 | 1.07 | 5.42E-01 | -1.37 | 1.32E-02 | -1.47 | 3.39E-03 |
|  | *DLG4* | -2.60 | 2.23E-04 | -2.34 | 6.88E-04 | 1.11 | 6.17E-01 | -1.12 | 5.92E-01 | 1.11 | 6.32E-01 | 1.24 | 3.16E-01 |
|  | *EFNA1* | -1.85 | 1.87E-08 | -1.91 | 8.54E-09 | -1.03 | 6.22E-01 | 1.26 | 2.32E-03 | -1.13 | 7.25E-02 | -1.42 | 3.53E-05 |
|  | *FAM13A* | -3.77 | 6.61E-10 | -3.24 | 4.61E-09 | 1.17 | 1.89E-01 | -1.33 | 2.15E-02 | 1.02 | 8.94E-01 | 1.35 | 1.61E-02 |
|  | *FCN3* | -2.20 | 2.60E-06 | -2.21 | 2.34E-06 | -1.01 | 9.57E-01 | -2.14 | 4.08E-06 | -2.47 | 4.04E-07 | -1.15 | 2.39E-01 |
|  | *FOS* | -1.17 | 6.15E-01 | -1.18 | 5.99E-01 | -1.01 | 9.82E-01 | -3.32 | 1.22E-03 | 1.04 | 9.07E-01 | 3.44 | 9.38E-04 |
|  | *FSTL1* | -1.57 | 2.11E-04 | -1.81 | 9.18E-06 | -1.15 | 1.57E-01 | 1.35 | 6.57E-03 | 1.10 | 3.57E-01 | -1.23 | 4.75E-02 |
|  | *FZD8* | 1.62 | 2.78E-04 | 2.04 | 2.94E-06 | 1.26 | 4.35E-02 | 1.18 | 1.33E-01 | 1.26 | 4.24E-02 | 1.07 | 5.48E-01 |
|  | *G3BP1* | 1.47 | 1.99E-02 | 1.70 | 2.27E-03 | 1.16 | 3.32E-01 | 1.13 | 4.39E-01 | -1.19 | 2.62E-01 | -1.34 | 6.70E-02 |
|  | *G3BP2* | 2.29 | 3.22E-04 | 2.63 | 6.65E-05 | 1.15 | 4.79E-01 | 1.34 | 1.32E-01 | -1.25 | 2.46E-01 | -1.68 | 1.24E-02 |
|  | *GABBR1* | -1.89 | 3.41E-03 | -1.52 | 4.07E-02 | 1.25 | 2.60E-01 | -1.52 | 3.98E-02 | 1.08 | 6.99E-01 | 1.64 | 1.78E-02 |
|  | *GAS6* | -1.93 | 5.31E-06 | -2.27 | 2.69E-07 | -1.18 | 1.32E-01 | -1.25 | 4.67E-02 | -1.14 | 2.20E-01 | 1.09 | 3.99E-01 |
|  | *GDF15* | 2.74 | 1.71E-05 | 3.51 | 1.02E-06 | 1.28 | 1.72E-01 | -1.03 | 8.82E-01 | 1.24 | 2.41E-01 | 1.27 | 1.89E-01 |
|  | *GDI1* | -1.31 | 1.46E-01 | -1.31 | 1.45E-01 | -1.00 | 9.99E-01 | -1.05 | 8.04E-01 | 1.55 | 2.34E-02 | 1.62 | 1.38E-02 |
|  | *GEM* | 1.75 | 2.00E-04 | 1.92 | 3.85E-05 | 1.10 | 4.55E-01 | 1.27 | 6.12E-02 | 1.12 | 3.72E-01 | -1.14 | 2.93E-01 |
|  | *GFRA2* | -1.29 | 1.56E-03 | -1.23 | 6.40E-03 | 1.04 | 5.31E-01 | 1.06 | 4.40E-01 | 1.54 | 5.69E-06 | 1.46 | 2.89E-05 |
|  | *GNA11* | -1.80 | 2.05E-04 | -2.01 | 3.08E-05 | -1.12 | 3.91E-01 | -1.07 | 6.04E-01 | 1.18 | 2.05E-01 | 1.26 | 8.21E-02 |
|  | *GNG10* | 3.60 | 1.88E-04 | 4.58 | 2.88E-05 | 1.27 | 3.95E-01 | 1.41 | 2.26E-01 | -1.03 | 9.29E-01 | -1.45 | 1.96E-01 |
|  | *GNG12* | 2.67 | 4.20E-04 | 3.41 | 4.14E-05 | 1.27 | 3.01E-01 | 1.32 | 2.44E-01 | -1.16 | 5.12E-01 | -1.53 | 7.72E-02 |
|  | *GPR1* | -2.89 | 4.86E-03 | -2.97 | 4.06E-03 | -1.03 | 9.35E-01 | -1.87 | 7.38E-02 | 1.05 | 8.80E-01 | 1.97 | 5.51E-02 |
|  | *GPR126* | -2.10 | 4.70E-06 | -2.57 | 1.73E-07 | -1.22 | 9.50E-02 | 1.33 | 2.50E-02 | -1.04 | 7.65E-01 | -1.37 | 1.32E-02 |
|  | *GPR162* | -3.19 | 1.48E-06 | -2.30 | 8.48E-05 | 1.39 | 6.28E-02 | -1.25 | 1.92E-01 | -1.34 | 8.96E-02 | -1.08 | 6.66E-01 |
|  | *GPR89C* | 2.06 | 2.74E-07 | 1.72 | 1.22E-05 | -1.20 | 6.35E-02 | 1.22 | 4.42E-02 | -1.12 | 2.42E-01 | -1.36 | 3.39E-03 |
|  | *GRIN3B* | -1.59 | 3.47E-04 | -1.39 | 5.44E-03 | 1.14 | 2.31E-01 | 1.53 | 7.44E-04 | 1.31 | 1.87E-02 | -1.17 | 1.59E-01 |
|  | *GTF2H2B* | 1.89 | 5.54E-05 | 2.14 | 6.77E-06 | 1.13 | 3.26E-01 | 1.23 | 1.12E-01 | -1.18 | 1.82E-01 | -1.45 | 6.74E-03 |
|  | *HTRA1* | -3.35 | 1.81E-09 | -3.25 | 2.67E-09 | 1.03 | 7.89E-01 | 1.18 | 1.41E-01 | -1.10 | 3.73E-01 | -1.31 | 2.46E-02 |
|  | *IFIT1* | -4.15 | 1.46E-05 | -1.97 | 1.17E-02 | 2.10 | 6.66E-03 | -2.15 | 5.45E-03 | -1.78 | 2.93E-02 | 1.21 | 4.40E-01 |
|  | *IGFBP4* | -2.93 | 1.65E-07 | -3.33 | 3.13E-08 | -1.14 | 3.38E-01 | -1.07 | 5.89E-01 | 1.13 | 3.66E-01 | 1.21 | 1.57E-01 |
|  | *IGFBP6* | 1.10 | 2.82E-01 | -1.15 | 1.20E-01 | -1.27 | 1.34E-02 | 1.22 | 3.51E-02 | 1.69 | 1.24E-05 | 1.38 | 1.73E-03 |
|  | *IL20RB* | 1.72 | 2.44E-05 | 1.42 | 1.95E-03 | -1.21 | 6.04E-02 | -1.11 | 2.79E-01 | -1.20 | 7.25E-02 | -1.08 | 4.39E-01 |
|  | *INPP4B* | 2.02 | 4.85E-07 | 1.64 | 4.18E-05 | -1.23 | 3.72E-02 | 1.50 | 3.43E-04 | 1.55 | 1.52E-04 | 1.03 | 7.14E-01 |
|  | *ITPR2* | -1.60 | 1.64E-02 | -1.58 | 2.00E-02 | 1.02 | 9.25E-01 | -1.07 | 6.93E-01 | -1.57 | 2.04E-02 | -1.47 | 4.62E-02 |
|  | *KCNH3* | -1.81 | 1.32E-06 | -1.87 | 6.68E-07 | -1.03 | 7.22E-01 | 1.19 | 5.38E-02 | 1.28 | 7.97E-03 | 1.08 | 3.70E-01 |
|  | *KCNH6* | -2.38 | 2.15E-03 | -2.39 | 2.10E-03 | -1.00 | 9.92E-01 | -1.67 | 4.97E-02 | -1.12 | 6.41E-01 | 1.48 | 1.21E-01 |
|  | *LAT2* | -2.27 | 1.14E-06 | -2.74 | 6.20E-08 | -1.21 | 1.20E-01 | -1.69 | 2.39E-04 | -1.26 | 6.10E-02 | 1.34 | 1.93E-02 |
|  | *LILRB3* | -2.04 | 1.81E-04 | -1.84 | 8.17E-04 | 1.11 | 5.05E-01 | -1.43 | 2.90E-02 | 1.01 | 9.37E-01 | 1.45 | 2.46E-02 |
|  | *LOC653631* | 2.11 | 1.28E-03 | 2.16 | 9.55E-04 | 1.03 | 8.98E-01 | 1.27 | 2.42E-01 | -1.08 | 7.14E-01 | -1.36 | 1.31E-01 |
|  | *LPAR1* | 1.84 | 2.81E-07 | 2.06 | 2.21E-08 | 1.12 | 1.51E-01 | 1.88 | 1.64E-07 | 1.88 | 1.69E-07 | -1.00 | 9.86E-01 |
|  | *LTBP2* | -2.47 | 8.26E-07 | -2.53 | 6.02E-07 | -1.02 | 8.67E-01 | -1.08 | 5.38E-01 | -1.15 | 2.71E-01 | -1.06 | 6.18E-01 |
|  | *LTBP3* | -1.74 | 3.68E-04 | -1.91 | 7.76E-05 | -1.09 | 4.86E-01 | -1.10 | 4.78E-01 | -1.11 | 4.06E-01 | -1.02 | 9.01E-01 |
|  | *LTBP4* | -1.96 | 3.08E-06 | -2.28 | 1.90E-07 | -1.16 | 1.52E-01 | 1.12 | 2.81E-01 | 1.48 | 1.10E-03 | 1.32 | 1.26E-02 |
|  | *MAP4K5* | -1.26 | 6.23E-02 | -1.47 | 4.15E-03 | -1.17 | 2.12E-01 | -1.07 | 5.60E-01 | 1.30 | 4.00E-02 | 1.39 | 1.16E-02 |
|  | *MAPK11* | -1.86 | 2.42E-03 | -2.15 | 3.85E-04 | -1.16 | 4.20E-01 | -1.76 | 4.66E-03 | -1.23 | 2.58E-01 | 1.44 | 5.40E-02 |
|  | *MAPK6* | 1.95 | 1.65E-05 | 2.18 | 2.22E-06 | 1.12 | 3.31E-01 | 1.31 | 3.01E-02 | 1.31 | 3.06E-02 | -1.00 | 9.93E-01 |
|  | *MAPK8IP3* | -1.58 | 2.80E-02 | -1.41 | 9.16E-02 | 1.12 | 5.51E-01 | -1.44 | 7.46E-02 | 1.10 | 6.31E-01 | 1.58 | 2.85E-02 |
|  | *MIB2* | -1.62 | 3.57E-04 | -1.65 | 2.53E-04 | -1.02 | 8.78E-01 | -1.19 | 1.37E-01 | 1.22 | 8.69E-02 | 1.45 | 3.44E-03 |
|  | *MTA1* | -1.53 | 1.25E-03 | -1.46 | 3.06E-03 | 1.05 | 6.91E-01 | -1.02 | 8.60E-01 | 1.34 | 1.77E-02 | 1.36 | 1.22E-02 |
|  | *NAMPT* | 1.68 | 3.24E-03 | 2.55 | 8.73E-06 | 1.52 | 1.37E-02 | -1.01 | 9.62E-01 | 1.07 | 6.84E-01 | 1.07 | 6.49E-01 |
|  | *NFAT5* | -2.63 | 1.71E-05 | -2.68 | 1.33E-05 | -1.02 | 9.06E-01 | -1.31 | 1.20E-01 | -1.03 | 8.76E-01 | 1.28 | 1.58E-01 |
|  | *NR2E3* | -1.97 | 6.34E-05 | -1.88 | 1.33E-04 | 1.05 | 7.35E-01 | -1.57 | 2.77E-03 | -1.04 | 7.63E-01 | 1.51 | 5.44E-03 |
|  | *NR2F1* | -2.62 | 1.85E-07 | -3.23 | 1.01E-08 | -1.23 | 9.53E-02 | 1.27 | 6.10E-02 | 1.49 | 3.46E-03 | 1.18 | 1.89E-01 |
|  | *NRGN* | -1.44 | 2.34E-02 | -1.56 | 7.56E-03 | -1.08 | 6.02E-01 | -1.64 | 3.58E-03 | 1.19 | 2.59E-01 | 1.95 | 2.68E-04 |
|  | *NTN4* | -1.10 | 6.73E-01 | -1.39 | 1.64E-01 | -1.26 | 3.20E-01 | 1.49 | 9.45E-02 | 1.02 | 9.21E-01 | -1.45 | 1.13E-01 |
|  | *OPRL1* | -2.45 | 7.77E-07 | -2.25 | 3.06E-06 | 1.09 | 4.83E-01 | 1.26 | 7.01E-02 | 1.32 | 3.42E-02 | 1.05 | 7.19E-01 |
|  | *P2RY2* | 1.41 | 4.61E-04 | 1.10 | 2.46E-01 | -1.28 | 6.58E-03 | -1.21 | 2.71E-02 | -1.14 | 1.23E-01 | 1.07 | 4.42E-01 |
|  | *PCNA* | 1.74 | 1.43E-03 | 1.57 | 6.81E-03 | -1.11 | 4.90E-01 | 1.06 | 7.04E-01 | -1.10 | 5.21E-01 | -1.17 | 3.12E-01 |
|  | *PDE4C* | -2.04 | 2.81E-03 | -2.04 | 2.76E-03 | -1.00 | 9.93E-01 | -1.50 | 6.52E-02 | 1.10 | 6.42E-01 | 1.65 | 2.55E-02 |
|  | *PIAS2* | 1.70 | 5.23E-07 | 1.99 | 1.23E-08 | 1.17 | 4.00E-02 | 1.14 | 8.80E-02 | 1.01 | 9.02E-01 | -1.13 | 1.10E-01 |
|  | *PILRB* | -1.97 | 2.00E-03 | -1.88 | 3.31E-03 | 1.04 | 8.23E-01 | -1.64 | 1.70E-02 | -1.28 | 2.02E-01 | 1.28 | 2.08E-01 |
|  | *PKIG* | 1.69 | 2.83E-04 | 1.91 | 3.17E-05 | 1.13 | 3.25E-01 | -1.35 | 1.86E-02 | -1.06 | 6.21E-01 | 1.28 | 5.17E-02 |
|  | *PKN1* | -1.54 | 5.22E-03 | -1.77 | 5.45E-04 | -1.15 | 3.22E-01 | -1.09 | 5.52E-01 | 1.33 | 4.88E-02 | 1.45 | 1.41E-02 |
|  | *PKN3* | -1.97 | 9.41E-06 | -2.44 | 2.55E-07 | -1.23 | 7.52E-02 | -1.60 | 5.02E-04 | -1.13 | 3.02E-01 | 1.42 | 5.32E-03 |
|  | *PLEKHA1* | -2.17 | 4.53E-07 | -2.42 | 6.84E-08 | -1.12 | 2.96E-01 | 1.07 | 5.32E-01 | 1.76 | 2.68E-05 | 1.65 | 1.04E-04 |
|  | *PLEKHM1* | 1.58 | 9.22E-07 | 1.52 | 3.06E-06 | -1.04 | 5.40E-01 | -1.17 | 2.14E-02 | -1.08 | 2.49E-01 | 1.09 | 2.01E-01 |
|  | *PLXNA2* | -1.87 | 3.81E-05 | -2.53 | 2.19E-07 | -1.36 | 1.64E-02 | 1.12 | 3.42E-01 | 1.21 | 1.11E-01 | 1.08 | 4.92E-01 |
|  | *PLXNA3* | -1.62 | 2.08E-03 | -1.47 | 9.88E-03 | 1.10 | 4.87E-01 | -1.08 | 5.59E-01 | 1.21 | 1.69E-01 | 1.31 | 5.75E-02 |
|  | *PPIC* | 1.30 | 1.93E-02 | 1.45 | 1.92E-03 | 1.11 | 3.04E-01 | 1.02 | 8.29E-01 | -1.44 | 2.23E-03 | -1.47 | 1.37E-03 |
|  | *PPM1A* | 1.76 | 1.02E-03 | 2.15 | 5.14E-05 | 1.22 | 1.89E-01 | -1.05 | 7.28E-01 | -1.04 | 8.02E-01 | 1.01 | 9.23E-01 |
|  | *PPP1R16B* | -1.75 | 9.92E-03 | -1.96 | 2.78E-03 | -1.12 | 5.69E-01 | -2.53 | 1.54E-04 | -3.49 | 4.80E-06 | -1.38 | 1.15E-01 |
|  | *PPP2R2B* | -2.47 | 2.75E-05 | -3.04 | 2.11E-06 | -1.23 | 2.20E-01 | -1.41 | 4.93E-02 | -1.77 | 2.55E-03 | -1.25 | 1.81E-01 |
|  | *PTPLA* | 1.32 | 3.83E-02 | 1.50 | 4.08E-03 | 1.14 | 3.06E-01 | 1.24 | 9.88E-02 | -1.40 | 1.41E-02 | -1.73 | 3.04E-04 |
|  | *PTPRE* | -1.95 | 1.09E-06 | -1.96 | 9.73E-07 | -1.01 | 9.52E-01 | -1.13 | 2.16E-01 | -1.20 | 6.37E-02 | -1.07 | 4.96E-01 |
|  | *RAB12* | -1.47 | 1.41E-02 | -1.26 | 1.23E-01 | 1.17 | 2.86E-01 | -1.22 | 1.85E-01 | 1.15 | 3.42E-01 | 1.40 | 3.01E-02 |
|  | *RAB23* | 2.07 | 1.48E-05 | 1.98 | 2.99E-05 | -1.04 | 7.39E-01 | 1.29 | 5.16E-02 | 1.08 | 5.59E-01 | -1.20 | 1.54E-01 |
|  | *RAB32* | 2.93 | 1.80E-08 | 3.32 | 3.27E-09 | 1.13 | 2.80E-01 | 1.21 | 1.04E-01 | -1.07 | 5.48E-01 | -1.30 | 3.20E-02 |
|  | *RAB40B* | -1.99 | 5.29E-06 | -2.03 | 3.75E-06 | -1.02 | 8.64E-01 | 1.05 | 6.47E-01 | 1.08 | 4.65E-01 | 1.03 | 7.82E-01 |
|  | *RAB7L1* | 2.09 | 6.04E-06 | 1.95 | 1.95E-05 | -1.07 | 5.74E-01 | 1.07 | 5.43E-01 | -1.11 | 3.71E-01 | -1.20 | 1.42E-01 |
|  | *RABL3* | 1.76 | 3.86E-06 | 2.13 | 6.62E-08 | 1.21 | 3.93E-02 | 1.21 | 3.78E-02 | -1.07 | 4.25E-01 | -1.30 | 6.77E-03 |
|  | *RASGRP3* | 1.13 | 2.69E-01 | -1.02 | 8.84E-01 | -1.15 | 2.14E-01 | -2.13 | 1.93E-06 | -2.23 | 8.57E-07 | -1.05 | 6.74E-01 |
|  | *RASIP1* | -2.76 | 2.49E-06 | -3.75 | 6.09E-08 | -1.36 | 5.52E-02 | -2.14 | 7.78E-05 | -3.06 | 6.56E-07 | -1.43 | 2.88E-02 |
|  | *RASL10A* | -1.86 | 2.60E-11 | -1.81 | 5.86E-11 | 1.03 | 5.00E-01 | 1.01 | 8.60E-01 | 1.07 | 1.31E-01 | 1.06 | 1.77E-01 |
|  | *RASSF2* | -1.89 | 4.42E-04 | -1.74 | 1.52E-03 | 1.09 | 5.85E-01 | -2.17 | 5.94E-05 | -1.96 | 2.73E-04 | 1.11 | 4.92E-01 |
|  | *RGS17* | 1.02 | 8.75E-01 | 1.66 | 7.66E-04 | 1.63 | 1.09E-03 | -1.01 | 9.32E-01 | 1.65 | 8.11E-04 | 1.67 | 6.70E-04 |
|  | *RGS4* | 2.34 | 3.61E-05 | 2.31 | 4.31E-05 | -1.01 | 9.35E-01 | 1.39 | 4.98E-02 | 1.43 | 3.36E-02 | 1.03 | 8.46E-01 |
|  | *RPS6KA2* | -2.63 | 7.37E-09 | -2.22 | 1.35E-07 | 1.19 | 9.11E-02 | -1.31 | 1.10E-02 | -1.91 | 2.51E-06 | -1.45 | 1.01E-03 |
|  | *RPS6KA3* | 1.88 | 5.21E-04 | 2.69 | 3.30E-06 | 1.43 | 2.76E-02 | 1.14 | 4.06E-01 | -1.25 | 1.53E-01 | -1.42 | 3.08E-02 |
|  | *RPS6KA5* | -1.38 | 1.37E-03 | -1.03 | 7.19E-01 | 1.34 | 3.09E-03 | 1.37 | 1.60E-03 | 1.33 | 3.57E-03 | -1.03 | 7.22E-01 |
|  | *RQCD1* | 2.15 | 4.78E-05 | 2.10 | 7.02E-05 | -1.03 | 8.60E-01 | 1.15 | 3.54E-01 | -1.05 | 7.52E-01 | -1.20 | 2.20E-01 |
|  | *RRAGB* | 2.00 | 5.08E-05 | 2.55 | 1.12E-06 | 1.28 | 7.50E-02 | 1.28 | 7.13E-02 | -1.07 | 6.22E-01 | -1.37 | 2.65E-02 |
|  | *RRBP1* | -1.60 | 7.34E-03 | -2.13 | 1.34E-04 | -1.33 | 8.68E-02 | -1.23 | 2.02E-01 | 1.18 | 2.92E-01 | 1.46 | 2.69E-02 |
|  | *SGSM2* | -1.92 | 3.39E-04 | -1.70 | 2.17E-03 | 1.13 | 4.14E-01 | -1.24 | 1.59E-01 | -1.16 | 3.19E-01 | 1.07 | 6.61E-01 |
|  | *SH2D3C* | -2.45 | 1.76E-06 | -2.17 | 1.09E-05 | 1.13 | 3.69E-01 | -1.88 | 1.19E-04 | -1.66 | 1.00E-03 | 1.13 | 3.47E-01 |
|  | *SHANK3* | -2.96 | 5.66E-06 | -3.21 | 2.25E-06 | -1.08 | 6.45E-01 | -2.84 | 9.27E-06 | -2.66 | 1.99E-05 | 1.07 | 7.16E-01 |
|  | *SIK1* | 1.10 | 2.17E-01 | 1.30 | 2.14E-03 | 1.18 | 3.35E-02 | -1.07 | 3.81E-01 | 1.75 | 4.62E-07 | 1.87 | 9.45E-08 |
|  | *SKAP2* | 1.63 | 2.61E-03 | 2.13 | 3.89E-05 | 1.31 | 7.16E-02 | 1.28 | 9.72E-02 | 1.09 | 5.31E-01 | -1.17 | 2.81E-01 |
|  | *SLC12A2* | 2.08 | 1.84E-03 | 2.40 | 3.68E-04 | 1.16 | 4.78E-01 | -1.20 | 3.84E-01 | -1.31 | 2.00E-01 | -1.09 | 6.66E-01 |
|  | *SLC7A1* | 1.58 | 2.37E-05 | 1.28 | 6.51E-03 | -1.23 | 1.95E-02 | -1.22 | 2.36E-02 | 1.33 | 2.18E-03 | 1.63 | 1.03E-05 |
|  | *SOCS5* | 1.41 | 3.09E-02 | 1.81 | 7.16E-04 | 1.29 | 1.01E-01 | -1.04 | 8.01E-01 | -1.41 | 2.89E-02 | -1.36 | 4.83E-02 |
|  | *SPARC* | -1.69 | 2.50E-04 | -1.65 | 3.72E-04 | 1.02 | 8.58E-01 | -1.46 | 3.99E-03 | -1.82 | 5.99E-05 | -1.24 | 7.30E-02 |
|  | *SPG21* | 1.55 | 2.87E-04 | 2.21 | 2.14E-07 | 1.42 | 2.08E-03 | 1.13 | 2.43E-01 | -1.09 | 4.12E-01 | -1.22 | 5.55E-02 |
|  | *SPRY2* | 1.96 | 1.73E-05 | 2.70 | 9.13E-08 | 1.38 | 1.25E-02 | -1.09 | 4.82E-01 | 2.07 | 6.37E-06 | 2.25 | 1.55E-06 |
|  | *SPRY4* | 1.14 | 2.00E-01 | 1.42 | 1.67E-03 | 1.25 | 2.97E-02 | -1.43 | 1.64E-03 | 1.58 | 1.46E-04 | 2.26 | 1.04E-07 |
|  | *STAT2* | -2.41 | 3.46E-06 | -1.92 | 1.17E-04 | 1.25 | 1.07E-01 | -1.49 | 8.24E-03 | -1.01 | 9.42E-01 | 1.47 | 9.66E-03 |
|  | *STC1* | 3.06 | 7.36E-10 | 4.24 | 1.13E-11 | 1.38 | 3.21E-03 | 1.26 | 2.44E-02 | 2.96 | 1.22E-09 | 2.34 | 5.21E-08 |
|  | *STC2* | -1.37 | 6.00E-02 | -1.31 | 1.06E-01 | 1.05 | 7.63E-01 | 2.11 | 1.58E-04 | 2.26 | 6.18E-05 | 1.07 | 6.70E-01 |
|  | *TBC1D2* | 2.13 | 5.65E-07 | 2.14 | 5.42E-07 | 1.00 | 9.82E-01 | 1.39 | 4.07E-03 | 1.29 | 1.94E-02 | -1.08 | 4.79E-01 |
|  | *TBC1D3G* | -1.78 | 2.72E-03 | -1.53 | 1.92E-02 | 1.16 | 3.79E-01 | -1.21 | 2.64E-01 | 1.08 | 6.57E-01 | 1.31 | 1.26E-01 |
|  | *TBC1D7* | 1.47 | 8.81E-04 | 1.75 | 1.70E-05 | 1.19 | 8.49E-02 | 1.04 | 6.98E-01 | -1.20 | 7.47E-02 | -1.25 | 3.46E-02 |
|  | *TGIF1* | 1.79 | 1.16E-03 | 2.18 | 6.26E-05 | 1.22 | 2.01E-01 | 1.24 | 1.77E-01 | 1.06 | 7.14E-01 | -1.17 | 3.16E-01 |
|  | *TIMP2* | 1.96 | 6.25E-05 | 2.33 | 3.86E-06 | 1.19 | 1.94E-01 | 1.21 | 1.58E-01 | -1.03 | 8.44E-01 | -1.24 | 1.11E-01 |
|  | *TMEM189* | -1.81 | 3.90E-05 | -1.77 | 5.97E-05 | 1.02 | 8.43E-01 | 1.25 | 5.96E-02 | 1.64 | 2.60E-04 | 1.32 | 2.15E-02 |
|  | *UBE2D3* | 1.96 | 2.24E-05 | 2.04 | 1.15E-05 | 1.04 | 7.51E-01 | 1.33 | 2.85E-02 | 1.12 | 3.53E-01 | -1.19 | 1.70E-01 |
|  | *VAPA* | -1.41 | 1.63E-02 | -1.63 | 1.39E-03 | -1.16 | 2.76E-01 | -1.02 | 8.86E-01 | 1.48 | 7.95E-03 | 1.50 | 5.78E-03 |
|  | *WSB1* | -1.97 | 5.07E-04 | -2.10 | 2.02E-04 | -1.07 | 6.82E-01 | -1.47 | 2.62E-02 | -2.07 | 2.56E-04 | -1.40 | 4.87E-02 |
|  | *ZDHHC13* | 1.74 | 9.58E-04 | 2.08 | 6.13E-05 | 1.19 | 2.27E-01 | 1.05 | 7.36E-01 | -1.17 | 2.73E-01 | -1.23 | 1.58E-01 |
|  | *ZFAND6* | 2.25 | 1.56E-03 | 2.96 | 9.70E-05 | 1.32 | 2.24E-01 | 1.34 | 1.95E-01 | 1.03 | 8.99E-01 | -1.30 | 2.40E-01 |
|  | *ZFP36* | -1.00 | 9.83E-01 | 1.95 | 8.88E-04 | 1.96 | 8.46E-04 | -1.22 | 2.48E-01 | 1.55 | 1.77E-02 | 1.89 | 1.30E-03 |
|  | *ZNF259* | 1.81 | 5.80E-08 | 2.05 | 3.07E-09 | 1.13 | 7.68E-02 | 1.22 | 8.53E-03 | 1.11 | 1.31E-01 | -1.10 | 1.87E-01 |
|  |  |  |  |  |  |  |  |  |  |  |  |  |  |
| **Translation** | *ACP1* | 1.91 | 1.11E-03 | 1.87 | 1.48E-03 | -1.02 | 8.98E-01 | 1.25 | 1.94E-01 | -1.10 | 5.71E-01 | -1.38 | 6.98E-02 |
|  | *CDC25B* | 1.31 | 3.75E-03 | 1.42 | 4.41E-04 | 1.08 | 3.48E-01 | -1.64 | 9.98E-06 | -1.44 | 3.09E-04 | 1.14 | 1.25E-01 |
|  | *DENR* | -2.50 | 4.99E-03 | -2.36 | 7.80E-03 | 1.06 | 8.41E-01 | -1.76 | 6.29E-02 | 1.17 | 5.85E-01 | 2.07 | 2.06E-02 |
|  | *DUSP5* | 1.29 | 2.18E-02 | 1.79 | 2.24E-05 | 1.38 | 5.47E-03 | -1.43 | 2.71E-03 | 1.22 | 7.24E-02 | 1.74 | 4.09E-05 |
|  | *EEF1A1* | -1.65 | 2.84E-04 | -1.67 | 2.28E-04 | -1.01 | 9.21E-01 | 1.06 | 6.05E-01 | 1.06 | 6.36E-01 | -1.01 | 9.65E-01 |
|  | *EIF3CL* | 1.73 | 1.01E-03 | 2.29 | 1.32E-05 | 1.32 | 6.03E-02 | 1.29 | 8.50E-02 | 1.15 | 3.26E-01 | -1.12 | 4.27E-01 |
|  | *EP400* | -1.43 | 2.64E-04 | -1.66 | 4.77E-06 | -1.16 | 7.30E-02 | 1.04 | 6.60E-01 | 1.18 | 4.66E-02 | 1.14 | 1.08E-01 |
|  | *HNRNPH2* | 1.89 | 2.08E-03 | 2.08 | 6.44E-04 | 1.10 | 6.06E-01 | 1.15 | 4.28E-01 | -1.40 | 7.30E-02 | -1.62 | 1.42E-02 |
|  | *IARS* | 2.48 | 3.32E-05 | 2.05 | 4.15E-04 | -1.21 | 2.60E-01 | 1.35 | 8.73E-02 | -1.04 | 8.02E-01 | -1.41 | 5.39E-02 |
|  | *LMTK3* | -1.54 | 6.06E-03 | -1.25 | 1.23E-01 | 1.23 | 1.54E-01 | 1.23 | 1.47E-01 | 1.51 | 7.83E-03 | 1.23 | 1.58E-01 |
|  | *LOC647030* | 2.42 | 2.17E-05 | 2.36 | 3.01E-05 | -1.02 | 8.78E-01 | 1.60 | 7.32E-03 | 1.22 | 2.26E-01 | -1.32 | 9.41E-02 |
|  | *MARS* | 2.04 | 1.00E-09 | 1.82 | 1.51E-08 | -1.12 | 8.36E-02 | 1.10 | 1.50E-01 | 1.62 | 3.64E-07 | 1.47 | 6.42E-06 |
|  | *MRPL20* | 1.76 | 3.79E-06 | 1.92 | 5.93E-07 | 1.09 | 3.44E-01 | 1.18 | 7.44E-02 | -1.28 | 1.08E-02 | -1.51 | 1.65E-04 |
|  | *MRPL21* | 1.71 | 4.65E-04 | 1.92 | 6.18E-05 | 1.12 | 3.68E-01 | 1.31 | 4.61E-02 | -1.05 | 6.83E-01 | -1.38 | 1.98E-02 |
|  | *MRPL33* | 1.85 | 7.62E-05 | 2.15 | 5.46E-06 | 1.16 | 2.21E-01 | 1.04 | 7.47E-01 | -1.13 | 3.19E-01 | -1.18 | 1.93E-01 |
|  | *MRPL35* | 1.62 | 3.01E-05 | 1.71 | 8.82E-06 | 1.05 | 5.60E-01 | 1.16 | 1.15E-01 | -1.07 | 4.65E-01 | -1.23 | 2.73E-02 |
|  | *MRPS18C* | 1.71 | 7.26E-04 | 1.97 | 7.34E-05 | 1.15 | 3.11E-01 | 1.14 | 3.37E-01 | -1.20 | 1.84E-01 | -1.37 | 2.94E-02 |
|  | *PELO* | 2.05 | 3.71E-06 | 2.10 | 2.34E-06 | 1.03 | 8.16E-01 | 1.31 | 2.34E-02 | 1.05 | 6.63E-01 | -1.25 | 5.70E-02 |
|  | *PHF15* | -1.81 | 3.29E-04 | -1.83 | 2.70E-04 | -1.01 | 9.30E-01 | -1.79 | 4.04E-04 | -1.64 | 1.75E-03 | 1.09 | 5.19E-01 |
|  | *PTPRB* | -1.34 | 1.78E-03 | -1.66 | 5.41E-06 | -1.24 | 1.46E-02 | -1.44 | 2.53E-04 | -1.95 | 1.24E-07 | -1.36 | 1.18E-03 |
|  | *PTPRR* | 4.00 | 2.86E-07 | 2.70 | 2.26E-05 | -1.48 | 3.72E-02 | 1.46 | 4.33E-02 | 1.27 | 1.89E-01 | -1.15 | 4.29E-01 |
|  | *RIOK3* | 2.09 | 1.93E-04 | 2.15 | 1.32E-04 | 1.03 | 8.64E-01 | 1.50 | 1.99E-02 | 1.21 | 2.46E-01 | -1.24 | 1.91E-01 |
|  | *RPL10A* | 1.83 | 1.23E-03 | 2.13 | 1.40E-04 | 1.17 | 3.39E-01 | 1.49 | 2.05E-02 | 1.09 | 5.81E-01 | -1.37 | 6.33E-02 |
|  | *RPL14* | -1.55 | 4.30E-03 | -1.59 | 3.05E-03 | -1.02 | 8.78E-01 | 1.58 | 3.43E-03 | 1.87 | 2.09E-04 | 1.19 | 2.24E-01 |
|  | *RPL17* | 2.03 | 6.88E-04 | 2.35 | 1.10E-04 | 1.16 | 4.16E-01 | 2.17 | 2.95E-04 | 1.35 | 1.01E-01 | -1.61 | 1.34E-02 |
|  | *RPL21* | 1.69 | 3.58E-04 | 2.11 | 7.03E-06 | 1.25 | 8.11E-02 | 1.47 | 5.09E-03 | 1.13 | 3.37E-01 | -1.30 | 4.09E-02 |
|  | *RPL29* | 1.56 | 2.38E-04 | 1.73 | 2.25E-05 | 1.11 | 2.87E-01 | 1.49 | 6.96E-04 | -1.21 | 6.12E-02 | -1.80 | 9.54E-06 |
|  | *RPL37* | -1.21 | 1.50E-02 | -1.12 | 1.25E-01 | 1.08 | 2.93E-01 | 1.51 | 1.74E-05 | 1.54 | 8.85E-06 | 1.02 | 7.46E-01 |
|  | *RPL7L1* | -1.91 | 2.42E-03 | -2.06 | 9.82E-04 | -1.08 | 6.90E-01 | -1.47 | 5.21E-02 | 1.18 | 3.82E-01 | 1.73 | 8.09E-03 |
|  | *RPL9* | 1.32 | 2.63E-02 | 1.55 | 1.25E-03 | 1.18 | 1.78E-01 | 2.44 | 3.94E-07 | 1.24 | 7.54E-02 | -1.96 | 1.54E-05 |
|  | *RPLP0* | 2.29 | 5.93E-05 | 2.55 | 1.43E-05 | 1.11 | 5.08E-01 | 1.91 | 7.35E-04 | 1.34 | 7.96E-02 | -1.42 | 4.09E-02 |
|  | *RPS15A* | 1.36 | 1.87E-02 | 1.65 | 5.68E-04 | 1.21 | 1.29E-01 | 1.87 | 5.29E-05 | 1.33 | 2.73E-02 | -1.41 | 1.04E-02 |
|  | *RPS21* | 1.77 | 9.37E-05 | 1.98 | 1.27E-05 | 1.11 | 3.56E-01 | 1.19 | 1.55E-01 | -1.33 | 2.28E-02 | -1.58 | 8.87E-04 |
|  | *RPS26* | 2.05 | 1.59E-04 | 1.86 | 6.56E-04 | -1.10 | 5.29E-01 | 1.22 | 2.02E-01 | -1.08 | 6.24E-01 | -1.32 | 8.48E-02 |
|  | *RPS26P11* | 1.63 | 4.24E-06 | 1.55 | 1.79E-05 | -1.05 | 4.87E-01 | 1.01 | 9.15E-01 | -1.30 | 2.96E-03 | -1.31 | 2.33E-03 |
|  | *RPS7* | 1.62 | 4.71E-04 | 1.80 | 6.35E-05 | 1.11 | 3.72E-01 | 2.07 | 4.86E-06 | 1.26 | 5.94E-02 | -1.65 | 3.43E-04 |
|  | *SARS* | 1.93 | 8.95E-07 | 1.93 | 8.46E-07 | 1.00 | 9.76E-01 | 1.28 | 1.35E-02 | -1.03 | 7.80E-01 | -1.31 | 7.30E-03 |
|  | *SNORA67* | 1.02 | 8.86E-01 | 1.13 | 2.62E-01 | 1.11 | 3.25E-01 | -1.15 | 1.87E-01 | 1.68 | 7.66E-05 | 1.94 | 4.44E-06 |
|  | *TAOK1* | -2.04 | 2.42E-03 | -2.32 | 5.75E-04 | -1.14 | 5.26E-01 | -1.44 | 8.54E-02 | 1.66 | 2.18E-02 | 2.40 | 4.02E-04 |
|  |  |  |  |  |  |  |  |  |  |  |  |  |  |
| **Response to stress** | *AHSA2* | -1.64 | 8.24E-03 | -1.49 | 2.73E-02 | 1.10 | 5.79E-01 | -1.27 | 1.72E-01 | 1.07 | 6.78E-01 | 1.36 | 8.15E-02 |
|  | *AKR1B1* | 1.30 | 2.48E-02 | 1.41 | 4.70E-03 | 1.09 | 4.49E-01 | -1.77 | 4.32E-05 | -1.63 | 2.28E-04 | 1.09 | 4.53E-01 |
|  | *ALDH2* | -3.10 | 1.08E-08 | -3.10 | 1.08E-08 | -1.00 | 9.98E-01 | -1.03 | 8.15E-01 | -1.88 | 2.98E-05 | -1.83 | 4.94E-05 |
|  | *ALKBH1* | 1.24 | 7.37E-02 | 1.53 | 1.40E-03 | 1.23 | 7.78E-02 | 1.05 | 6.50E-01 | -1.26 | 5.52E-02 | -1.33 | 2.18E-02 |
|  | *ASF1A* | 1.75 | 1.73E-05 | 1.62 | 1.05E-04 | -1.09 | 4.07E-01 | 1.16 | 1.52E-01 | -1.25 | 3.14E-02 | -1.45 | 1.23E-03 |
|  | *ATF4* | 2.65 | 7.14E-09 | 2.73 | 4.24E-09 | 1.03 | 7.33E-01 | 1.24 | 3.66E-02 | 1.61 | 9.49E-05 | 1.30 | 1.37E-02 |
|  | *ATRN* | -1.39 | 1.57E-02 | -1.54 | 2.64E-03 | -1.11 | 4.24E-01 | 1.04 | 7.42E-01 | -1.12 | 3.65E-01 | -1.17 | 2.22E-01 |
|  | *C1orf124* | 1.44 | 2.57E-02 | 1.46 | 2.19E-02 | 1.01 | 9.40E-01 | -1.18 | 2.95E-01 | -1.45 | 2.28E-02 | -1.24 | 1.75E-01 |
|  | *C7orf68* | -1.45 | 2.75E-03 | -1.91 | 9.54E-06 | -1.32 | 1.76E-02 | 1.63 | 2.16E-04 | 1.64 | 2.06E-04 | 1.00 | 9.84E-01 |
|  | *CA9* | -1.35 | 1.00E-03 | -1.40 | 3.44E-04 | -1.04 | 6.35E-01 | 2.70 | 1.19E-10 | 2.23 | 3.79E-09 | -1.21 | 2.00E-02 |
|  | *CABC1* | -1.58 | 2.26E-05 | -1.67 | 5.28E-06 | -1.06 | 4.86E-01 | 1.11 | 2.02E-01 | 1.03 | 6.89E-01 | -1.08 | 3.72E-01 |
|  | *CHD1L* | -1.44 | 4.23E-03 | -1.40 | 7.29E-03 | 1.03 | 8.07E-01 | 1.24 | 7.49E-02 | 1.26 | 5.06E-02 | 1.02 | 8.41E-01 |
|  | *CUL4B* | 1.81 | 9.67E-04 | 2.51 | 8.41E-06 | 1.39 | 4.06E-02 | 1.20 | 2.38E-01 | 1.28 | 1.17E-01 | 1.07 | 6.74E-01 |
|  | *CYBA* | -2.03 | 6.66E-07 | -2.02 | 7.22E-07 | 1.00 | 9.66E-01 | -1.04 | 7.21E-01 | -1.07 | 5.14E-01 | -1.03 | 7.65E-01 |
|  | *DDB1* | -1.33 | 5.00E-02 | -1.33 | 4.86E-02 | -1.00 | 9.88E-01 | -1.30 | 6.61E-02 | 1.27 | 9.22E-02 | 1.65 | 1.52E-03 |
|  | *DNAJC4* | -1.96 | 2.58E-05 | -1.82 | 1.01E-04 | 1.08 | 5.31E-01 | 1.25 | 7.85E-02 | 1.26 | 7.14E-02 | 1.01 | 9.60E-01 |
|  | *DYSF* | -1.54 | 1.90E-02 | -1.99 | 6.43E-04 | -1.29 | 1.40E-01 | -2.36 | 6.58E-05 | -1.84 | 1.78E-03 | 1.28 | 1.52E-01 |
|  | *EFEMP2* | -2.28 | 1.70E-05 | -1.92 | 2.26E-04 | 1.19 | 2.42E-01 | 1.42 | 2.35E-02 | 1.49 | 1.17E-02 | 1.05 | 7.43E-01 |
|  | *ERO1L* | -2.14 | 1.65E-06 | -2.74 | 2.82E-08 | -1.28 | 3.34E-02 | 1.32 | 1.97E-02 | 1.53 | 1.00E-03 | 1.16 | 1.90E-01 |
|  | *ERRFI1* | -1.20 | 3.10E-02 | 1.06 | 4.57E-01 | 1.28 | 6.18E-03 | 1.49 | 7.19E-05 | 1.82 | 4.75E-07 | 1.22 | 2.16E-02 |
|  | *FAM129A* | 2.81 | 3.72E-05 | 2.39 | 2.35E-04 | -1.18 | 4.06E-01 | 1.22 | 3.17E-01 | 1.48 | 5.54E-02 | 1.21 | 3.22E-01 |
|  | *GTF2H1* | 1.40 | 3.10E-02 | 1.52 | 1.00E-02 | 1.08 | 5.98E-01 | 1.07 | 6.57E-01 | -1.22 | 1.78E-01 | -1.31 | 8.04E-02 |
|  | *HIGD1A* | 2.24 | 6.17E-05 | 2.32 | 3.70E-05 | 1.04 | 8.13E-01 | 1.21 | 2.35E-01 | -1.07 | 6.65E-01 | -1.30 | 1.12E-01 |
|  | *HIST1H2BK* | 1.90 | 2.63E-04 | 2.46 | 5.82E-06 | 1.29 | 8.83E-02 | 1.45 | 1.71E-02 | -1.02 | 8.79E-01 | -1.48 | 1.23E-02 |
|  | *HLA-B* | -1.43 | 1.65E-02 | -1.47 | 9.82E-03 | -1.03 | 8.10E-01 | -1.24 | 1.29E-01 | -1.05 | 7.25E-01 | 1.18 | 2.33E-01 |
|  | *HSPA2* | -1.76 | 5.16E-04 | -1.85 | 2.19E-04 | -1.05 | 7.03E-01 | -1.42 | 1.82E-02 | -1.82 | 2.96E-04 | -1.28 | 7.76E-02 |
|  | *HSPA4* | 1.51 | 5.82E-03 | 1.49 | 7.12E-03 | -1.01 | 9.28E-01 | 1.04 | 7.79E-01 | -1.26 | 9.29E-02 | -1.31 | 5.42E-02 |
|  | *HSPA8* | 1.93 | 2.16E-06 | 2.26 | 1.10E-07 | 1.17 | 1.21E-01 | 1.08 | 4.50E-01 | -1.11 | 3.08E-01 | -1.19 | 8.54E-02 |
|  | *HYOU1* | -1.65 | 2.45E-04 | -2.07 | 3.50E-06 | -1.25 | 5.79E-02 | -1.12 | 3.27E-01 | -1.03 | 7.67E-01 | 1.08 | 4.89E-01 |
|  | *IDH1* | 2.15 | 2.32E-06 | 2.14 | 2.53E-06 | -1.01 | 9.64E-01 | 1.31 | 2.71E-02 | -1.09 | 4.35E-01 | -1.44 | 4.91E-03 |
|  | *IRF7* | -2.71 | 2.20E-05 | -1.94 | 1.38E-03 | 1.40 | 7.35E-02 | -1.27 | 1.88E-01 | -1.02 | 8.95E-01 | 1.24 | 2.32E-01 |
|  | *KIAA1715* | -1.66 | 3.05E-04 | -1.82 | 5.48E-05 | -1.09 | 4.40E-01 | 1.06 | 6.24E-01 | 1.48 | 2.80E-03 | 1.40 | 8.38E-03 |
|  | *KYNU* | 1.53 | 3.91E-02 | 1.93 | 2.90E-03 | 1.26 | 2.39E-01 | 1.07 | 7.25E-01 | -1.04 | 8.57E-01 | -1.11 | 5.96E-01 |
|  | *MRPS11* | 1.38 | 2.58E-02 | 1.44 | 1.29E-02 | 1.04 | 7.46E-01 | -1.02 | 8.95E-01 | -1.30 | 6.19E-02 | -1.28 | 7.98E-02 |
|  | *MRPS35* | 2.01 | 9.99E-05 | 2.39 | 7.48E-06 | 1.19 | 2.33E-01 | 1.31 | 6.88E-02 | -1.11 | 4.85E-01 | -1.45 | 1.63E-02 |
|  | *NFE2L2* | 2.13 | 2.70E-04 | 2.33 | 8.31E-05 | 1.09 | 5.96E-01 | 1.13 | 4.62E-01 | -1.11 | 5.29E-01 | -1.26 | 1.81E-01 |
|  | *NFKBIZ* | -1.38 | 1.07E-01 | 1.08 | 6.80E-01 | 1.50 | 4.86E-02 | -1.71 | 1.15E-02 | 2.05 | 1.44E-03 | 3.51 | 3.58E-06 |
|  | *NOX4* | -1.80 | 1.86E-04 | -1.24 | 1.03E-01 | 1.45 | 8.36E-03 | -1.26 | 8.71E-02 | 1.02 | 8.54E-01 | 1.29 | 6.13E-02 |
|  | *NUDT1* | -1.92 | 1.22E-03 | -2.63 | 2.10E-05 | -1.37 | 7.82E-02 | -1.17 | 3.58E-01 | 1.23 | 2.44E-01 | 1.44 | 4.57E-02 |
|  | *OS9* | -1.38 | 2.09E-02 | -1.57 | 2.49E-03 | -1.13 | 3.40E-01 | -1.00 | 9.72E-01 | -1.29 | 6.60E-02 | -1.28 | 7.05E-02 |
|  | *PDIA5* | -1.63 | 1.73E-05 | -1.65 | 1.29E-05 | -1.01 | 8.89E-01 | -1.07 | 4.30E-01 | -1.34 | 2.77E-03 | -1.25 | 1.60E-02 |
|  | *PLOD1* | -2.66 | 8.16E-10 | -2.81 | 3.39E-10 | -1.06 | 5.22E-01 | -1.01 | 8.67E-01 | -1.15 | 1.16E-01 | -1.13 | 1.55E-01 |
|  | *PLOD2* | -1.58 | 6.65E-07 | -2.04 | 7.79E-10 | -1.30 | 5.05E-04 | 1.18 | 1.56E-02 | 1.10 | 1.37E-01 | -1.07 | 2.79E-01 |
|  | *PLSCR4* | -2.07 | 1.32E-03 | -2.00 | 1.88E-03 | 1.03 | 8.75E-01 | 1.09 | 6.65E-01 | -1.72 | 1.08E-02 | -1.87 | 4.12E-03 |
|  | *PMS2L5* | 1.42 | 2.55E-03 | 1.89 | 6.22E-06 | 1.32 | 1.20E-02 | 1.06 | 5.50E-01 | -1.29 | 2.22E-02 | -1.37 | 6.03E-03 |
|  | *PTX3* | 1.98 | 4.77E-04 | 1.42 | 4.33E-02 | -1.40 | 5.21E-02 | 1.54 | 1.49E-02 | 1.13 | 4.58E-01 | -1.36 | 6.90E-02 |
|  | *RAD51C* | 1.83 | 7.60E-05 | 1.80 | 9.71E-05 | -1.01 | 9.11E-01 | 1.21 | 1.27E-01 | -1.18 | 1.78E-01 | -1.43 | 7.64E-03 |
|  | *RBM3* | 2.22 | 1.04E-03 | 3.02 | 3.84E-05 | 1.36 | 1.49E-01 | 1.05 | 8.22E-01 | -1.46 | 8.29E-02 | -1.52 | 5.37E-02 |
|  | *SHFM1* | 1.81 | 2.77E-05 | 2.04 | 2.93E-06 | 1.13 | 2.83E-01 | 1.22 | 7.63E-02 | 1.09 | 4.16E-01 | -1.12 | 3.08E-01 |
|  | *SLC2A1* | -3.04 | 1.78E-06 | -4.59 | 1.99E-08 | -1.51 | 1.96E-02 | 1.43 | 3.91E-02 | -1.05 | 7.87E-01 | -1.49 | 2.23E-02 |
|  | *TFPI* | -1.40 | 8.51E-03 | -1.07 | 5.81E-01 | 1.32 | 2.80E-02 | 1.47 | 3.66E-03 | 1.25 | 6.57E-02 | -1.17 | 1.85E-01 |
|  | *TFRC* | 2.36 | 3.92E-05 | 2.16 | 1.25E-04 | -1.09 | 5.97E-01 | -1.06 | 7.21E-01 | -1.83 | 1.29E-03 | -1.73 | 2.88E-03 |
|  | *THBD* | 1.33 | 2.07E-02 | 1.41 | 6.68E-03 | 1.06 | 6.04E-01 | -1.07 | 5.31E-01 | 2.03 | 6.04E-06 | 2.18 | 1.72E-06 |
|  | *TNIP1* | -1.65 | 6.20E-04 | -1.63 | 8.18E-04 | 1.02 | 9.02E-01 | -1.06 | 6.23E-01 | 1.26 | 7.10E-02 | 1.34 | 2.64E-02 |
|  | *TXNRD2* | -1.33 | 3.11E-02 | -2.51 | 4.89E-07 | -1.89 | 5.11E-05 | 1.03 | 7.82E-01 | -1.37 | 1.69E-02 | -1.42 | 9.30E-03 |
|  | *WDR33* | -1.54 | 2.50E-03 | -1.40 | 1.42E-02 | 1.10 | 4.37E-01 | -1.22 | 1.21E-01 | 1.06 | 6.24E-01 | 1.30 | 4.74E-02 |
|  |  |  |  |  |  |  |  |  |  |  |  |  |  |
| **Transport** | *ABCA3* | -1.75 | 7.20E-06 | -2.05 | 2.28E-07 | -1.18 | 8.54E-02 | -1.18 | 7.42E-02 | -1.22 | 4.21E-02 | -1.03 | 7.73E-01 |
|  | *ABCC3* | -1.76 | 5.31E-04 | -2.02 | 5.30E-05 | -1.15 | 3.06E-01 | -1.56 | 3.93E-03 | -1.90 | 1.40E-04 | -1.22 | 1.50E-01 |
|  | *ABCG2* | -1.08 | 5.73E-01 | -1.01 | 9.18E-01 | 1.06 | 6.44E-01 | -1.44 | 1.14E-02 | -2.20 | 9.61E-06 | -1.53 | 4.36E-03 |
|  | *AP1S1* | 1.91 | 1.28E-04 | 2.32 | 5.92E-06 | 1.22 | 1.60E-01 | 1.20 | 1.82E-01 | -1.28 | 8.23E-02 | -1.54 | 4.67E-03 |
|  | *AP3M1* | 1.79 | 5.87E-05 | 2.35 | 4.33E-07 | 1.32 | 2.37E-02 | 1.50 | 1.92E-03 | -1.01 | 9.43E-01 | -1.51 | 1.64E-03 |
|  | *ASGR1* | -2.77 | 3.40E-07 | -2.40 | 2.65E-06 | 1.15 | 2.88E-01 | -1.08 | 5.54E-01 | 1.16 | 2.67E-01 | 1.26 | 9.73E-02 |
|  | *ATG4A* | 1.13 | 3.66E-01 | 1.19 | 2.04E-01 | 1.05 | 7.01E-01 | -1.70 | 6.91E-04 | -1.23 | 1.21E-01 | 1.37 | 2.43E-02 |
|  | *ATG4B* | 1.95 | 1.85E-05 | 2.43 | 4.24E-07 | 1.25 | 6.89E-02 | 1.07 | 5.73E-01 | -1.13 | 3.21E-01 | -1.20 | 1.28E-01 |
|  | *ATP1B3* | 2.04 | 6.15E-03 | 2.63 | 5.36E-04 | 1.29 | 2.86E-01 | -1.06 | 8.03E-01 | -1.51 | 9.20E-02 | -1.42 | 1.44E-01 |
|  | *ATP5F1* | 2.76 | 2.72E-04 | 3.20 | 6.45E-05 | 1.16 | 5.18E-01 | 1.39 | 1.63E-01 | -1.24 | 3.56E-01 | -1.72 | 2.73E-02 |
|  | *ATP6V0D1* | 2.01 | 6.21E-05 | 2.47 | 2.76E-06 | 1.23 | 1.47E-01 | -1.17 | 2.71E-01 | -1.34 | 4.12E-02 | -1.15 | 3.02E-01 |
|  | *ATP6V0E1* | 1.70 | 6.40E-05 | 2.00 | 2.44E-06 | 1.18 | 1.28E-01 | 1.12 | 2.68E-01 | -1.13 | 2.50E-01 | -1.27 | 3.16E-02 |
|  | *CACNA1H* | -1.52 | 2.84E-04 | -1.40 | 2.10E-03 | 1.09 | 3.81E-01 | 1.33 | 6.47E-03 | 1.27 | 2.15E-02 | -1.05 | 5.82E-01 |
|  | *CACNG6* | -1.43 | 8.44E-04 | -1.39 | 1.59E-03 | 1.03 | 7.81E-01 | -1.15 | 1.37E-01 | 1.44 | 6.06E-04 | 1.66 | 2.09E-05 |
|  | *CATSPER1* | 1.16 | 1.89E-01 | 1.19 | 1.19E-01 | 1.03 | 7.90E-01 | 1.19 | 1.17E-01 | 1.87 | 1.31E-05 | 1.57 | 4.49E-04 |
|  | *CATSPER2* | -2.65 | 9.09E-03 | -2.38 | 1.79E-02 | 1.11 | 7.54E-01 | -1.52 | 2.25E-01 | 1.09 | 8.05E-01 | 1.65 | 1.49E-01 |
|  | *CHMP1B* | 1.78 | 4.05E-03 | 2.20 | 2.85E-04 | 1.23 | 2.48E-01 | 1.23 | 2.56E-01 | 1.14 | 4.73E-01 | -1.08 | 6.65E-01 |
|  | *CHMP2B* | 2.12 | 3.96E-04 | 2.12 | 3.97E-04 | -1.00 | 9.99E-01 | 1.10 | 5.89E-01 | 1.03 | 8.62E-01 | -1.07 | 7.13E-01 |
|  | *CKLF* | 1.48 | 7.50E-03 | 1.45 | 1.09E-02 | -1.02 | 8.64E-01 | 1.15 | 2.95E-01 | -1.31 | 5.49E-02 | -1.50 | 5.78E-03 |
|  | *CLTA* | 1.47 | 2.61E-05 | 1.88 | 3.61E-08 | 1.28 | 2.41E-03 | 1.17 | 3.30E-02 | -1.10 | 2.05E-01 | -1.28 | 1.94E-03 |
|  | *COG5* | 2.03 | 2.45E-04 | 2.17 | 9.78E-05 | 1.07 | 6.79E-01 | 1.25 | 1.71E-01 | -1.04 | 7.88E-01 | -1.30 | 1.07E-01 |
|  | *CRCP* | -2.87 | 3.78E-03 | -2.62 | 7.10E-03 | 1.10 | 7.78E-01 | -1.66 | 1.26E-01 | 1.01 | 9.75E-01 | 1.68 | 1.19E-01 |
|  | *CRYM* | 1.72 | 3.49E-05 | 2.01 | 1.47E-06 | 1.17 | 1.34E-01 | 1.19 | 9.62E-02 | 1.71 | 4.16E-05 | 1.43 | 1.96E-03 |
|  | *CYB5R4* | 2.50 | 4.67E-07 | 2.66 | 1.90E-07 | 1.06 | 6.22E-01 | 1.27 | 6.51E-02 | 1.03 | 7.89E-01 | -1.23 | 1.08E-01 |
|  | *DAB2* | 1.77 | 1.36E-03 | 2.01 | 2.06E-04 | 1.14 | 4.07E-01 | 1.32 | 7.74E-02 | 1.52 | 1.19E-02 | 1.15 | 3.68E-01 |
|  | *DBI* | 1.87 | 1.27E-04 | 1.67 | 8.91E-04 | -1.12 | 3.89E-01 | 1.21 | 1.48E-01 | -1.15 | 2.84E-01 | -1.40 | 1.75E-02 |
|  | *DENND1A* | 1.76 | 1.54E-05 | 2.02 | 8.92E-07 | 1.15 | 1.66E-01 | -1.11 | 2.93E-01 | -1.00 | 9.60E-01 | 1.10 | 3.15E-01 |
|  | *DIRC2* | 1.57 | 7.51E-05 | 1.78 | 3.74E-06 | 1.14 | 1.64E-01 | -1.04 | 6.65E-01 | -1.18 | 7.86E-02 | -1.13 | 1.72E-01 |
|  | *DPH3* | 2.11 | 3.28E-06 | 2.14 | 2.49E-06 | 1.02 | 8.89E-01 | 1.09 | 4.34E-01 | -1.07 | 5.30E-01 | -1.18 | 1.66E-01 |
|  | *DSCR3* | 2.83 | 9.71E-08 | 2.79 | 1.20E-07 | -1.01 | 9.05E-01 | 1.26 | 7.36E-02 | -1.05 | 6.70E-01 | -1.33 | 3.14E-02 |
|  | *ELFN2* | -1.52 | 9.01E-04 | -1.54 | 6.95E-04 | -1.01 | 9.08E-01 | 1.36 | 9.58E-03 | 1.22 | 7.48E-02 | -1.11 | 3.27E-01 |
|  | *ENAH* | -1.40 | 4.28E-02 | -1.52 | 1.49E-02 | -1.08 | 6.14E-01 | -1.48 | 2.10E-02 | -1.06 | 7.26E-01 | 1.40 | 4.34E-02 |
|  | *ENSA* | 1.99 | 3.05E-06 | 2.13 | 8.30E-07 | 1.07 | 5.06E-01 | -1.07 | 5.39E-01 | -1.42 | 3.41E-03 | -1.33 | 1.33E-02 |
|  | *ERP29* | 1.88 | 6.09E-05 | 2.34 | 1.50E-06 | 1.25 | 8.49E-02 | 1.05 | 6.79E-01 | -1.24 | 9.30E-02 | -1.31 | 4.16E-02 |
|  | *ETFA* | 1.56 | 7.62E-03 | 1.79 | 9.81E-04 | 1.15 | 3.67E-01 | 1.28 | 1.13E-01 | -1.23 | 1.78E-01 | -1.58 | 6.68E-03 |
|  | *GARS* | 2.11 | 2.19E-06 | 2.09 | 2.71E-06 | -1.01 | 9.14E-01 | 1.15 | 2.15E-01 | 1.25 | 5.39E-02 | 1.09 | 4.48E-01 |
|  | *HBG1* | -1.67 | 1.34E-05 | -1.58 | 5.59E-05 | 1.06 | 5.06E-01 | 1.57 | 6.61E-05 | 1.58 | 5.77E-05 | 1.01 | 9.50E-01 |
|  | *HBG2* | -1.60 | 2.77E-04 | -1.79 | 2.43E-05 | -1.12 | 2.74E-01 | 1.64 | 1.50E-04 | 1.69 | 8.58E-05 | 1.03 | 7.99E-01 |
|  | *HERC1* | -2.19 | 1.52E-06 | -2.05 | 4.78E-06 | 1.07 | 5.64E-01 | 1.14 | 2.69E-01 | -1.04 | 7.61E-01 | -1.18 | 1.64E-01 |
|  | *KIF20A* | -1.61 | 4.99E-04 | -2.41 | 3.50E-07 | -1.49 | 2.20E-03 | -1.05 | 6.79E-01 | 1.22 | 9.83E-02 | 1.28 | 4.41E-02 |
|  | *KPNA2* | 2.16 | 4.19E-03 | 1.80 | 2.26E-02 | -1.20 | 4.44E-01 | 1.34 | 2.25E-01 | -1.64 | 4.90E-02 | -2.20 | 3.42E-03 |
|  | *LAPTM4B* | 1.70 | 5.98E-05 | 1.93 | 4.11E-06 | 1.14 | 2.11E-01 | 1.39 | 4.67E-03 | -1.19 | 1.09E-01 | -1.65 | 1.12E-04 |
|  | *MFSD6* | 1.37 | 8.57E-04 | 1.65 | 5.09E-06 | 1.21 | 2.73E-02 | -1.45 | 1.85E-04 | 1.15 | 9.53E-02 | 1.66 | 4.60E-06 |
|  | *MKKS* | 1.54 | 1.24E-03 | 1.61 | 5.76E-04 | 1.04 | 7.34E-01 | 1.07 | 5.70E-01 | -1.16 | 2.11E-01 | -1.24 | 7.69E-02 |
|  | *MMGT1* | 2.08 | 4.21E-07 | 2.47 | 1.95E-08 | 1.19 | 8.86E-02 | 1.26 | 2.76E-02 | 1.08 | 4.35E-01 | -1.16 | 1.27E-01 |
|  | *MOBKL3* | 2.00 | 1.94E-04 | 2.15 | 6.96E-05 | 1.07 | 6.43E-01 | 1.50 | 1.36E-02 | 1.04 | 8.09E-01 | -1.45 | 2.28E-02 |
|  | *MRPL45* | 2.70 | 1.55E-05 | 3.41 | 1.02E-06 | 1.26 | 1.85E-01 | 1.57 | 1.60E-02 | -1.04 | 8.21E-01 | -1.63 | 9.79E-03 |
|  | *NDUFB6* | 1.68 | 4.15E-04 | 1.86 | 6.92E-05 | 1.10 | 4.23E-01 | 1.17 | 2.14E-01 | -1.06 | 6.39E-01 | -1.24 | 9.45E-02 |
|  | *NPTX1* | -2.22 | 4.44E-07 | -2.26 | 3.31E-07 | -1.02 | 8.74E-01 | 1.12 | 2.73E-01 | 1.63 | 1.89E-04 | 1.45 | 2.33E-03 |
|  | *PCYOX1* | -2.78 | 4.56E-04 | -3.01 | 2.18E-04 | -1.08 | 7.42E-01 | -1.16 | 5.50E-01 | 1.02 | 9.23E-01 | 1.18 | 4.88E-01 |
|  | *PDIA4* | -1.89 | 1.71E-04 | -2.07 | 4.01E-05 | -1.10 | 5.10E-01 | -1.05 | 7.09E-01 | -1.17 | 2.69E-01 | -1.11 | 4.57E-01 |
|  | *PFKL* | -2.05 | 9.85E-07 | -1.99 | 1.73E-06 | 1.03 | 7.71E-01 | 1.06 | 5.46E-01 | 1.23 | 5.08E-02 | 1.16 | 1.57E-01 |
|  | *PKIA* | 1.70 | 1.79E-04 | 1.86 | 3.45E-05 | 1.09 | 4.55E-01 | 1.12 | 3.33E-01 | 1.33 | 2.18E-02 | 1.19 | 1.47E-01 |
|  | *PLIN2* | 1.56 | 1.30E-03 | 2.18 | 3.30E-06 | 1.39 | 1.16E-02 | 1.20 | 1.44E-01 | 1.03 | 7.92E-01 | -1.16 | 2.24E-01 |
|  | *PRICKLE1* | -1.57 | 9.36E-03 | -1.62 | 5.93E-03 | -1.03 | 8.36E-01 | -2.06 | 1.92E-04 | -1.44 | 3.11E-02 | 1.43 | 3.18E-02 |
|  | *RAE1* | 1.60 | 2.25E-03 | 1.77 | 4.17E-04 | 1.11 | 4.59E-01 | 1.14 | 3.47E-01 | -1.10 | 5.01E-01 | -1.24 | 1.16E-01 |
|  | *SAR1B* | 1.90 | 1.13E-03 | 1.83 | 1.85E-03 | -1.04 | 8.26E-01 | 1.08 | 6.56E-01 | -1.37 | 7.73E-02 | -1.47 | 3.18E-02 |
|  | *SEC62* | -1.69 | 7.88E-05 | -2.02 | 2.22E-06 | -1.20 | 9.91E-02 | -1.00 | 9.91E-01 | -1.02 | 8.42E-01 | -1.02 | 8.50E-01 |
|  | *SLC16A12* | -2.00 | 3.87E-03 | -1.85 | 8.64E-03 | 1.08 | 7.18E-01 | -1.52 | 5.93E-02 | -1.02 | 9.18E-01 | 1.49 | 7.23E-02 |
|  | *SLC16A14* | 1.88 | 1.86E-06 | 2.12 | 1.82E-07 | 1.12 | 2.23E-01 | -1.04 | 6.56E-01 | -1.16 | 1.19E-01 | -1.11 | 2.53E-01 |
|  | *SLC16A3* | -2.57 | 3.80E-12 | -2.85 | 6.26E-13 | -1.11 | 8.78E-02 | -1.02 | 7.32E-01 | -1.04 | 5.42E-01 | -1.02 | 7.87E-01 |
|  | *SLC23A3* | -1.87 | 1.27E-03 | -1.49 | 2.56E-02 | 1.25 | 1.84E-01 | -1.28 | 1.50E-01 | 1.04 | 8.11E-01 | 1.33 | 9.78E-02 |
|  | *SLC25A24* | 2.04 | 1.83E-03 | 2.28 | 5.37E-04 | 1.11 | 5.88E-01 | 1.24 | 2.79E-01 | -1.15 | 4.79E-01 | -1.43 | 8.25E-02 |
|  | *SLC25A26* | 1.91 | 1.71E-05 | 2.32 | 5.50E-07 | 1.22 | 9.62E-02 | 1.18 | 1.60E-01 | -1.14 | 2.47E-01 | -1.34 | 1.59E-02 |
|  | *SLC25A37* | -2.59 | 9.92E-04 | -2.22 | 4.11E-03 | 1.17 | 5.30E-01 | -1.17 | 5.15E-01 | 1.52 | 1.01E-01 | 1.79 | 2.77E-02 |
|  | *SLC2A12* | -1.62 | 4.14E-04 | -2.09 | 3.33E-06 | -1.29 | 3.44E-02 | 1.39 | 8.35E-03 | -1.47 | 2.78E-03 | -2.05 | 4.79E-06 |
|  | *SLC2A3* | -1.89 | 7.07E-08 | -1.59 | 5.60E-06 | 1.19 | 2.91E-02 | -1.06 | 4.60E-01 | 1.09 | 2.46E-01 | 1.15 | 6.65E-02 |
|  | *SLC30A1* | 2.09 | 5.30E-08 | 1.90 | 3.79E-07 | -1.10 | 2.73E-01 | 1.12 | 1.86E-01 | -1.05 | 5.46E-01 | -1.18 | 6.18E-02 |
|  | *SLC35E1* | -2.67 | 3.07E-03 | -2.83 | 1.98E-03 | -1.06 | 8.45E-01 | -1.64 | 1.03E-01 | -1.05 | 8.69E-01 | 1.56 | 1.39E-01 |
|  | *SLC35F5* | 1.98 | 6.78E-04 | 1.64 | 8.63E-03 | -1.21 | 2.65E-01 | 1.30 | 1.38E-01 | -1.13 | 4.83E-01 | -1.46 | 3.58E-02 |
|  | *SLC38A6* | 1.79 | 1.62E-05 | 1.97 | 2.30E-06 | 1.10 | 3.44E-01 | 1.09 | 3.83E-01 | 1.10 | 3.52E-01 | 1.01 | 9.52E-01 |
|  | *SLC3A2* | 2.90 | 3.28E-05 | 4.19 | 7.41E-07 | 1.45 | 7.28E-02 | 1.10 | 6.23E-01 | 1.03 | 8.82E-01 | -1.07 | 7.30E-01 |
|  | *SLC45A3* | -1.58 | 1.41E-04 | -1.68 | 3.24E-05 | -1.07 | 5.02E-01 | -1.50 | 4.55E-04 | -1.38 | 3.22E-03 | 1.09 | 3.90E-01 |
|  | *SLC4A5* | -2.56 | 2.60E-03 | -2.47 | 3.50E-03 | 1.04 | 8.95E-01 | -1.54 | 1.26E-01 | 1.42 | 2.13E-01 | 2.18 | 9.65E-03 |
|  | *SLC4A7* | -1.36 | 1.34E-02 | -1.12 | 3.19E-01 | 1.21 | 1.03E-01 | -1.06 | 5.96E-01 | 1.35 | 1.48E-02 | 1.44 | 4.60E-03 |
|  | *SLC5A3* | -2.49 | 3.17E-08 | -2.83 | 4.37E-09 | -1.13 | 2.20E-01 | -1.19 | 9.88E-02 | -1.11 | 2.87E-01 | 1.07 | 5.28E-01 |
|  | *SLC7A11* | 2.01 | 2.89E-07 | 2.33 | 1.59E-08 | 1.16 | 1.02E-01 | 1.10 | 2.99E-01 | 1.06 | 5.20E-01 | -1.04 | 6.84E-01 |
|  | *SLC7A5* | 2.35 | 5.99E-05 | 2.47 | 3.08E-05 | 1.05 | 7.58E-01 | 1.28 | 1.52E-01 | 1.99 | 5.47E-04 | 1.56 | 1.48E-02 |
|  | *SLCO2A1* | -1.05 | 7.42E-01 | -1.36 | 4.45E-02 | -1.30 | 8.44E-02 | -2.10 | 5.94E-05 | -2.66 | 1.99E-06 | -1.27 | 1.13E-01 |
|  | *SLCO4A1* | -5.46 | 8.38E-12 | -5.94 | 3.71E-12 | -1.09 | 4.56E-01 | -2.59 | 8.10E-08 | -1.97 | 8.21E-06 | 1.31 | 2.32E-02 |
|  | *SMG5* | 1.57 | 1.87E-04 | 1.45 | 1.26E-03 | -1.09 | 3.99E-01 | -1.07 | 5.15E-01 | -1.31 | 1.19E-02 | -1.23 | 4.69E-02 |
|  | *SNAP23* | 1.75 | 6.60E-03 | 2.39 | 1.47E-04 | 1.37 | 1.03E-01 | 1.10 | 5.97E-01 | -1.25 | 2.29E-01 | -1.38 | 9.13E-02 |
|  | *SNCG* | -1.80 | 2.86E-04 | -1.69 | 8.53E-04 | 1.07 | 6.29E-01 | -1.08 | 5.87E-01 | 1.16 | 2.75E-01 | 1.25 | 1.10E-01 |
|  | *SNX21* | -1.65 | 2.78E-04 | -1.54 | 1.05E-03 | 1.07 | 5.57E-01 | 1.34 | 1.67E-02 | 1.67 | 2.09E-04 | 1.25 | 6.21E-02 |
|  | *SNX5* | 3.30 | 7.36E-05 | 3.98 | 1.37E-05 | 1.20 | 4.36E-01 | 1.71 | 3.46E-02 | 1.02 | 9.37E-01 | -1.67 | 4.07E-02 |
|  | *SNX7* | 2.16 | 5.71E-05 | 2.28 | 2.58E-05 | 1.06 | 7.12E-01 | 1.15 | 3.45E-01 | -1.14 | 3.72E-01 | -1.32 | 7.54E-02 |
|  | *SRP54* | 1.49 | 1.64E-03 | 2.00 | 5.04E-06 | 1.34 | 1.47E-02 | 1.01 | 9.19E-01 | -1.17 | 1.63E-01 | -1.18 | 1.36E-01 |
|  | *SRP9* | 2.07 | 2.69E-03 | 2.51 | 3.62E-04 | 1.21 | 3.79E-01 | 1.21 | 3.85E-01 | -1.24 | 3.15E-01 | -1.50 | 7.05E-02 |
|  | *STEAP1* | 2.25 | 1.12E-05 | 2.42 | 3.79E-06 | 1.08 | 5.95E-01 | 1.05 | 7.11E-01 | -1.28 | 8.61E-02 | -1.34 | 4.17E-02 |
|  | *STX1A* | -1.19 | 1.58E-01 | -1.20 | 1.49E-01 | -1.00 | 9.73E-01 | -1.20 | 1.51E-01 | 1.63 | 7.15E-04 | 1.96 | 2.73E-05 |
|  | *STXBP5* | 1.83 | 4.11E-07 | 2.01 | 5.36E-08 | 1.10 | 2.58E-01 | 1.09 | 2.79E-01 | 1.09 | 2.84E-01 | -1.00 | 9.92E-01 |
|  | *SYPL1* | 2.33 | 6.41E-04 | 2.96 | 5.19E-05 | 1.27 | 2.66E-01 | 1.32 | 1.94E-01 | -1.39 | 1.30E-01 | -1.83 | 8.78E-03 |
|  | *TCN2* | -1.00 | 9.93E-01 | -1.07 | 6.40E-01 | -1.07 | 6.46E-01 | -2.29 | 1.25E-05 | -2.45 | 4.96E-06 | -1.07 | 6.53E-01 |
|  | *THOC3* | 2.04 | 2.07E-03 | 2.02 | 2.30E-03 | -1.01 | 9.64E-01 | 1.20 | 3.68E-01 | -1.15 | 4.90E-01 | -1.38 | 1.21E-01 |
|  | *THOC4* | 1.48 | 1.54E-02 | 1.19 | 2.56E-01 | -1.25 | 1.50E-01 | -1.03 | 8.24E-01 | -1.37 | 4.54E-02 | -1.32 | 7.02E-02 |
|  | *TIMM10* | 1.63 | 5.67E-05 | 1.35 | 4.51E-03 | -1.20 | 6.25E-02 | 1.05 | 6.22E-01 | -1.07 | 4.70E-01 | -1.12 | 2.31E-01 |
|  | *TINAGL1* | -1.78 | 9.91E-05 | -2.17 | 2.89E-06 | -1.22 | 1.05E-01 | 1.04 | 7.56E-01 | 1.04 | 7.46E-01 | 1.00 | 9.89E-01 |
|  | *TMEM38B* | 1.63 | 4.03E-04 | 1.78 | 6.86E-05 | 1.10 | 4.28E-01 | 1.34 | 1.73E-02 | -1.01 | 9.38E-01 | -1.36 | 1.46E-02 |
|  | *TOMM5* | 1.98 | 6.47E-05 | 1.84 | 2.05E-04 | -1.07 | 6.02E-01 | 1.24 | 1.17E-01 | -1.07 | 6.21E-01 | -1.33 | 4.55E-02 |
|  | *UQCRFS1* | 1.70 | 2.19E-05 | 1.56 | 1.63E-04 | -1.09 | 3.62E-01 | 1.06 | 5.27E-01 | -1.27 | 1.90E-02 | -1.35 | 4.73E-03 |
|  | *VPS13A* | -1.76 | 9.47E-05 | -1.92 | 1.88E-05 | -1.09 | 4.56E-01 | -1.47 | 3.33E-03 | -1.33 | 2.21E-02 | 1.10 | 3.93E-01 |
|  | *VPS24* | 1.99 | 2.09E-03 | 2.31 | 3.64E-04 | 1.16 | 4.42E-01 | 1.27 | 2.27E-01 | -1.06 | 7.61E-01 | -1.35 | 1.36E-01 |
|  | *XPOT* | 2.49 | 6.63E-05 | 2.75 | 2.07E-05 | 1.10 | 5.88E-01 | 1.26 | 2.11E-01 | 1.04 | 8.45E-01 | -1.21 | 2.86E-01 |
|  |  |  |  |  |  |  |  |  |  |  |  |  |  |
| **Transcription** | *AIRE* | -2.18 | 1.16E-03 | -2.02 | 2.66E-03 | 1.08 | 7.14E-01 | -1.35 | 1.56E-01 | 1.23 | 3.16E-01 | 1.66 | 2.17E-02 |
|  | *ATF3* | 2.02 | 2.08E-02 | 2.25 | 9.07E-03 | 1.11 | 7.01E-01 | 2.07 | 1.74E-02 | 2.99 | 9.32E-04 | 1.45 | 1.99E-01 |
|  | *BCKDHA* | -2.05 | 1.57E-06 | -1.96 | 3.58E-06 | 1.04 | 6.76E-01 | -1.21 | 7.79E-02 | -1.20 | 8.79E-02 | 1.01 | 9.49E-01 |
|  | *C14orf43* | -1.78 | 5.73E-06 | -1.76 | 7.74E-06 | 1.01 | 8.83E-01 | -1.16 | 1.29E-01 | -1.15 | 1.53E-01 | 1.01 | 9.24E-01 |
|  | *CCNC* | 2.08 | 1.32E-03 | 2.69 | 7.16E-05 | 1.29 | 2.02E-01 | 1.29 | 2.06E-01 | -1.15 | 4.81E-01 | -1.48 | 5.73E-02 |
|  | *ECD* | 1.54 | 4.46E-03 | 1.96 | 8.28E-05 | 1.27 | 8.80E-02 | 1.09 | 5.14E-01 | -1.16 | 2.79E-01 | -1.27 | 9.17E-02 |
|  | *EGR1* | -1.43 | 6.97E-02 | -1.23 | 2.74E-01 | 1.16 | 4.34E-01 | -1.75 | 7.52E-03 | 1.12 | 5.55E-01 | 1.96 | 2.00E-03 |
|  | *EIF1B* | 2.22 | 5.27E-06 | 2.60 | 4.64E-07 | 1.17 | 2.20E-01 | 1.40 | 1.43E-02 | 1.02 | 8.48E-01 | -1.37 | 2.16E-02 |
|  | *ETS2* | 1.65 | 6.02E-04 | 1.66 | 5.45E-04 | 1.01 | 9.65E-01 | -1.64 | 6.69E-04 | -1.92 | 4.05E-05 | -1.17 | 2.15E-01 |
|  | *FBXO7* | 1.60 | 7.57E-05 | 2.15 | 1.43E-07 | 1.34 | 4.82E-03 | 1.16 | 1.28E-01 | -1.00 | 9.92E-01 | -1.16 | 1.26E-01 |
|  | *FOXD1* | 1.86 | 9.80E-04 | 2.43 | 2.41E-05 | 1.31 | 1.06E-01 | 1.21 | 2.38E-01 | 1.27 | 1.51E-01 | 1.04 | 7.85E-01 |
|  | *FUBP3* | 2.15 | 7.18E-04 | 2.19 | 5.76E-04 | 1.02 | 9.22E-01 | 1.25 | 2.45E-01 | 1.04 | 8.42E-01 | -1.21 | 3.31E-01 |
|  | *GTF2A2* | 1.82 | 5.19E-05 | 1.78 | 8.06E-05 | -1.02 | 8.40E-01 | 1.02 | 8.84E-01 | -1.26 | 5.96E-02 | -1.28 | 4.46E-02 |
|  | *GTF2IRD2B* | 1.84 | 5.30E-04 | 2.26 | 2.27E-05 | 1.23 | 1.63E-01 | 1.29 | 9.59E-02 | -1.01 | 9.54E-01 | -1.30 | 8.61E-02 |
|  | *HES6* | 1.95 | 5.66E-07 | 1.88 | 1.20E-06 | -1.04 | 6.94E-01 | 1.09 | 3.50E-01 | 1.33 | 4.80E-03 | 1.22 | 3.69E-02 |
|  | *HMGA2* | 1.91 | 1.07E-06 | 2.08 | 2.06E-07 | 1.08 | 3.78E-01 | 1.22 | 3.88E-02 | 1.78 | 4.81E-06 | 1.46 | 5.48E-04 |
|  | *HNRNPU* | -1.63 | 7.28E-05 | -2.05 | 5.80E-07 | -1.26 | 2.68E-02 | -1.07 | 4.94E-01 | 1.18 | 9.34E-02 | 1.27 | 2.38E-02 |
|  | *INSM2* | 1.18 | 1.14E-01 | 1.54 | 4.59E-04 | 1.30 | 1.77E-02 | -1.92 | 4.00E-06 | -1.04 | 6.94E-01 | 1.85 | 8.93E-06 |
|  | *IRF2BP2* | -1.32 | 1.29E-01 | -1.28 | 1.71E-01 | 1.03 | 8.70E-01 | 1.42 | 6.05E-02 | -1.04 | 8.17E-01 | -1.48 | 3.81E-02 |
|  | *IRF6* | -2.06 | 5.89E-06 | -2.09 | 4.53E-06 | -1.02 | 8.97E-01 | -1.33 | 2.38E-02 | -1.19 | 1.47E-01 | 1.12 | 3.53E-01 |
|  | *KIAA0427* | -1.62 | 1.12E-03 | -1.80 | 1.77E-04 | -1.11 | 4.16E-01 | -1.24 | 9.80E-02 | 1.02 | 8.86E-01 | 1.27 | 7.49E-02 |
|  | *L3MBTL3* | 1.37 | 3.36E-03 | 1.70 | 2.16E-05 | 1.24 | 3.31E-02 | -1.33 | 7.41E-03 | -1.10 | 3.46E-01 | 1.21 | 5.53E-02 |
|  | *LMO4* | 2.32 | 2.65E-07 | 1.85 | 1.68E-05 | -1.26 | 4.52E-02 | 1.13 | 2.53E-01 | 1.04 | 7.12E-01 | -1.09 | 4.32E-01 |
|  | *LOC100134189* | 1.66 | 3.71E-06 | 1.83 | 3.69E-07 | 1.10 | 2.39E-01 | 1.21 | 2.39E-02 | -1.10 | 2.25E-01 | -1.34 | 1.56E-03 |
|  | *LOC286512* | -1.36 | 1.20E-01 | -1.35 | 1.22E-01 | 1.00 | 9.89E-01 | 1.20 | 3.40E-01 | 1.52 | 3.64E-02 | 1.27 | 2.16E-01 |
|  | *MCM8* | -2.36 | 7.03E-04 | -2.29 | 9.73E-04 | 1.03 | 8.86E-01 | -1.66 | 2.75E-02 | 1.05 | 8.20E-01 | 1.74 | 1.70E-02 |
|  | *MIF4GD* | 1.47 | 4.86E-05 | 1.65 | 1.87E-06 | 1.12 | 1.27E-01 | -1.09 | 2.32E-01 | -1.27 | 3.83E-03 | -1.16 | 5.20E-02 |
|  | *MKI67IP* | 1.59 | 2.69E-03 | 1.43 | 1.45E-02 | -1.11 | 4.50E-01 | 1.14 | 3.32E-01 | -1.30 | 6.74E-02 | -1.48 | 8.69E-03 |
|  | *MOV10* | -1.89 | 6.96E-04 | -1.87 | 8.13E-04 | 1.01 | 9.45E-01 | -1.29 | 1.18E-01 | 1.07 | 6.53E-01 | 1.39 | 5.00E-02 |
|  | *MXD4* | -2.22 | 4.63E-07 | -2.04 | 2.17E-06 | 1.09 | 4.23E-01 | -1.18 | 1.21E-01 | -1.30 | 2.01E-02 | -1.10 | 3.70E-01 |
|  | *NFE2L3* | -1.61 | 3.04E-03 | -2.59 | 2.09E-06 | -1.61 | 3.03E-03 | 1.34 | 4.82E-02 | 1.30 | 7.57E-02 | -1.03 | 8.17E-01 |
|  | *NGDN* | 1.79 | 1.14E-03 | 1.95 | 3.35E-04 | 1.09 | 5.87E-01 | 1.17 | 3.00E-01 | -1.27 | 1.31E-01 | -1.49 | 1.63E-02 |
|  | *PIR* | 1.47 | 5.51E-03 | 1.35 | 2.49E-02 | -1.09 | 4.89E-01 | 1.05 | 7.07E-01 | -1.42 | 9.92E-03 | -1.49 | 4.31E-03 |
|  | *POLR2B* | 1.94 | 9.30E-04 | 1.66 | 7.11E-03 | -1.17 | 3.71E-01 | 1.27 | 1.73E-01 | -1.18 | 3.32E-01 | -1.50 | 2.65E-02 |
|  | *POLR2D* | 1.71 | 1.14E-04 | 1.48 | 2.13E-03 | -1.16 | 2.02E-01 | 1.09 | 4.62E-01 | 1.05 | 6.68E-01 | -1.04 | 7.56E-01 |
|  | *POLR2L* | 2.01 | 1.60E-06 | 1.99 | 1.87E-06 | -1.01 | 9.35E-01 | -1.21 | 7.55E-02 | -1.51 | 6.25E-04 | -1.25 | 3.75E-02 |
|  | *RPL6* | 1.71 | 2.81E-03 | 1.82 | 1.12E-03 | 1.07 | 6.86E-01 | 1.33 | 8.32E-02 | -1.33 | 8.18E-02 | -1.77 | 1.72E-03 |
|  | *SCML1* | 2.51 | 1.16E-05 | 2.22 | 6.33E-05 | -1.13 | 4.30E-01 | 1.04 | 8.07E-01 | 1.19 | 2.75E-01 | 1.14 | 3.92E-01 |
|  | *SNAPC5* | 1.80 | 2.12E-06 | 1.69 | 8.53E-06 | -1.06 | 4.91E-01 | 1.01 | 9.14E-01 | -1.14 | 1.48E-01 | -1.15 | 1.22E-01 |
|  | *SOX7* | -2.21 | 3.99E-05 | -1.91 | 3.53E-04 | 1.16 | 3.29E-01 | -1.55 | 7.96E-03 | -1.60 | 4.96E-03 | -1.03 | 8.32E-01 |
|  | *SOX8* | -1.95 | 2.08E-05 | -1.85 | 5.20E-05 | 1.05 | 6.70E-01 | 1.14 | 2.64E-01 | 1.09 | 4.90E-01 | -1.05 | 6.60E-01 |
|  | *SPOCD1* | 1.36 | 4.25E-02 | 1.76 | 7.11E-04 | 1.30 | 7.48E-02 | 1.15 | 3.29E-01 | 2.01 | 8.99E-05 | 1.75 | 8.20E-04 |
|  | *SYNCRIP* | 1.68 | 2.05E-04 | 1.50 | 2.07E-03 | -1.12 | 3.11E-01 | -1.11 | 3.49E-01 | -1.40 | 7.95E-03 | -1.25 | 5.83E-02 |
|  | *TCEA1* | 2.32 | 2.72E-04 | 2.77 | 3.49E-05 | 1.19 | 3.55E-01 | 1.23 | 2.75E-01 | -1.17 | 4.03E-01 | -1.45 | 6.30E-02 |
|  | *TCEA2* | -1.83 | 1.18E-05 | -1.64 | 1.27E-04 | 1.12 | 2.77E-01 | 1.13 | 2.35E-01 | 1.02 | 8.12E-01 | -1.11 | 3.36E-01 |
|  | *TCEAL1* | 2.02 | 2.76E-04 | 2.77 | 3.96E-06 | 1.37 | 5.88E-02 | 1.08 | 6.12E-01 | -1.30 | 1.15E-01 | -1.40 | 4.33E-02 |
|  | *TCF3* | -1.61 | 2.77E-04 | -1.87 | 1.25E-05 | -1.16 | 1.65E-01 | -1.07 | 5.14E-01 | 1.27 | 3.45E-02 | 1.36 | 8.51E-03 |
|  | *TFB2M* | 2.01 | 8.33E-07 | 2.04 | 6.02E-07 | 1.02 | 8.63E-01 | 1.11 | 3.08E-01 | -1.26 | 2.77E-02 | -1.39 | 2.89E-03 |
|  | *TMPO* | 2.12 | 3.37E-03 | 1.99 | 6.54E-03 | -1.07 | 7.67E-01 | 1.11 | 6.58E-01 | -1.47 | 1.03E-01 | -1.62 | 4.37E-02 |
|  | *TSC22D1* | 3.59 | 7.04E-07 | 5.66 | 8.25E-09 | 1.57 | 1.69E-02 | 1.40 | 6.50E-02 | 1.38 | 7.93E-02 | -1.02 | 9.17E-01 |
|  | *UBA3* | 2.11 | 2.37E-03 | 2.41 | 5.84E-04 | 1.14 | 5.37E-01 | 1.23 | 3.44E-01 | -1.37 | 1.53E-01 | -1.68 | 2.41E-02 |
|  | *YAF2* | -1.96 | 8.48E-06 | -2.12 | 2.07E-06 | -1.08 | 4.85E-01 | -1.00 | 9.97E-01 | 1.21 | 9.50E-02 | 1.21 | 9.44E-02 |
|  | *ZNF323* | -1.43 | 6.74E-02 | -1.35 | 1.16E-01 | 1.06 | 7.72E-01 | -1.38 | 9.68E-02 | -2.26 | 2.97E-04 | -1.64 | 1.41E-02 |
|  | *ZNF395* | -2.03 | 1.41E-05 | -1.97 | 2.48E-05 | 1.03 | 7.88E-01 | 1.09 | 4.64E-01 | 1.03 | 7.86E-01 | -1.06 | 6.42E-01 |
|  | *ZNF430* | -1.83 | 8.52E-03 | -1.76 | 1.31E-02 | 1.04 | 8.44E-01 | -1.58 | 3.79E-02 | 1.02 | 9.14E-01 | 1.62 | 3.04E-02 |
|  | *ZNF467* | -4.36 | 5.43E-09 | -3.58 | 4.79E-08 | 1.22 | 1.85E-01 | 1.18 | 2.61E-01 | -1.05 | 7.38E-01 | -1.24 | 1.51E-01 |
|  | *ZNF486* | -2.24 | 4.93E-03 | -2.24 | 4.90E-03 | -1.00 | 9.98E-01 | -1.51 | 1.20E-01 | 1.19 | 5.05E-01 | 1.79 | 3.27E-02 |
|  | *ZNF581* | -1.88 | 6.67E-05 | -1.62 | 9.59E-04 | 1.16 | 2.41E-01 | 1.49 | 4.04E-03 | 1.18 | 1.90E-01 | -1.27 | 6.94E-02 |
|  | *ZNF652* | -2.52 | 5.59E-03 | -2.25 | 1.28E-02 | 1.12 | 7.08E-01 | -1.82 | 5.65E-02 | 1.13 | 6.90E-01 | 2.05 | 2.50E-02 |
|  | *ZNF669* | -1.84 | 1.22E-02 | -2.17 | 2.29E-03 | -1.18 | 4.56E-01 | -1.33 | 2.07E-01 | 1.15 | 5.36E-01 | 1.53 | 6.82E-02 |
|  | *ZNF83* | -1.13 | 3.00E-01 | -1.13 | 3.00E-01 | -1.00 | 9.99E-01 | -1.15 | 2.28E-01 | 1.21 | 1.10E-01 | 1.39 | 8.96E-03 |
|  |  |  |  |  |  |  |  |  |  |  |  |  |  |
| **Proteolysis** | *BTBD1* | 2.49 | 3.22E-04 | 2.73 | 1.22E-04 | 1.10 | 6.64E-01 | 1.18 | 4.32E-01 | -1.33 | 1.80E-01 | -1.57 | 4.13E-02 |
|  | *CAPN11* | -2.04 | 1.52E-05 | -2.25 | 2.99E-06 | -1.10 | 4.31E-01 | -1.23 | 1.00E-01 | 1.09 | 5.02E-01 | 1.34 | 2.63E-02 |
|  | *CPA4* | 1.82 | 1.88E-03 | -1.01 | 9.41E-01 | -1.85 | 1.60E-03 | 3.57 | 4.24E-07 | 3.20 | 1.45E-06 | -1.12 | 5.18E-01 |
|  | *CPD* | -2.02 | 7.23E-06 | -2.11 | 3.54E-06 | -1.04 | 7.25E-01 | -1.01 | 9.31E-01 | -1.25 | 6.44E-02 | -1.24 | 7.61E-02 |
|  | *CTSC* | 1.32 | 7.15E-02 | 1.34 | 6.24E-02 | 1.01 | 9.44E-01 | 1.39 | 3.75E-02 | -1.20 | 2.36E-01 | -1.66 | 2.73E-03 |
|  | *CTSL1* | 2.27 | 4.89E-04 | 3.25 | 9.18E-06 | 1.43 | 7.97E-02 | 1.23 | 2.99E-01 | -1.07 | 7.18E-01 | -1.32 | 1.68E-01 |
|  | *ECE2* | 1.45 | 9.51E-05 | 1.65 | 2.52E-06 | 1.14 | 9.53E-02 | -1.03 | 6.96E-01 | -1.22 | 1.58E-02 | -1.18 | 3.59E-02 |
|  | *ERMP1* | -1.79 | 6.42E-05 | -2.05 | 4.91E-06 | -1.15 | 2.31E-01 | -1.18 | 1.57E-01 | -1.81 | 4.83E-05 | -1.54 | 1.23E-03 |
|  | *FBXL18* | 1.15 | 1.36E-01 | -1.38 | 2.36E-03 | -1.60 | 7.52E-05 | -1.30 | 1.03E-02 | -1.97 | 7.83E-07 | -1.51 | 2.78E-04 |
|  | *FBXL20* | 1.04 | 7.13E-01 | 1.56 | 1.96E-04 | 1.51 | 4.46E-04 | -1.05 | 5.98E-01 | -1.04 | 6.86E-01 | 1.01 | 9.02E-01 |
|  | *FBXO22* | 1.88 | 2.87E-05 | 1.83 | 4.60E-05 | -1.03 | 8.26E-01 | 1.52 | 1.64E-03 | -1.02 | 8.74E-01 | -1.55 | 1.14E-03 |
|  | *HECW2* | 2.01 | 1.50E-09 | 1.63 | 3.24E-07 | -1.23 | 3.46E-03 | 1.06 | 3.79E-01 | 1.64 | 2.52E-07 | 1.55 | 1.33E-06 |
|  | *ISG15* | -3.05 | 7.49E-06 | -1.85 | 2.99E-03 | 1.65 | 1.26E-02 | -1.71 | 8.02E-03 | -1.43 | 6.27E-02 | 1.20 | 3.33E-01 |
|  | *KIAA0368* | -1.45 | 1.70E-05 | -1.64 | 4.27E-07 | -1.13 | 7.47E-02 | -1.30 | 6.79E-04 | -1.19 | 1.37E-02 | 1.09 | 1.89E-01 |
|  | *LAP3* | 1.46 | 2.38E-03 | 1.62 | 2.71E-04 | 1.11 | 3.42E-01 | 1.12 | 2.97E-01 | -1.31 | 1.99E-02 | -1.47 | 1.91E-03 |
|  | *LOC643668* | 2.48 | 3.95E-04 | 2.98 | 5.62E-05 | 1.21 | 3.83E-01 | 1.29 | 2.35E-01 | -1.17 | 4.73E-01 | -1.51 | 6.53E-02 |
|  | *LOC644877* | 1.76 | 1.32E-03 | 2.19 | 5.61E-05 | 1.24 | 1.68E-01 | 1.19 | 2.50E-01 | -1.25 | 1.58E-01 | -1.49 | 1.58E-02 |
|  | *LOC652826* | 1.96 | 4.27E-05 | 2.33 | 2.66E-06 | 1.19 | 1.90E-01 | 1.22 | 1.28E-01 | 1.01 | 9.33E-01 | -1.21 | 1.48E-01 |
|  | *MME* | 2.67 | 1.30E-07 | 2.81 | 5.97E-08 | 1.05 | 6.54E-01 | 1.73 | 1.91E-04 | 1.52 | 2.27E-03 | -1.14 | 2.80E-01 |
|  | *MMP10* | 3.18 | 1.27E-08 | 5.16 | 4.91E-11 | 1.62 | 7.19E-04 | 1.89 | 4.12E-05 | 5.43 | 2.94E-11 | 2.87 | 5.21E-08 |
|  | *NPEPPS* | 1.79 | 2.48E-04 | 2.23 | 7.01E-06 | 1.24 | 1.10E-01 | 1.22 | 1.45E-01 | 1.03 | 8.15E-01 | -1.18 | 2.15E-01 |
|  | *NRIP3* | 1.26 | 1.89E-01 | -1.03 | 8.67E-01 | -1.30 | 1.43E-01 | 1.37 | 8.35E-02 | 2.39 | 7.69E-05 | 1.75 | 4.40E-03 |
|  | *PEPD* | 1.60 | 1.20E-04 | 1.86 | 4.34E-06 | 1.17 | 1.29E-01 | -1.05 | 6.09E-01 | -1.20 | 7.60E-02 | -1.14 | 1.90E-01 |
|  | *POL3S* | -1.38 | 1.20E-02 | -1.19 | 1.44E-01 | 1.16 | 2.22E-01 | -1.05 | 6.70E-01 | 1.35 | 1.74E-02 | 1.42 | 6.87E-03 |
|  | *PPIL5* | 1.53 | 1.13E-04 | 1.21 | 4.50E-02 | -1.27 | 1.30E-02 | -1.19 | 5.91E-02 | -1.51 | 1.66E-04 | -1.27 | 1.41E-02 |
|  | *PSMA6* | 1.65 | 1.95E-03 | 1.81 | 4.48E-04 | 1.10 | 5.17E-01 | 1.04 | 7.60E-01 | -1.55 | 5.35E-03 | -1.62 | 2.70E-03 |
|  | *PSMD12* | -1.76 | 3.14E-03 | -1.79 | 2.70E-03 | -1.01 | 9.46E-01 | -1.50 | 2.51E-02 | -1.07 | 6.87E-01 | 1.40 | 5.69E-02 |
|  | *STAMBPL1* | 2.23 | 9.26E-05 | 2.05 | 3.00E-04 | -1.09 | 5.98E-01 | 1.41 | 4.54E-02 | 1.12 | 4.75E-01 | -1.26 | 1.73E-01 |
|  | *UBE2E1* | 1.88 | 2.91E-03 | 2.64 | 4.91E-05 | 1.41 | 8.00E-02 | 1.18 | 3.67E-01 | -1.14 | 4.93E-01 | -1.35 | 1.22E-01 |
|  | *UBE2G1* | 1.94 | 1.12E-03 | 2.30 | 1.27E-04 | 1.18 | 3.36E-01 | 1.20 | 2.94E-01 | -1.17 | 3.66E-01 | -1.41 | 5.97E-02 |
|  | *UBE2H* | 2.47 | 1.22E-04 | 3.30 | 4.73E-06 | 1.33 | 1.38E-01 | 1.47 | 5.26E-02 | 1.21 | 3.20E-01 | -1.22 | 3.07E-01 |
|  | *UBE2L6* | 1.48 | 2.10E-02 | 2.15 | 9.95E-05 | 1.46 | 2.53E-02 | 1.27 | 1.40E-01 | -1.00 | 9.83E-01 | -1.27 | 1.35E-01 |
|  | *USP48* | -1.98 | 2.71E-06 | -2.12 | 7.03E-07 | -1.07 | 4.87E-01 | -1.18 | 1.19E-01 | -1.01 | 8.99E-01 | 1.17 | 1.49E-01 |
|  | *YME1L1* | 2.52 | 5.77E-05 | 2.78 | 1.81E-05 | 1.10 | 5.89E-01 | 1.21 | 2.95E-01 | -1.02 | 9.03E-01 | -1.24 | 2.45E-01 |
|  |  |  |  |  |  |  |  |  |  |  |  |  |  |
| **Cell differentiation** | *CAST* | -1.18 | 2.47E-01 | -1.89 | 2.73E-04 | -1.60 | 3.90E-03 | -1.25 | 1.31E-01 | 1.12 | 4.36E-01 | 1.40 | 2.86E-02 |
|  | *CDK5RAP3* | -1.80 | 6.31E-04 | -1.57 | 5.43E-03 | 1.15 | 3.45E-01 | -1.27 | 1.16E-01 | -1.08 | 5.80E-01 | 1.17 | 2.91E-01 |
|  | *DFNA5* | 2.01 | 5.30E-06 | 2.25 | 7.51E-07 | 1.12 | 3.25E-01 | 1.12 | 3.19E-01 | 1.51 | 1.49E-03 | 1.35 | 1.41E-02 |
|  | *FNDC3B* | -1.33 | 2.12E-02 | -1.68 | 2.00E-04 | -1.27 | 4.79E-02 | 1.13 | 2.76E-01 | 1.05 | 6.70E-01 | -1.08 | 4.99E-01 |
|  | *IFRD1* | 1.83 | 4.42E-05 | 1.92 | 1.91E-05 | 1.05 | 6.95E-01 | -1.16 | 2.20E-01 | 1.04 | 7.61E-01 | 1.20 | 1.32E-01 |
|  | *KIF2A* | 1.55 | 2.94E-04 | 1.93 | 2.75E-06 | 1.24 | 3.86E-02 | 1.16 | 1.55E-01 | -1.05 | 6.06E-01 | -1.22 | 5.98E-02 |
|  | *MGST1* | 1.98 | 2.06E-05 | 1.87 | 5.39E-05 | -1.06 | 6.55E-01 | 1.26 | 6.54E-02 | -1.07 | 5.94E-01 | -1.35 | 2.21E-02 |
|  | *NAV1* | -1.54 | 8.95E-04 | -1.99 | 5.43E-06 | -1.29 | 2.81E-02 | -1.11 | 3.47E-01 | -1.11 | 3.38E-01 | -1.00 | 9.84E-01 |
|  | *NGRN* | 1.73 | 1.45E-03 | 2.12 | 6.82E-05 | 1.22 | 1.82E-01 | 1.50 | 1.28E-02 | 1.16 | 3.20E-01 | -1.29 | 9.86E-02 |
|  | *NTN4* | 1.12 | 2.96E-01 | -1.04 | 7.39E-01 | -1.16 | 1.74E-01 | 1.44 | 2.78E-03 | -1.07 | 5.17E-01 | -1.55 | 6.38E-04 |
|  | *OSGIN2* | 2.04 | 1.34E-03 | 2.13 | 7.79E-04 | 1.05 | 8.09E-01 | 1.32 | 1.57E-01 | 1.24 | 2.71E-01 | -1.07 | 7.36E-01 |
|  | *PAQR8* | -1.72 | 2.47E-05 | -1.72 | 2.55E-05 | 1.00 | 9.89E-01 | 1.10 | 3.28E-01 | 1.23 | 4.75E-02 | 1.11 | 2.77E-01 |
|  | *PHGDH* | 5.85 | 1.17E-08 | 5.78 | 1.30E-08 | -1.01 | 9.48E-01 | 1.77 | 5.22E-03 | 3.25 | 3.73E-06 | 1.83 | 3.38E-03 |
|  | *S100A4* | -1.28 | 1.15E-01 | -1.44 | 2.64E-02 | -1.12 | 4.56E-01 | -1.68 | 2.90E-03 | -2.02 | 1.80E-04 | -1.21 | 2.26E-01 |
|  | *SEMA4B* | -5.27 | 1.11E-12 | -4.90 | 2.37E-12 | 1.08 | 4.58E-01 | 1.03 | 7.39E-01 | 1.32 | 9.94E-03 | 1.28 | 2.04E-02 |
|  | *SLIT3* | -2.39 | 6.24E-09 | -2.77 | 5.49E-10 | -1.16 | 1.06E-01 | 1.23 | 2.53E-02 | 1.24 | 2.00E-02 | 1.01 | 9.11E-01 |
|  | *UPK1A* | -2.29 | 1.34E-09 | -2.05 | 1.33E-08 | 1.12 | 1.40E-01 | 1.77 | 3.52E-07 | 2.02 | 1.81E-08 | 1.14 | 9.71E-02 |
|  |  |  |  |  |  |  |  |  |  |  |  |  |  |
| **Immune system process** | *ADSSL1* | -3.67 | 3.12E-09 | -3.99 | 1.17E-09 | -1.09 | 5.03E-01 | 1.49 | 4.22E-03 | 1.23 | 1.02E-01 | -1.21 | 1.38E-01 |
|  | *EBI3* | -1.70 | 3.26E-06 | -1.79 | 9.27E-07 | -1.05 | 5.21E-01 | 1.54 | 4.07E-05 | 1.69 | 3.46E-06 | 1.10 | 2.44E-01 |
|  | *GBP2* | -1.50 | 4.54E-03 | -1.53 | 3.34E-03 | -1.02 | 8.91E-01 | -1.00 | 9.96E-01 | 1.42 | 1.17E-02 | 1.42 | 1.16E-02 |
|  | *HLA-A* | -2.00 | 9.05E-04 | -2.29 | 1.61E-04 | -1.15 | 4.45E-01 | -1.55 | 2.24E-02 | -1.08 | 6.71E-01 | 1.44 | 5.36E-02 |
|  | *IFI44L* | -4.77 | 2.03E-05 | -3.22 | 4.51E-04 | 1.48 | 1.68E-01 | -1.83 | 4.05E-02 | -1.83 | 4.00E-02 | -1.00 | 9.95E-01 |
|  | *IL18BP* | -1.92 | 4.51E-05 | -1.72 | 3.40E-04 | 1.12 | 3.65E-01 | -1.82 | 1.20E-04 | -1.34 | 2.98E-02 | 1.36 | 2.13E-02 |
|  | *LILRB1* | -2.35 | 4.03E-04 | -1.94 | 3.66E-03 | 1.22 | 3.34E-01 | -1.35 | 1.44E-01 | 1.15 | 5.00E-01 | 1.55 | 3.97E-02 |
|  | *LOC728666* | 1.83 | 1.94E-04 | 2.09 | 2.10E-05 | 1.14 | 3.13E-01 | 1.31 | 5.19E-02 | -1.14 | 3.12E-01 | -1.50 | 5.90E-03 |
|  | *NDRG1* | 1.01 | 9.68E-01 | 1.12 | 5.01E-01 | 1.12 | 5.27E-01 | 1.17 | 3.69E-01 | 1.67 | 7.94E-03 | 1.42 | 5.37E-02 |
|  | *OAS2* | -3.92 | 2.98E-06 | -3.11 | 2.93E-05 | 1.26 | 2.76E-01 | -2.94 | 5.29E-05 | -2.77 | 9.72E-05 | 1.06 | 7.81E-01 |
|  | *OASL* | -2.66 | 7.99E-07 | -2.42 | 3.25E-06 | 1.10 | 4.74E-01 | -1.30 | 6.34E-02 | 1.45 | 1.25E-02 | 1.88 | 1.59E-04 |
|  |  |  |  |  |  |  |  |  |  |  |  |  |  |
| **Nucleobase, nucleoside, nucleotide and nucleic acid metabolic process** | *AK3L1* | -1.55 | 1.44E-06 | -1.54 | 1.56E-06 | 1.00 | 9.66E-01 | 1.13 | 6.13E-02 | 1.65 | 2.06E-07 | 1.46 | 9.14E-06 |
|  | *C8orf45* | -2.66 | 4.10E-03 | -2.35 | 1.01E-02 | 1.13 | 6.84E-01 | -1.77 | 7.06E-02 | 1.13 | 6.86E-01 | 2.00 | 3.15E-02 |
|  | *CSTF1* | 1.54 | 8.91E-04 | 1.84 | 2.36E-05 | 1.20 | 1.12E-01 | 1.03 | 8.01E-01 | -1.09 | 4.12E-01 | -1.13 | 2.87E-01 |
|  | *CTPS2* | -1.72 | 5.36E-06 | -1.78 | 2.40E-06 | -1.04 | 6.89E-01 | 1.01 | 9.02E-01 | 1.10 | 2.67E-01 | 1.09 | 3.21E-01 |
|  | *DDX47* | 2.22 | 9.59E-04 | 2.53 | 2.29E-04 | 1.14 | 5.27E-01 | 1.35 | 1.56E-01 | -1.09 | 6.66E-01 | -1.47 | 7.10E-02 |
|  | *EMG1* | 1.36 | 2.08E-03 | 1.52 | 1.19E-04 | 1.12 | 2.12E-01 | 1.13 | 1.66E-01 | 1.11 | 2.49E-01 | -1.02 | 8.04E-01 |
|  | *EXOSC3* | 1.39 | 2.33E-02 | 1.45 | 1.16E-02 | 1.05 | 7.45E-01 | 1.11 | 4.54E-01 | -1.13 | 3.56E-01 | -1.26 | 1.04E-01 |
|  | *GART* | 1.92 | 3.66E-05 | 1.91 | 3.78E-05 | -1.00 | 9.88E-01 | 1.24 | 9.13E-02 | -1.10 | 4.48E-01 | -1.36 | 1.97E-02 |
|  | *KRR1* | 1.93 | 5.31E-05 | 2.13 | 1.10E-05 | 1.10 | 4.63E-01 | 1.01 | 9.42E-01 | -1.22 | 1.28E-01 | -1.23 | 1.13E-01 |
|  | *LARP6* | 2.51 | 5.09E-07 | 2.46 | 6.90E-07 | -1.02 | 8.71E-01 | 1.04 | 7.30E-01 | 1.32 | 3.65E-02 | 1.26 | 7.24E-02 |
|  | *LOC100130932* | 1.54 | 2.14E-03 | 1.62 | 9.19E-04 | 1.05 | 7.09E-01 | 1.17 | 2.06E-01 | -1.15 | 2.73E-01 | -1.35 | 2.51E-02 |
|  | *LRRC33* | 1.42 | 5.90E-04 | 1.63 | 1.65E-05 | 1.15 | 1.15E-01 | -1.61 | 2.12E-05 | -1.32 | 3.66E-03 | 1.22 | 2.99E-02 |
|  | *PANK2* | 1.55 | 4.99E-03 | 1.34 | 4.62E-02 | -1.16 | 3.05E-01 | 1.05 | 7.29E-01 | -1.37 | 3.60E-02 | -1.43 | 1.74E-02 |
|  | *PNPT1* | -2.18 | 1.43E-03 | -1.95 | 4.70E-03 | 1.12 | 5.98E-01 | -1.55 | 4.86E-02 | 1.06 | 7.70E-01 | 1.65 | 2.68E-02 |
|  | *REC8* | -2.23 | 5.96E-04 | -1.86 | 4.88E-03 | 1.20 | 3.56E-01 | -1.46 | 6.77E-02 | -1.19 | 3.70E-01 | 1.22 | 3.20E-01 |
|  | *RNASE4* | -1.63 | 1.24E-04 | -1.61 | 1.62E-04 | 1.01 | 9.05E-01 | 1.32 | 1.34E-02 | 1.25 | 3.95E-02 | -1.05 | 6.09E-01 |
|  | *RRM2* | 2.48 | 2.81E-05 | 1.75 | 2.92E-03 | -1.41 | 4.82E-02 | 1.11 | 5.21E-01 | -1.18 | 3.26E-01 | -1.31 | 1.13E-01 |
|  | *RTCD1* | 1.78 | 1.48E-03 | 1.89 | 6.03E-04 | 1.06 | 6.91E-01 | 1.66 | 4.10E-03 | 1.36 | 5.96E-02 | -1.22 | 2.18E-01 |
|  | *SNURF* | 1.82 | 1.64E-04 | 2.19 | 7.51E-06 | 1.20 | 1.62E-01 | 1.41 | 1.41E-02 | -1.04 | 7.48E-01 | -1.47 | 6.99E-03 |
|  | *TDO2* | 2.25 | 3.96E-06 | 2.80 | 1.47E-07 | 1.25 | 9.35E-02 | 1.43 | 9.57E-03 | 1.07 | 5.95E-01 | -1.34 | 2.99E-02 |
|  | *TINF2* | 1.56 | 1.54E-02 | 1.86 | 1.53E-03 | 1.19 | 3.05E-01 | 1.34 | 9.81E-02 | 1.13 | 4.80E-01 | -1.19 | 3.19E-01 |
|  | *TSEN15* | 2.08 | 1.15E-04 | 1.68 | 2.70E-03 | -1.24 | 1.71E-01 | 1.16 | 3.25E-01 | -1.17 | 3.06E-01 | -1.36 | 5.35E-02 |
|  | *WTAP* | 2.16 | 3.29E-05 | 2.56 | 2.82E-06 | 1.18 | 2.44E-01 | 1.06 | 6.88E-01 | -1.17 | 2.79E-01 | -1.24 | 1.45E-01 |
|  |  |  |  |  |  |  |  |  |  |  |  |  |  |
| **Protein metabolic process** | *CCT6A* | 1.93 | 1.80E-03 | 2.40 | 1.23E-04 | 1.24 | 2.40E-01 | 1.47 | 4.64E-02 | -1.09 | 6.20E-01 | -1.61 | 1.65E-02 |
|  | *CCT7* | 1.68 | 3.29E-03 | 2.04 | 1.95E-04 | 1.22 | 2.19E-01 | 1.31 | 9.84E-02 | -1.20 | 2.55E-01 | -1.56 | 9.17E-03 |
|  | *FKBP10* | -2.42 | 5.64E-06 | -2.48 | 3.95E-06 | -1.03 | 8.61E-01 | -1.23 | 1.55E-01 | -1.11 | 4.67E-01 | 1.11 | 4.67E-01 |
|  | *LOC402644* | 1.80 | 1.35E-02 | 1.82 | 1.22E-02 | 1.01 | 9.64E-01 | 1.20 | 4.02E-01 | -1.32 | 2.07E-01 | -1.59 | 4.39E-02 |
|  | *MCRS1* | 1.50 | 4.23E-04 | 1.86 | 3.76E-06 | 1.24 | 3.82E-02 | 1.11 | 3.00E-01 | -1.12 | 2.34E-01 | -1.24 | 3.36E-02 |
|  | *NKTR* | -1.93 | 9.13E-06 | -1.53 | 9.66E-04 | 1.26 | 4.40E-02 | -1.18 | 1.38E-01 | 1.11 | 3.64E-01 | 1.31 | 2.31E-02 |
|  | *P4HA2* | -1.67 | 1.98E-05 | -1.84 | 2.22E-06 | -1.10 | 2.91E-01 | 1.13 | 1.86E-01 | 1.17 | 9.27E-02 | 1.04 | 6.92E-01 |
|  | *P4HB* | -1.64 | 1.04E-06 | -2.04 | 4.59E-09 | -1.25 | 4.65E-03 | -1.08 | 2.71E-01 | -1.04 | 5.85E-01 | 1.04 | 5.70E-01 |
|  | *SGSH* | -1.79 | 7.32E-04 | -1.87 | 3.47E-04 | -1.05 | 7.41E-01 | -1.30 | 8.36E-02 | -1.28 | 9.99E-02 | 1.01 | 9.24E-01 |
|  | *TCP1* | 3.00 | 6.13E-05 | 3.53 | 1.22E-05 | 1.18 | 4.52E-01 | 1.42 | 1.17E-01 | -1.11 | 6.31E-01 | -1.57 | 4.66E-02 |
|  |  |  |  |  |  |  |  |  |  |  |  |  |  |
| **Metabolic process** | *AMD1* | 1.72 | 9.24E-06 | 2.12 | 1.03E-07 | 1.24 | 2.74E-02 | 1.04 | 6.64E-01 | -1.21 | 4.34E-02 | -1.26 | 1.76E-02 |
|  | *AMT* | -1.97 | 5.96E-04 | -1.74 | 3.18E-03 | 1.13 | 4.62E-01 | -1.19 | 2.95E-01 | 1.02 | 9.13E-01 | 1.21 | 2.50E-01 |
|  | *ARSD* | -1.93 | 2.20E-07 | -1.71 | 3.51E-06 | 1.13 | 1.56E-01 | -1.10 | 2.37E-01 | -1.38 | 8.52E-04 | -1.25 | 1.26E-02 |
|  | *ASMTL* | -1.73 | 7.40E-04 | -1.27 | 9.72E-02 | 1.36 | 3.31E-02 | 1.14 | 3.31E-01 | 1.12 | 4.12E-01 | -1.02 | 8.75E-01 |
|  | *BCAT1* | 2.51 | 1.31E-05 | 2.23 | 6.40E-05 | -1.12 | 4.60E-01 | 1.02 | 9.14E-01 | 1.04 | 8.22E-01 | 1.02 | 9.07E-01 |
|  | *C12orf5* | 2.69 | 4.15E-05 | 2.51 | 9.17E-05 | -1.07 | 7.16E-01 | -1.05 | 8.05E-01 | -1.29 | 1.82E-01 | -1.23 | 2.70E-01 |
|  | *CENPV* | 1.55 | 2.59E-03 | 1.94 | 5.34E-05 | 1.25 | 9.48E-02 | 1.06 | 6.42E-01 | 1.26 | 8.14E-02 | 1.19 | 1.87E-01 |
|  | *CKS1B* | 1.80 | 1.35E-04 | 1.48 | 4.97E-03 | -1.22 | 1.21E-01 | 1.07 | 5.75E-01 | 1.04 | 7.30E-01 | -1.03 | 8.27E-01 |
|  | *CLYBL* | -1.45 | 9.90E-05 | -1.49 | 4.51E-05 | -1.03 | 7.19E-01 | 1.49 | 4.86E-05 | 1.31 | 2.24E-03 | -1.14 | 9.87E-02 |
|  | *CSAD* | -1.32 | 2.07E-02 | -1.35 | 1.46E-02 | -1.02 | 8.69E-01 | -1.15 | 2.34E-01 | 1.17 | 1.70E-01 | 1.34 | 1.60E-02 |
|  | *ECHDC1* | 1.77 | 4.24E-04 | 1.75 | 5.47E-04 | -1.02 | 9.10E-01 | 1.12 | 4.12E-01 | -1.17 | 2.47E-01 | -1.31 | 5.67E-02 |
|  | *FTSJD1* | 1.81 | 5.77E-04 | 1.97 | 1.67E-04 | 1.08 | 5.82E-01 | 1.04 | 8.04E-01 | -1.44 | 2.01E-02 | -1.49 | 1.18E-02 |
|  | *GSTM2* | -1.90 | 1.08E-05 | -1.82 | 2.38E-05 | 1.04 | 7.08E-01 | 1.17 | 1.54E-01 | 1.25 | 5.38E-02 | 1.06 | 5.73E-01 |
|  | *IDS* | 2.59 | 1.82E-07 | 2.85 | 4.44E-08 | 1.10 | 4.19E-01 | 1.07 | 5.51E-01 | 1.21 | 1.13E-01 | 1.13 | 3.03E-01 |
|  | *ISCU* | 1.77 | 3.05E-07 | 1.91 | 5.01E-08 | 1.08 | 3.10E-01 | 1.19 | 2.81E-02 | -1.09 | 2.57E-01 | -1.29 | 2.24E-03 |
|  | *LEPRE1* | -1.95 | 1.52E-06 | -2.48 | 1.85E-08 | -1.27 | 2.12E-02 | -1.05 | 6.27E-01 | -1.10 | 3.27E-01 | -1.05 | 6.15E-01 |
|  | *LEPREL1* | -1.78 | 1.56E-07 | -2.12 | 2.68E-09 | -1.19 | 2.02E-02 | 1.30 | 1.60E-03 | 1.03 | 6.80E-01 | -1.26 | 4.05E-03 |
|  | *LEPREL2* | -3.29 | 5.34E-07 | -3.61 | 1.86E-07 | -1.10 | 5.65E-01 | -1.05 | 7.54E-01 | -1.18 | 3.07E-01 | -1.12 | 4.73E-01 |
|  | *LGSN* | -2.30 | 8.30E-04 | -2.07 | 2.61E-03 | 1.11 | 6.12E-01 | -1.47 | 7.97E-02 | 1.52 | 6.12E-02 | 2.23 | 1.16E-03 |
|  | *ME2* | 1.88 | 2.10E-04 | 2.16 | 2.25E-05 | 1.15 | 3.11E-01 | 1.33 | 5.05E-02 | -1.04 | 7.63E-01 | -1.39 | 2.73E-02 |
|  | *MTHFD2* | 1.69 | 4.27E-07 | 1.82 | 6.27E-08 | 1.08 | 2.87E-01 | 1.16 | 4.71E-02 | 1.52 | 9.07E-06 | 1.31 | 8.88E-04 |
|  | *NEU1* | 1.89 | 1.57E-05 | 2.39 | 2.32E-07 | 1.27 | 4.17E-02 | 1.13 | 2.79E-01 | 1.09 | 4.16E-01 | -1.03 | 7.80E-01 |
|  | *PCYOX1L* | -1.53 | 3.27E-04 | -1.81 | 7.52E-06 | -1.18 | 9.31E-02 | 1.43 | 1.55E-03 | 1.08 | 4.46E-01 | -1.32 | 8.65E-03 |
|  | *PPA2* | -2.70 | 1.97E-03 | -2.15 | 1.20E-02 | 1.25 | 4.20E-01 | -1.77 | 5.19E-02 | 1.06 | 8.27E-01 | 1.88 | 3.33E-02 |
|  | *PSAT1* | 4.91 | 2.34E-11 | 4.61 | 4.49E-11 | -1.06 | 5.84E-01 | 1.72 | 1.05E-04 | 2.04 | 4.13E-06 | 1.19 | 1.37E-01 |
|  | *PSPH* | 1.18 | 7.66E-02 | 1.33 | 4.21E-03 | 1.13 | 1.80E-01 | 1.63 | 2.58E-05 | -1.21 | 4.28E-02 | -1.98 | 3.64E-07 |
|  | *PTS* | 1.70 | 1.41E-03 | 1.65 | 2.24E-03 | -1.03 | 8.38E-01 | 1.23 | 1.64E-01 | -1.17 | 2.73E-01 | -1.44 | 1.88E-02 |
|  | *RDH10* | 2.22 | 9.53E-06 | 2.36 | 3.63E-06 | 1.07 | 6.36E-01 | 1.04 | 7.64E-01 | -1.10 | 4.61E-01 | -1.15 | 3.04E-01 |
|  | *SAPS3* | -1.94 | 1.03E-04 | -2.01 | 5.93E-05 | -1.03 | 8.03E-01 | -1.11 | 4.49E-01 | -1.06 | 6.80E-01 | 1.05 | 7.27E-01 |
|  | *SDC4* | -2.02 | 1.04E-04 | -2.14 | 4.24E-05 | -1.06 | 6.80E-01 | 1.12 | 4.38E-01 | 1.73 | 1.12E-03 | 1.55 | 6.51E-03 |
|  | *UPB1* | -1.58 | 9.34E-05 | -1.61 | 5.70E-05 | -1.02 | 8.21E-01 | 1.12 | 2.21E-01 | -1.24 | 2.82E-02 | -1.40 | 1.81E-03 |
|  |  |  |  |  |  |  |  |  |  |  |  |  |  |
| **Regulation of cell growth** | *CAPRIN2* | -1.49 | 5.72E-03 | -1.23 | 1.29E-01 | 1.22 | 1.40E-01 | -1.20 | 1.76E-01 | 1.41 | 1.52E-02 | 1.69 | 6.86E-04 |
|  | *CRIM1* | 1.11 | 6.68E-01 | -1.13 | 6.18E-01 | -1.25 | 3.58E-01 | 1.60 | 6.37E-02 | -1.44 | 1.41E-01 | -2.31 | 2.47E-03 |
|  | *ESM1* | 2.95 | 1.73E-07 | 2.05 | 3.48E-05 | -1.44 | 1.35E-02 | 1.54 | 4.37E-03 | 1.61 | 2.10E-03 | 1.04 | 7.46E-01 |
|  | *FAM107A* | -1.51 | 1.93E-05 | -1.95 | 2.45E-08 | -1.29 | 1.95E-03 | -1.61 | 2.69E-06 | -1.95 | 2.39E-08 | -1.21 | 1.60E-02 |
|  | *FGFRL1* | -1.43 | 1.74E-02 | -1.49 | 9.48E-03 | -1.04 | 7.79E-01 | 1.07 | 6.32E-01 | 1.58 | 3.61E-03 | 1.48 | 1.05E-02 |
|  |  |  |  |  |  |  |  |  |  |  |  |  |  |
| **Multicellular organism development** | *C11orf73* | 1.62 | 7.04E-04 | 1.84 | 6.33E-05 | 1.14 | 2.87E-01 | 1.16 | 2.14E-01 | -1.31 | 3.65E-02 | -1.52 | 2.30E-03 |
|  | *C1QTNF5* | -1.87 | 6.09E-04 | -1.90 | 4.72E-04 | -1.02 | 9.10E-01 | -1.64 | 4.01E-03 | -2.26 | 3.94E-05 | -1.37 | 4.98E-02 |
|  | *KRT10* | 1.94 | 3.41E-04 | 1.86 | 6.65E-04 | -1.05 | 7.67E-01 | 1.17 | 3.00E-01 | -1.01 | 9.39E-01 | -1.19 | 2.67E-01 |
|  | *KRT15* | -1.37 | 9.44E-04 | -1.55 | 2.70E-05 | -1.14 | 1.20E-01 | 1.09 | 3.12E-01 | 1.99 | 7.33E-08 | 1.83 | 4.56E-07 |
|  | *LMBR1* | 1.75 | 1.39E-05 | 2.24 | 1.02E-07 | 1.28 | 1.81E-02 | -1.07 | 4.89E-01 | -1.53 | 2.77E-04 | -1.43 | 1.33E-03 |
|  | *MFNG* | -2.15 | 4.20E-07 | -2.14 | 4.52E-07 | 1.00 | 9.68E-01 | -1.30 | 1.62E-02 | -1.54 | 4.02E-04 | -1.18 | 1.11E-01 |
|  | *NAV2* | -1.28 | 1.22E-01 | -1.27 | 1.32E-01 | 1.01 | 9.63E-01 | -1.06 | 7.07E-01 | 2.32 | 3.08E-05 | 2.46 | 1.39E-05 |
|  |  |  |  |  |  |  |  |  |  |  |  |  |  |
| **Cell cycle** | *DMWD* | -1.73 | 2.49E-03 | -1.65 | 4.90E-03 | 1.05 | 7.63E-01 | -1.47 | 2.38E-02 | -1.06 | 6.90E-01 | 1.38 | 5.37E-02 |
|  | *FAM83D* | 1.39 | 1.79E-01 | 1.30 | 2.77E-01 | -1.07 | 7.85E-01 | -1.42 | 1.53E-01 | -2.31 | 2.32E-03 | -1.62 | 5.50E-02 |
|  | *KNTC1* | -1.29 | 1.84E-02 | -1.71 | 3.23E-05 | -1.33 | 9.49E-03 | -1.12 | 2.80E-01 | 1.45 | 1.34E-03 | 1.62 | 1.15E-04 |
|  | *MAD2L1BP* | 1.47 | 7.43E-03 | 1.90 | 9.41E-05 | 1.29 | 6.34E-02 | 1.02 | 8.91E-01 | -1.26 | 9.09E-02 | -1.28 | 7.02E-02 |
|  | *MPHOSPH6* | 1.74 | 2.28E-05 | 1.65 | 7.04E-05 | -1.05 | 6.02E-01 | 1.05 | 6.04E-01 | -1.15 | 1.66E-01 | -1.21 | 6.41E-02 |
|  | *NUMA1* | -1.79 | 7.49E-05 | -2.29 | 9.23E-07 | -1.28 | 4.32E-02 | -1.25 | 6.68E-02 | 1.16 | 2.08E-01 | 1.45 | 4.37E-03 |
|  | *RBM7* | 1.71 | 5.29E-04 | 2.14 | 1.25E-05 | 1.25 | 9.86E-02 | 1.39 | 1.82E-02 | 1.03 | 8.26E-01 | -1.35 | 2.88E-02 |
|  | *SESN2* | 1.45 | 7.79E-03 | 1.35 | 2.73E-02 | -1.08 | 5.62E-01 | -1.16 | 2.52E-01 | 1.09 | 4.75E-01 | 1.27 | 7.19E-02 |
|  |  |  |  |  |  |  |  |  |  |  |  |  |  |
| **other** | *CDV3* | 2.20 | 2.25E-05 | 1.85 | 3.21E-04 | -1.19 | 2.34E-01 | 1.44 | 1.67E-02 | 1.03 | 8.46E-01 | -1.40 | 2.52E-02 |
|  | *CMTM7* | 1.77 | 2.62E-03 | 2.55 | 2.14E-05 | 1.44 | 4.11E-02 | 1.15 | 4.11E-01 | -1.06 | 7.47E-01 | -1.21 | 2.58E-01 |
|  | *CRTAP* | 2.34 | 2.47E-04 | 2.48 | 1.24E-04 | 1.06 | 7.57E-01 | 1.54 | 3.17E-02 | 1.15 | 4.57E-01 | -1.34 | 1.34E-01 |
|  | *DNAH1* | -1.68 | 3.56E-03 | -1.35 | 6.96E-02 | 1.25 | 1.72E-01 | -1.31 | 9.82E-02 | -1.11 | 5.19E-01 | 1.18 | 2.92E-01 |
|  | *EFEMP1* | -1.73 | 6.75E-05 | -2.18 | 7.85E-07 | -1.26 | 4.00E-02 | 1.06 | 6.05E-01 | -1.35 | 1.04E-02 | -1.43 | 3.30E-03 |
|  | *GDF3* | -2.20 | 3.86E-06 | -2.23 | 3.12E-06 | -1.01 | 9.15E-01 | -2.54 | 4.00E-07 | -2.75 | 1.20E-07 | -1.09 | 5.05E-01 |
|  | *GLRX3* | 2.28 | 3.80E-05 | 2.34 | 2.73E-05 | 1.02 | 8.78E-01 | 1.29 | 1.17E-01 | -1.09 | 5.67E-01 | -1.40 | 3.85E-02 |
|  | *H1F0* | -1.36 | 6.69E-03 | -1.52 | 5.59E-04 | -1.12 | 2.78E-01 | -1.13 | 2.37E-01 | 1.27 | 2.67E-02 | 1.44 | 1.88E-03 |
|  | *H1FX* | -1.39 | 2.17E-02 | -1.60 | 2.25E-03 | -1.15 | 3.11E-01 | -1.08 | 5.71E-01 | 1.40 | 1.96E-02 | 1.51 | 5.67E-03 |
|  | *H3F3B* | -1.23 | 1.24E-01 | -1.52 | 5.08E-03 | -1.23 | 1.33E-01 | 1.08 | 5.55E-01 | 1.85 | 1.67E-04 | 1.71 | 6.29E-04 |
|  | *HIST1H2AC* | 2.01 | 7.90E-04 | 2.23 | 2.22E-04 | 1.11 | 5.73E-01 | 1.24 | 2.36E-01 | -1.15 | 4.36E-01 | -1.42 | 5.82E-02 |
|  | *HNMT* | 2.04 | 3.95E-06 | 2.37 | 2.94E-07 | 1.16 | 1.85E-01 | 1.17 | 1.70E-01 | 1.15 | 2.15E-01 | -1.02 | 8.87E-01 |
|  | *HPS1* | 1.95 | 2.11E-06 | 1.85 | 5.91E-06 | -1.05 | 6.07E-01 | -1.05 | 6.13E-01 | 1.08 | 4.20E-01 | 1.14 | 1.97E-01 |
|  | *LOC653557* | 1.59 | 2.23E-03 | 1.87 | 1.29E-04 | 1.18 | 2.14E-01 | 1.28 | 7.29E-02 | 1.03 | 8.47E-01 | -1.25 | 1.05E-01 |
|  | *LXN* | -1.70 | 1.48E-03 | -1.55 | 6.14E-03 | 1.09 | 5.28E-01 | 2.05 | 7.54E-05 | 1.89 | 2.74E-04 | -1.09 | 5.62E-01 |
|  | *M6PR* | 2.21 | 2.98E-04 | 2.80 | 1.74E-05 | 1.27 | 2.03E-01 | 1.19 | 3.37E-01 | 1.01 | 9.53E-01 | -1.18 | 3.66E-01 |
|  | *MT1X* | -1.24 | 3.49E-03 | 1.02 | 7.30E-01 | 1.27 | 1.60E-03 | 1.71 | 1.62E-07 | 1.32 | 4.21E-04 | -1.29 | 9.99E-04 |
|  | *PPFIA4* | -1.70 | 1.23E-05 | -1.70 | 1.32E-05 | 1.00 | 9.75E-01 | 1.04 | 6.32E-01 | 1.32 | 6.08E-03 | 1.26 | 1.73E-02 |
|  | *PSMG1* | 1.62 | 1.78E-05 | 1.68 | 7.52E-06 | 1.04 | 6.80E-01 | 1.06 | 4.90E-01 | -1.23 | 2.24E-02 | -1.31 | 4.93E-03 |
|  | *SNX33* | -1.59 | 2.88E-04 | -1.64 | 1.46E-04 | -1.03 | 7.61E-01 | 1.14 | 2.34E-01 | 1.26 | 3.72E-02 | 1.11 | 3.23E-01 |
|  | *SPAG4* | -3.16 | 1.63E-09 | -3.12 | 1.99E-09 | 1.01 | 8.92E-01 | 1.48 | 1.30E-03 | 1.39 | 4.99E-03 | -1.06 | 5.52E-01 |
|  | *STOM* | 2.28 | 4.22E-03 | 3.32 | 1.54E-04 | 1.46 | 1.53E-01 | 1.10 | 7.03E-01 | -1.67 | 5.76E-02 | -1.84 | 2.65E-02 |
|  | *TMEM91* | -2.24 | 1.68E-03 | -2.17 | 2.33E-03 | 1.03 | 8.85E-01 | -1.51 | 7.47E-02 | 1.00 | 9.87E-01 | 1.52 | 7.25E-02 |
|  | *WDR1* | 2.36 | 5.87E-05 | 2.77 | 7.76E-06 | 1.17 | 3.45E-01 | 1.06 | 7.24E-01 | 1.11 | 5.38E-01 | 1.05 | 7.91E-01 |

FC: Fold-Change; 1g: Adherent cells cultured in a normal laboratory incubator; AD: Adherent cells in simulated µg; 3D: Elongated three dimensional cell aggregates in simulated µg
